# *n*‑Butane Capture Accompanied by a Colorimetric Response in a Flexible Thiazolothiazole Metal–Organic Framework

**DOI:** 10.1021/jacs.6c10210

**Published:** 2026-09-07

**Authors:** Szymon K. Sobczak, Asif Raza, Bartosz Mazur, Wola Mishchanka, Anastasia Efimova, Volodymyr Bon, Bogdan Kuchta, Michael J. Zaworotko, Stefan Kaskel, Soumya Mukherjee, Kornel Roztocki, Agnieszka M. Janiak

**Affiliations:** † Faculty of Chemistry, 49562 Adam Mickiewicz University, Uniwersytetu Poznańskiego 8, 61-614 Poznań, Poland; ‡ Department of Chemical Sciences, Bernal Institute and Research Ireland Centre for Pharmaceuticals (SSPC), 8808 University of Limerick, Limerick V94 T9PX, Ireland; § Faculty of Chemistry, Wrocław University of Science and Technology, C. K. Norwida 4/6, Wrocław 50-370, Poland; ∥ Faculty of Agriculture/Environment/Chemistry, Dresden University of Applied Sciences, Friedrich-List-Platz 1, 01069 Dresden, Germany; ⊥ Chair of Inorganic Chemistry, 9169 Technische Universität Dresden, Bergstrasse 66, 01062 Dresden, Germany

## Abstract

Simultaneous capture and optical detection of flammable gases in enclosed environments are critical for operational safety. Herein, using two dynamic MOFs, UAM-1S and UAM-1O, which differ compositionally by a single atom, we demonstrate the crucial role of framework flexibility in enabling this dual function. The mechanistic, kinetic, and energetic landscape of *n*-butane-induced structural transformations is revealed by *in situ* UV–vis spectroscopy and static/time-resolved powder X-ray diffraction conducted during adsorption along calorimetric studies. The integration of these results with single-crystal X-ray diffraction data of UAM-1O loaded with hydrocarbons, together with grand canonical Monte Carlo simulations and DFT calculations, enables us to propose a diffusion mechanism. Furthermore, isothermal adsorption measurements show that UAM-1S adsorbs 1.5 mmol g^–1^ of *n-*butane at 298 K and 0.016 bar, below its explosive limit (1.9%), while exhibiting minimal water uptake. Complementary dynamic column breakthrough and temperature-programmed desorption experiments reveal the selective capture of *n-*butane from simulated air at concentrations below 1.9% under dry conditions and below 3% under humid conditions. Finally, the loading of *n*-butane leads to a distinct color change of the host material thereby enabling its use as a visual indicator. These results open a new research direction, highlighting the untapped potential of flexible MOFs for capturing and detecting flammable gases at low concentrations.

## Graphical abstract

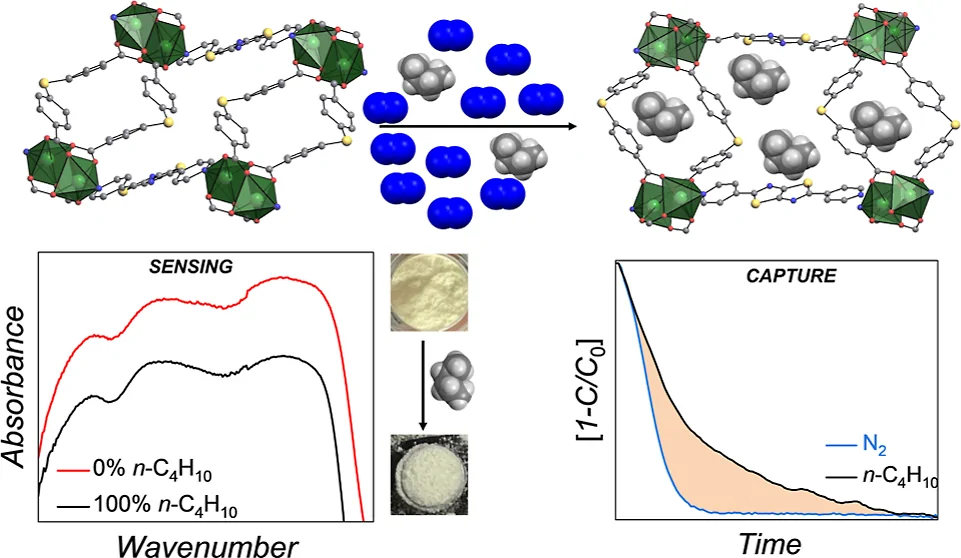

## Introduction

Ensuring operational safety in enclosed industrial, commercial, and domestic environments is critically important, as gas accumulation may lead to explosion or fire hazards. This challenge requires the development of advanced functional materials capable of simultaneous real-time air quality monitoring and rapid sequestration of hazardous gases. The integration of such multiple functionalities within a single material platform represents a major scientific and technological challenge.^1^ In this context, structural adaptivity and dynamic responsiveness, as exemplified by biological systems, are critical design principles,^2^ enabling effective multitasking performance.^3,4^

Among adaptive programmable materials,^5–7^ flexible metal–organic frameworks^8^ (MOFs), a rare subclass^9^ of MOFs, have emerged as systems capable of molecular recognition^10^ and selective expulsion.^11^ Typically, solvent removal from the nanocavities of flexible MOFs transforms their porous structure (open phase = op) into a non*-*porous or less porous form (closed phase = cp).^8^ Gas adsorption can initiate reopening of the MOF and in most of the cases the associated energy landscape characterized by a discontinuous (stepwise) profile with well-defined energy barriers between cp and op states.^12,13^ For example, ELM-11 exhibits a one-step structural transformation upon exposure to CO_2_ at 293 K,^14^ and two step during the CO_2_ adsorption at 195 K.^15^ On the other hand, JUK-8 transforms directly from a less porous form to the open form during CO_2_ adsorption at 195 K.^16^ Nevertheless, flexible porous materials that exhibit a continuous phase transformation with a steady increase in uptake are very rare with fewer than 10 examples reported to date.^17–21^ For example, SHF-61 incrementally changes its unit-cell volume during time-resolved desolvation,^18^ while X-dia-4-Co gradually transforms from the closed to the open phase when exposed to carbon dioxide at 195 K.^20^ Another example of such behavior is UAM-1S, which incrementally changes its structure in response to CO_2_ at 195 K.^17,21^

To leverage the full potential of flexible MOFs, it is crucial to develop a detailed experimental and theoretical understanding of the mechanisms driving the structural transformations in time domain,^22^ together with high-throughput screening methods.^23,24^ This involves studying the thermodynamic and kinetic aspects of phase changes, the role of host–guest interactions, and the influence of external conditions such as temperature and pressure.^13^ Advanced characterization techniques, computational modeling, and *in situ* monitoring are essential tools in this endeavor.^25^ This stems from the fact that structural adaptivity paves the way for arange of new-generation applications,^26–28^ particularly in gas storage^17,29,30^ and complex separations.^14,31–34^ The potential of flexible MOFs is demonstrated by separation of water isotopologues^35^ or propylene/propane mixtures,^36^ achieving highly efficient discrimination of xylene isomers,^37^ reaching record C_2_H_2_ packing densities,^38^ and capturing I_2_/CH_3_I in humid environments,^24^ among others. However, a single flexible material capable of acting as a multifunctional host for the simultaneous capture and detection of flammable gases at low concentrations is rare.

To address this gap, we investigate the capture and detection properties of two isostructural flexible thiazolo­[5,4-*d*]­thiazole MOFs, UAM-1O and UAM-1S,^17,21^ toward *n-*C_4_H_10_under various conditions. Previous studies reported that a minimal structural variation such as substitution of a single atom (oxygen *vs* sulfur) in the angular dicarboxylate linker changes the type of guest-induced response from the stepwise CO_2_-induced transformation in UAM-1O into a continuous transformation in UAM-1S. Here, we not only show that the structural transformation in both cases proceeds in a stepwise manner but also demonstrate that UAM-1S is a multifunctional material capable of capturing *n-*butane and undergoing a colorimetric response below the *n-*butane explosion limit. *In situ* UV–vis spectroscopy combined with equilibrium and time-resolved PXRD measurements during adsorption, along with differential scanning calorimetry of desorption, provides detailed insight into the mechanistic, kinetic, and energetic landscape of gas-induced structural transformations. While grand canonical Monte Carlo simulations (GCMC) and single-crystal structure of *n*-butane-loaded host enable the proposal of the diffusion mechanism. Furthermore, the isothermal adsorption measurement shows that UAM-1S adsorbs 1.5 mmol g^–1^ of *n-*C_4_H_10_ at 298 K and 0.016 bar, well below the explosive limit (1.9%). Complementary dynamic column breakthrough and temperature-programmed desorption experiments reveal selective capture of dilute *n-*butane from simulated *n-*C_4_H_10_/air mixtures at concentrations below 1.9% under dry conditions and below 3% under humid conditions. Concomitantly, exposure to *n-*butane induces structural changes accompanied by a distinct color change of the UAM-1S powder from yellow to almost white, which may be used as a visual indicator.

These results demonstrate that flexible MOFs are promising materials for a wide range of environmental applications. Their unique combination of low-concentration gas capture and a distinct colorimetric response driven by structural changes makes them particularly attractive compared to their rigid counterparts.

## Results and Discussion

### *n*-C_4_H_10_ Sorption

To provide a comprehensive overview of the properties of UAM-1X (X = O and S), we summarize key structural changes occurring upon desolvation^17,21^ (Figure 1). In the case of UAM-1O, two Zn–O bonds in the metal cluster break, transforming the cluster from a paddlewheel into two tetrahedra connected by two carboxylate groups. This transformation coincides with a substantial reduction in theoretical pore volume, from 37.4% to 5.1%. In contrast, desolvation of UAM-1S leaves the metal cluster intact, although the pore volume decreases from 36.1% to 6.2%.

**Figure 1:**
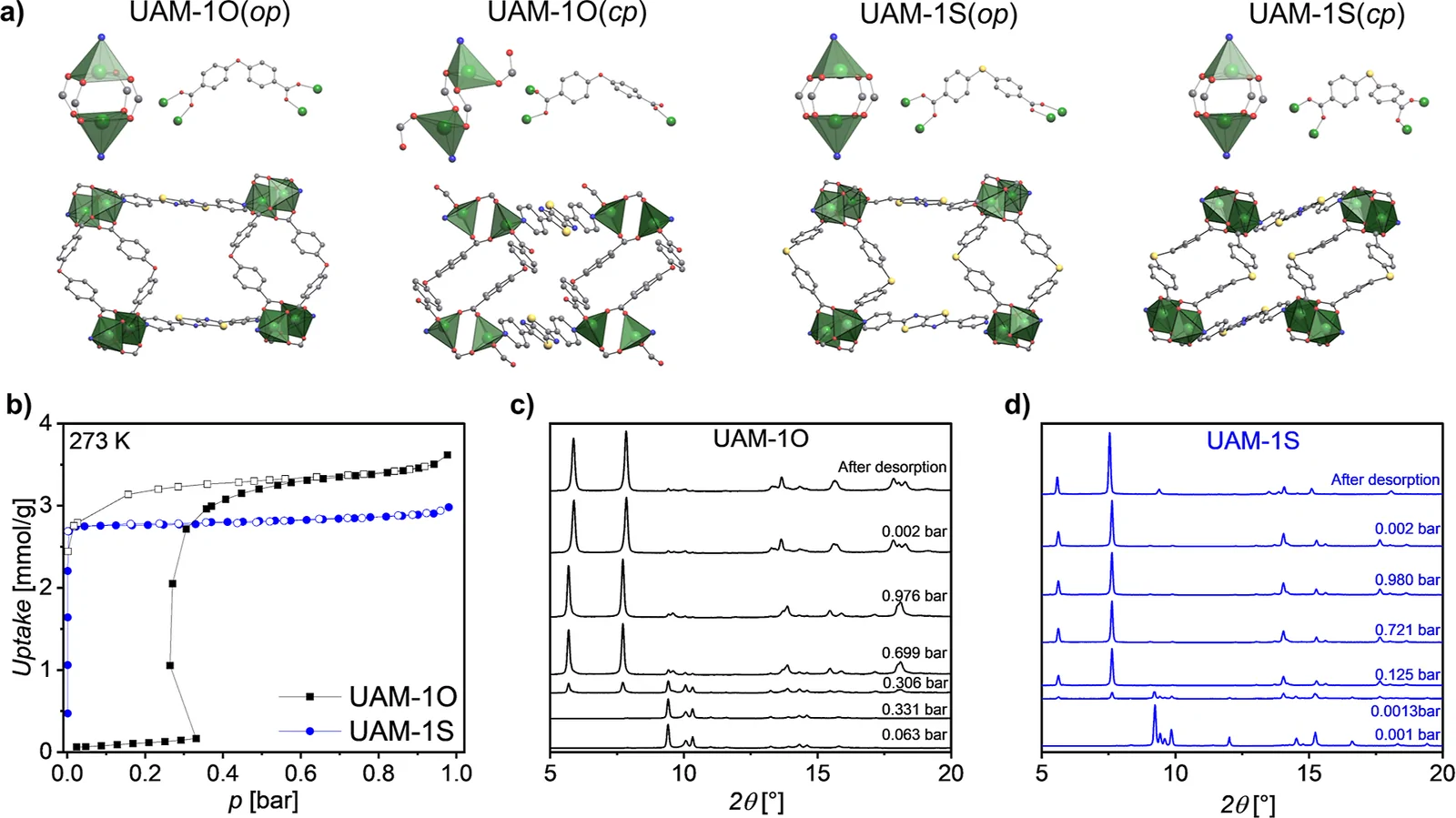
(a) Comparison of the crystal structures of the op and cp phases of UAM-1X (X = O or S); hydrogen atoms have been omitted for clarity and atoms are marked as follows: green—Zn, gray—C, red—O, blue—N, and yellow—S. Adapted with permission Copyright 2025 American Chemical Society.^21^ (b) *n*-Butane adsorption isotherms at 273 K for UAM-1O and UAM-1S (full symbols, adsorption; empty symbols, desorption) along with corresponding powder diffraction patterns of (c) UAM-1O and (d) UAM-1S collected at defined pressure.

In a previous report, we showed that UAM-1O exhibits a structural transition known as gate opening in response to the adsorption of CO_2_ at 195 K. The transition at 0.33 bar is preceded by moderate CO_2_ uptake of 20 cm^3^ g^–1^ (0.89 mmol g^–1^) by the cp-phase (Figure S1). On the other hand, *n-*butane at 273 K is not adsorbed by UAM-1O­(cp) until the pressure exceeds 0.33 bar, at which point a cp-op transition occurs, accompanied by an abrupt gas uptake (Figure 1).

The relatively high gate opening pressure of 0.33 bar is caused by the energy barrier that must be overcome for paddlewheel reconstruction (Figure 1). At the maximum pressure of 0.97 bar, UAM-1O adsorbs 3.6 mmol g^–1^ of *n-*C_4_H_10_ (17.3 wt %), which corresponds to the composition [Zn_2_(oba)_2_TzTz]·3.5*n-*C_4_H_10_. During desorption, UAM-1O releases about 1.1 mmol g^–1^ of *n-*C_4_H_10_, maintaining 2.5 mmol g^–1^ (11.79 wt %; [Zn_2_(oba)_2_TzTz]·2.5*n-*C_4_H_10_) of gas in the pores even at a pressure of as low as 0.002 bar (Figure 1). Corresponding *in situ* PXRDs confirm that UAM-1O exists in two states. In dynamic vacuum, the PXRD indicates only the UAM-1O­(cp). Once the pressure exceeds 0.33 bar, the framework opens, accompanied by the appearance of reflections from the op phase and the gradual disappearance of reflections from the cp phase. At the lowest measured point of the desorption isotherm (*p* = 0.002 bar), reflections from the cp phase are not observed; instead only reflections from the op phase are present, providing additional evidence for the possible retention of *n-*C_4_H_10_ in the pores.

The previously reported mechanism of the UAM-1S transition, cp → op, upon CO_2_ physisorption was determined to be continuous breathing, involving multiple intermediate phases^17^ (Figure S1). This behavior is caused by the preservation^21^ of the metal cluster during desolvation, enabled by higher deformability of soft sba^2–^ linker compared to more rigid oba^2–^ linker (Figure S2). The UAM-1S adsorbs *n-*butane at low pressures with an uptake of 2.7 mmol g^–1^ (13.7 wt %) at 0.002 bar. The uptake rapidly reaches a plateau of 3.0 mmol g^–1^ (14.8wt %), corresponding to [Zn_2_(sba)_2_TzTz]·3.0 *n-*C_4_H_10_ in good agreement with the GCMC simulations (Figure S5). During desorption, only about 0.37 mmol g^–1^ of *n-*butane is released at a pressure of 0.002 bar at 298 K. Thus, 2.6 *n-*butane molecules remain trapped in the pores ([Zn_2_(sba)_2_TzTz]·2.6*n-*C_4_H_10_).

According to *in situ* PXRD patterns collected at 0.0013 bar, reflections from the open phase appear, along with the partial disappearance of reflections from the cp phase (Figure 1). At 0.125 bar, reflections from the closed phase are no longer observed and, importantly, they do not reappear along the desorption branch. Despite applying a vacuum, negligible desorption of *n-*butane occurs from the pores.

Considering that UAM-1S and UAM-1O retain comparable amounts of *n-*butane in their pores (2.5 mmol per mol and 2.6 mmol per mol, respectively) but exhibit different transition mechanisms, they are ideal candidates for studying the thermal effects associated with *n-*C_4_H_10_ desorption by differential scanning calorimetry (DSC) (Figure 2). Subsequent PXRD analyses prove that only the cp phase is present in both materials, confirming that the op → cp structural transformation during DSC has been complete. Computational studies^21^ indicate that the cp phases of both materials are energetically favorable. Notably, the cp phase of UAM-1O is predicted to be more stable than that of UAM-1S, although this prediction has not been verified experimentally yet (Figure S2). DSC analysis of the UAM-1X materials revealed endothermic desorption associated with the phase transition, with enthalpy values of −47 kJ/mol for UAM-1O and −61 kJ/mol for UAM-1S.

**Figure 2:**
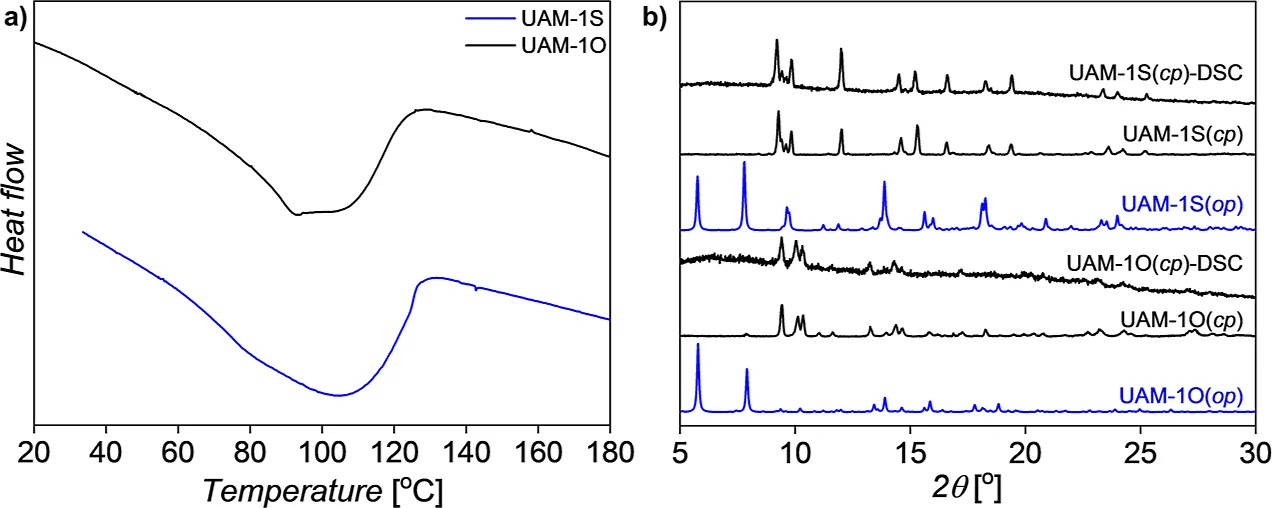
(a) DSC curves for UAM-1O and UAM-1S; heating rate: 5 °C min^–1^. (b) Comparison of the PXRD patterns of materials after DSC measurements, the closed phases and the *n*-C_4_H_10_-filled phase.

The lower heat of desorption of *n-*butane from UAM-1O is related to the higher exothermic effect of the op → cp transformation, which, based on these findings, is estimated to be approximately 14 kJ/mol greater than for UAM-1S. Furthermore, the desorption temperatures of *n-*C_4_H_10_ were determined to be 93 °C for UAM-1O and 106 °C for UAM-1S at atmospheric pressure. For both frameworks, the desorption temperature and the calculated enthalpies suggest values ranging between physisorption and chemisorption processes.^39^

Finally, we performed a series of GCMC simulations using simulated structures of UAM-1S, with varying cell volumes and fractions of free voids by interpolating between the op and cp structures (Figures S5 and S6). These calculations indicate that, in principle, UAM-1S could adsorb gas through a substructure corresponding to approximately 50% of the open*-*pore phase volume. However, no such intermediate phase was observed in the *in situ* data (Figures 1b, 3, and S7), which will be further discussed and rationalized based on combined experimental and theoretical analyses in the following sections.

### Kinetics of *n*-C_4_H_10_ Adsorption

Understanding the adsorption process in flexible MOFs is crucial for their potential applications. However, this requires detailed kinetic investigations to enable the precise determination of the adsorption rate, the dimensional characteristics of the framework’s structural response to guest molecules, and whether the adsorption process occurs locally or cooperatively throughout the entire crystal lattice.^13,14,40,41^ For an in depth analysis, the structural transformation of both frameworks during *n-*butane adsorption was monitored by time-resolved *in situ* PXRD. The Kolmogorov–Johnson–Mehl–Avrami (KJMA)^42^ equation, commonly used to describe the adsorption-driven phase transitions in flexible MOFs,^14,40^ was employed to analyze the data. The KJMA equation is expressed as *a* = 1 – exp­(−*kt* ^*n*^), where *a* represents the fraction of the op phase, *k* is the rate constant, *t* is the time, and *n* corresponds to the dimension of the phase transition.

Due to the high gate opening pressure of UAM-1O­(cp), we used 100 kPa at 273 K, otherwise no structural changes were observed. Under these conditions, the time between gas inlet time (*t*_s_) and adsorption initiation (*t*_0_) is 35 s, and the phase transition end time (*t*_e_), defined as maximum fraction of the open phase, is reached after approximately 600 s. This means that the complete phase transition takes place over roughly 565 s. Interestingly, in the case of UAM-1O during *n-*butane adsorption, kinetic studies indicate that this process can be divided into two stages: a fast and a slow stage. The fast stage lasts from approximately 35 to 230 s, during which the fraction of the open phase reaches a level of 0.7 and then the process slows down significantly, achieving an open*-*phase mole fraction close to 1 that requires an additional ∼500 s (Figures 3 and S7).

This effect can be attributed to the additional phase transition, as evidenced by the peak shift observed in *in situ* PXRD and considerably higher total *n*-butane uptake (0.5 mmol) in UAM-1O as compared to UAM-1S (Figure 1b). The rearrangement of UAM-1O together with the densely packed bulky *n-*C_4_H_10_ within it requires additional time for mutual reorganization and further adsorption.

**Figure 3:**
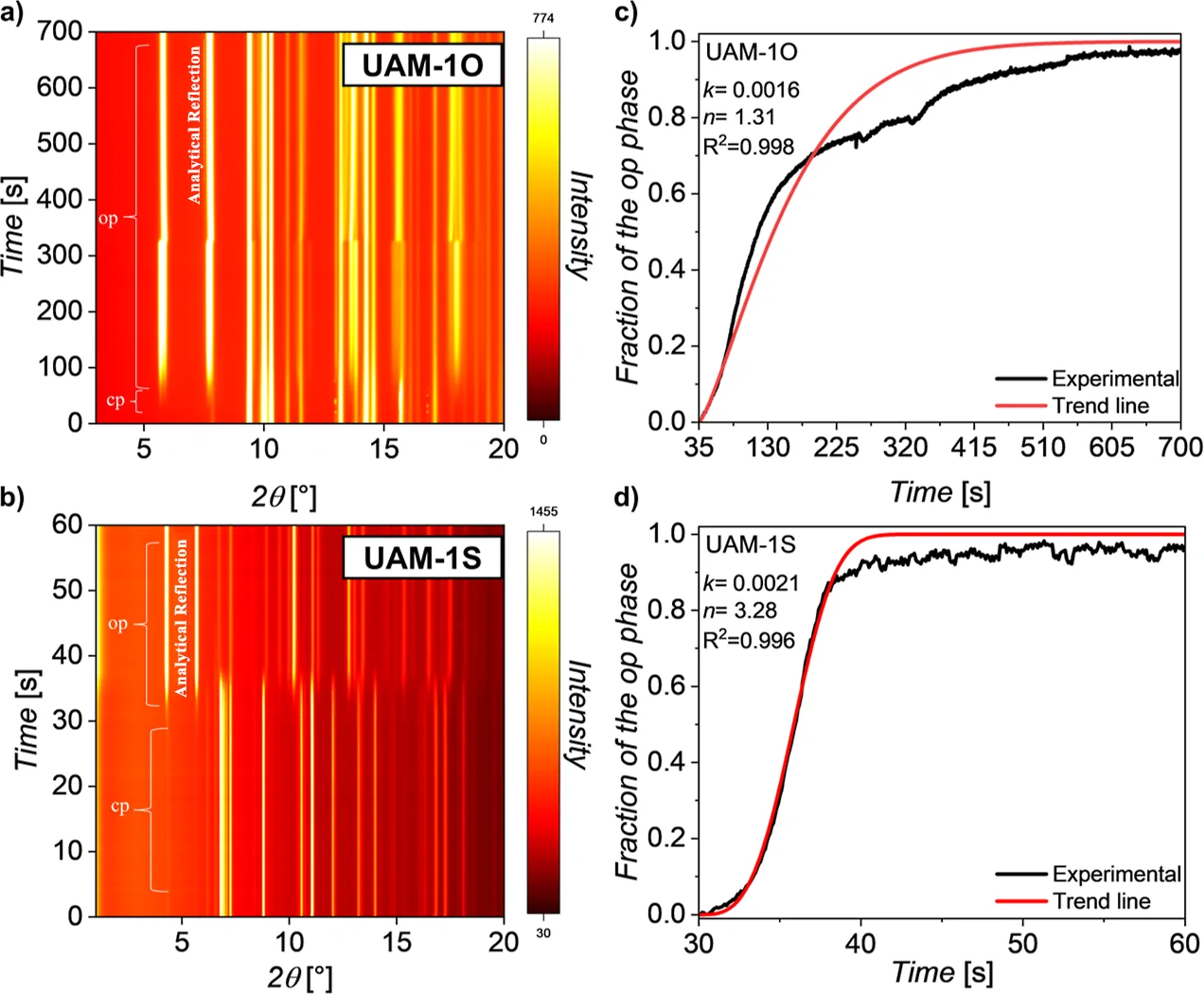
Color maps of in situ PXRD patterns collected upon *n*-C_4_H_10_ adsorption on the (a) UAM-1O at 273 K and 100 kPa and on the (b) UAM-1S at 273 K and 60 kPa. Fractions of the op pore phases for (c) UAM-1O and (d) UAM-1S calculated from *in situ* time-dependent PXRD measurements.

The UAM-1S phase transition kinetics studies of *n-*butane adsorption were carried out at a pressure of 60 kPa. The time between gas inlet (*t*_s_) and adsorption initiation (*t*_0_) is 30 s and the phase transition end time (*t*_e_) is reached after approximately 58.4 s, indicating that the complete phase transition takes place in 28.4 s. For UAM-1S, the *n* factor was determined to be 3.28 and the rate constant *k* was 0.0021 [1/*s* ^3.28^]. Comparing these data to the kinetic data collected for CO_2_ at 60 kPa,^21^ one can see a notable increase in the *n* factor for butane,^21^ as it is only 1.43 for CO_2_. The elevated *n* coefficient indicates that the structural transformation associated with *n*-butane adsorption occurs rapidly and homogeneously throughout the entire crystal lattice and is not preceded by any intermediate state, as was found for CO_2_. Furthermore, we collected Raman spectra during the adsorption process (Figure S9).

### Insights into *n*-Hydrocarbon–MOF Interactions

The determination of the precise position of different hydrocarbons, *n*-butane, *n*-pentane, and *n*-hexane, within UAM-1X is crucial for understanding the mechanism and energy landscape of adsorption. Due to the destructive nature of the activation process (Figure S10), the UAM-1S closed phases could not be used for SC-XRD studies or any manipulations, while its crystals are highly difficult to handle. Therefore, the more manipulation*-*resistant UAM-1O­(op) crystals were used. The UAM-1O­(op)_*n*hydrocarbon_ structures crystallize in the orthorhombic *Pbcn* space group (Tables S1 and S2). These frameworks feature two types of pores that are potentially accessible to guest molecules: small and large.

In the *n-*butane-loaded structure, the position of 1.75 *n-*C_4_H_10_ molecules could be clearly identified in the asymmetric unit (ASU). The remaining disordered guest molecules^43^ corresponds to about 1.47 *n-*butane. Consequently, the total number of *n-*butane molecules per ASU is estimated to be three, giving the composition [Zn_2_(oba)_2_TzTz]·3*n-*C_4_H_10_, which corresponds to the last point on the desorption branch (Figure 1). Crystal structure analysis of the UAM-1O­(op)_*n‑*butane_ reveals a correlation between the pore environment and the conformation of *n-*butane. Within the large pore, *n-*butane adopts the energetically most favorable antiperiplanar conformation (Figures 4 and S11). In contrast, within the small pore, *n-*butane is found in the gauche conformation, which is approximately 3.4 kJ/mol less favorable than the antiperiplanar form. The presence of *n-*C_4_H_10_ in the UAM-1O­(op)_*n‑*butane_ crystals was confirmed after 10 days using TG-MS (Figure S12).

**Figure 4:**
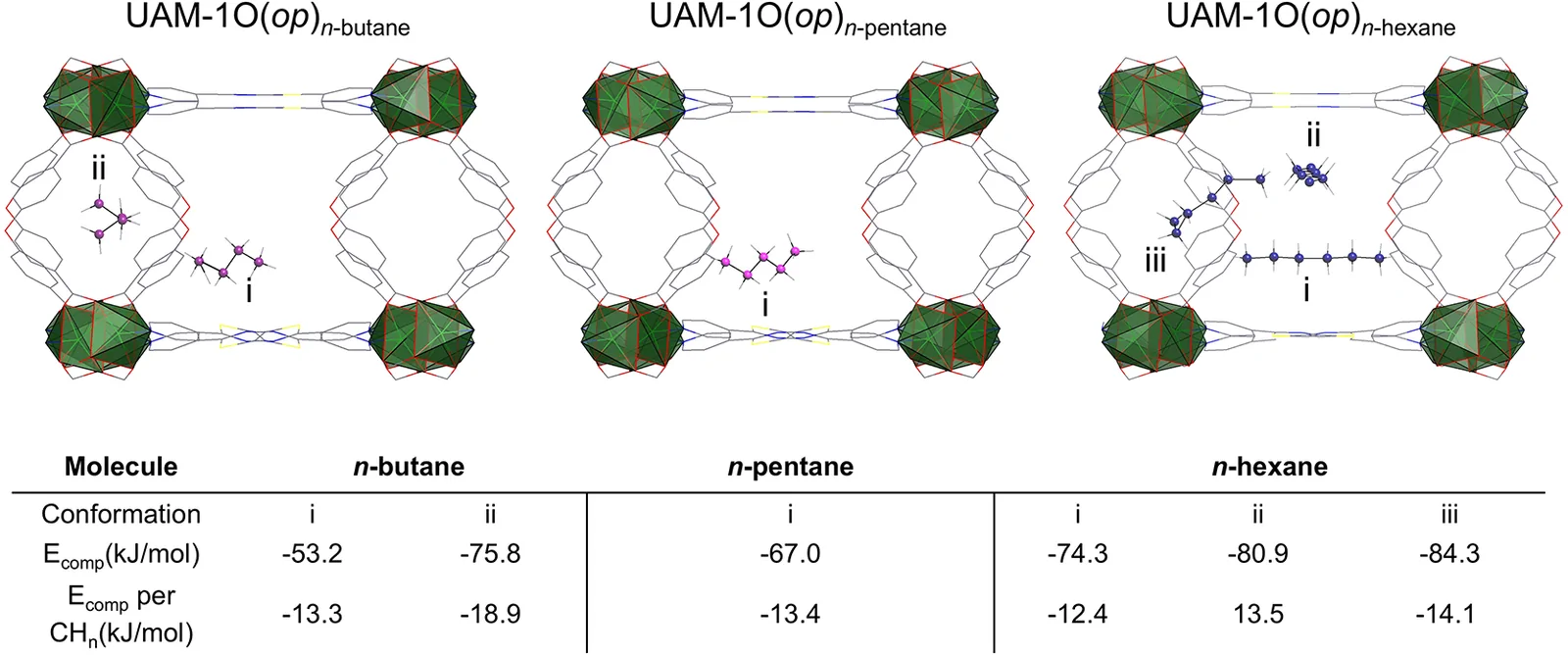
Crystal structures of hydrocarbon-loaded UAM-1O (upper row; C—gray, O—red, H—white, N—blue, S—yellow, Zn—green, *n*-butane—purple, *n*-pentane—pink, and *n*-hexane—dark blue). Calculated interaction energies for different experimental positions of hydrocarbons (lower row) indicated on upper row. Detailed illustrations of the positions and interactions of hydrocarbons with UAM-1O (Figures S11, S13–S17) are presented in the ESI.

In the *n-*pentane-loaded crystals, the guest molecules are confined exclusively in the large pores, where *n-*pentane exists only in the *trans*–*trans* conformation. This observation indicates that the *n-*pentane molecule is sterically too large to adopt a conformation compatible with the small pore (Figure S13). In the crystal structure, 0.667 (2/3) of *n-*pentane molecules are located in the ASU, while solvent masking^44^ in Olex2 revealed an additional 22 e̅ of electron density, corresponding to approximately 0.5 *n-*pentane molecules. Thus, the ASU contains roughly one *n-*pentane molecule in total.

For the *n-*hexane-loaded crystals, the behavior appears intermediate between that of *n-*butane and *n-*pentane. Within the large pore, 2.2 *n-*hexane molecules adopt the all-trans conformation, which is similar to that of *n-*pentane. Simultaneously, a smaller fraction (0.25) adopts a gauche–*trans*–*trans* (GTT) conformation, enabling these molecules to occupy both pore types concurrently (Figure S14). In total, approximately 1.6 of *n-*hexane molecules are present per asymmetric unit, with no residual electron density observed.

Using experimental data, we calculated the binding energies of hydrocarbons depending on their position within UAM-1O (Figure 4, Table S3). Notably, *n-*C_4_H_10_ (i) exhibits markedly stronger binding in the small pore (−75.8 kJ/mol; −18.9 kJ/mol per CH_*n*_) than in the large pore (ii; −53.2 kJ/mol; −13.3 kJ/mol per CH_*n*_), indicating influence of confined environment. For *n-*pentane, only a single crystallographically unique position was identified, with a binding energy of −67.0 kJ/mol, corresponding to approximately −13 kJ/mol per CH_*n*_. On the other hand, *n-*hexane adopts three conformations with binding energies of −74.3, −80.9, and −84.3 kJ/mol, averaging approximately −13 kJ/mol per CH_*n*_.

The origin of the relatively high adsorption energy in the small pore was studied using local energy decomposition (LED). The calculated interaction energy −68.1 kJ/mol, far exceeds typical alkane physisorption of alkanes <50 kJ/mol. LED indicates that London dispersion provided a substantial stabilization of −82.0 kJ/mol comparable to the electrostatic stabilization of −83.8 kJ/mol, whereas the nondispersive correlation contribution was much smaller, −6.4 kJ/mol (Table S4).

The spatially extended dispersion interaction density indicates that pore confinement allows *n*-butane to interact simultaneously with multiple pore wall regions, leading to cooperative accumulation of individually weak van der Waals contacts (Figure S15). Thus, the enhanced adsorption energy is consistent with strong, dispersion-driven physisorption, which is further reinforced by the polar pore environment.

To assess whether the small pore preferentially stabilizes *n-*butane, *n-*pentane and *n-*hexane were additionally modeled fully within this cavity (Figures S16 and S17). Compared to *n-*butane, both hydrocarbons exhibit considerably weaker binding in the small pore: −69.3 kJ/mol (−13.9 kJ/mol per CH_*n*_ unit) for *n-*pentane and −56.0 kJ/mol (−9.3 kJ/mol per CH_*n*_ unit) for *n-*hexane. These results demonstrate that the small pore provides a uniquely favorable environment for *n*-butane.

The *n-*butane affinity toward small pores was also computationally investigated for UAM-1S (Table S5; Figure S18). The energies of adsorbate in the small pore are slightly lower compared to those calculated for *n-*butane adsorbed in the small pore of the UAM-1O structure (−16.6 kJ/mol vs −18.9 kJ/mol per carbon atom in UAM-1S and UAM-1O, respectively). However, this value is considerably lower than for *n-*butane in the large pore (−13.3 kJ/mol). This energetical-fitting serves as the driving force for the direct cp → op transition, without any intermediate states during *n-*butane adsorption (Figures 1b and 3). The result of the GCMC calculations performed for artificially generated intermediate phases of UAM-1S suggest the possibility of intermediate states involving adsorption in the large pore (Figure S5). Based on all these observations, we propose that in the first step *n-*butane diffuses into the small pores. Considering its fitting to the framework, the materials are expected to be fully opened, excluding any intermediate phases.

Smaller adsorbates (Figure S19), such as ethane and propane, exhibit an intermediate adsorption step, whereas isobutane shows an isotherm shape closely resembling that of *n*-butane. In contrast, *n*-pentane is adsorbed directly by UAM-1S without an intermediate step; however, its maximum uptake is lower, corresponding to a stoichiometric composition of [Zn_2_(sba)_2_TzTz]·2*n*-C_5_H_12_ compared to [Zn_2_(sba)_2_TzTz]·3*n*-C_4_H_10_ for *n*-butane.

Unlike the previously reported adsorption mechanisms for CO_2_ at 195 K (Figure S1), no intermediate phase was detected during *n*-butane adsorption in UAM-1S at 273 K. Regardless of whether *n*-butane pressure was changing rapidly (Figure 3) or the pressure was increased gradually under near-equilibrium conditions (Figure 1), PXRD revealed only the cp and op phases.

CO_2_ is a linear molecule with a kinetic diameter of 3.3 Å, whereas *n-*C_4_H_10_, with a kinetic diameter of 4.3 Å, is a flexible zigzag molecule capable of conformation adaptation. The estimated^45^ van der Waals volume of *n*-butane (78 Å^3^) is approximately twice that of CO_2_ (38 Å^3^). Owing to its substantial quadrupole moment, CO_2_ can diffuse into narrow 0D pores, enabling stabilization of intermediate structures.^12,16^

Carbon dioxide adsorption stabilizes multiple intermediate substructures of UAM-1S (Figure S1), most likely through occupation of both pore systems. In contrast, the *n-*C_4_H_10_ molecule is probably preferentially accommodated within the narrow pores of UAM-1S, inducing a direct transformation between the cp and op states (Figures 1 and 3). Therefore, these different adsorption pathways are primarily governed by geometry and host–guest interactions.

### *n*-C_4_H_10_ Capture and Colorimetric Response of UAM-1S

UAM-1S adsorbs *n-*butane at 273 K at very low pressure and is found to retain it within its pores at temperatures exceeding 60 °C (Figure 1). These findings suggest that UAM-1S could be a promising candidate for selective *n-*butane capture below its lower explosive limit of 1.9%. To further evaluate its adsorption performance, single-component adsorption experiments were conducted under ambient conditions using *n-*C_4_H_10_, CO_2_, and H_2_O vapor at 298 K (Figures 5 and S20).

**Figure 5:**
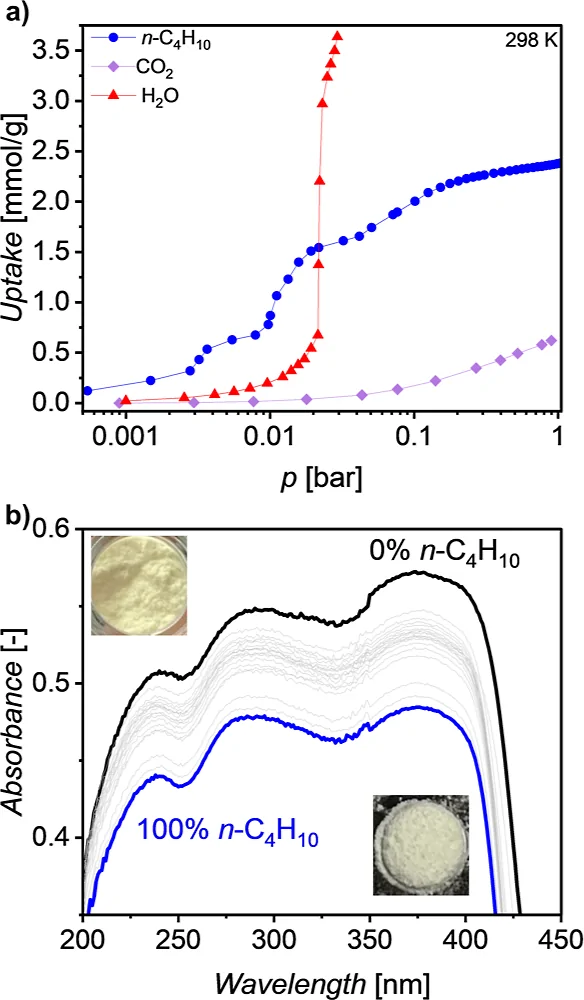
(a) Adsorption isotherms at 298 K. All presented isotherms were recorded on samples that had been stored in the sealed vial for approximately 2 years. (b) *In situ* UV–vis spectra recorded during the flow of *n*-butane at varying *n*-butane gas concentrations at room temperature (black—0%, blue—100%, and gray—intermediate concentrations).

UAM-1S adsorbs *n-*C_4_H_10_ up to 1.5 mmol g^–1^ at a pressure of only 0.016 bar (1.6%), while further increases in pressure leading to an uptake of 2.4 mmol g^–1^ at 0.97 bar. For CO_2_, an uptake of 0.04 mmol g^–1^ is observed at a pressure of 0.018 bar, which is significantly lower than that of *n*-butane. In the case of H_2_O vapor, a noticeable adsorption occurs above 0.02 bar, while at 0.016 bar, only 0.38 mmol g^–1^ is adsorbed. The isotherms clearly indicate a high affinity of UAM-1S for *n*-C_4_H_10_ at room temperature and low pressure.

For an optical analysis, *in situ* UV–vis spectra were recorded during *n-*butane adsorption (Figure 5). The spectrum measured in the absence of *n-*butane exhibits significantly higher absorbance across the UV–visible region, whereas under 100% *n-*butane the absorbance is strongly suppressed, particularly above ∼400 nm. This decrease in absorbance is accompanied by a color change of the MOF from yellow to almost white during *n-*butane adsorption. The effect is particularly pronounced when a pure MOF sample is used for the measurement, as shown in photographs of the MOF before and upon gas exposure (Figure S21).

To assess the origin of the changes observed in the UV–vis spectra, theoretical calculations were performed based on the three experimentally determined structures of UAM-1O (Table S1). In the UAM-1O­(cp), the closest TzTz core–OBA phenyl contact exhibits a centroid–centroid distance of 3.73 Å and an in-plane displacement of 1.47 Å (Figure S22). Upon opening, these parameters increase to 5.54 Å and 4.23 Å for the guest-free open phase and the UAM-1O­(op)_*n*‑butane_, respectively, indicating disruption of the original near face-to-face π-contact.

The calculated Γ-point Kohn–Sham gap increases from 1.09 eV in the cp to 1.77 eV in the empty op and 1.74 eV in the *n*-butane-loaded op phase (Figure S23). Although the absolute values of the calculated gaps should not be directly interpreted as optical transition energies, the calculated trend demonstrates that the framework opening modifies the electronic structure.

Analysis of the projected density of states further indicates that adsorbed *n*-butane does not introduce significant electronic states close to the band edges (Figure S23). Therefore, the optical response is unlikely to originate from guest-to-framework charge-transfer interactions. Instead, *n*-C_4_H_10_ adsorption acts as a structural stimulus that triggers the closed-to-open phase transition, disrupting TzTz–oba π-interactions and consequently altering the electronic structure.

The presence of the shorter TzTz–sba π-contact (3.34 Å; Figure S22) in UAM-1S­(cp), together with the 0.71 Γ-point Kohn–Sham gap in the cp, indicates that the experimentally observed color change in UAM-1S likely originates from the same mechanism. Owing to the shorter π–π contacts, a more pronounced perturbation of the conjugated interactions upon framework opening is observed (Figures S24 and 5) as compared to UAM-1O (Figure S23).

As recyclability and moisture stability are critical factors for adsorbents and sensors, five full isotherms of *n*-butane and three water isotherms were collected on pristine UAM-1S (Figures S24 and S25). After the fifth adsorption cycle, the total uptake decreased by only 3%, while no loss in water uptake was observed. Next, we collected SEM images of the samples after the fifth *n-*C_4_H_10_ cycle (Figure S24). The same samples were subjected to UV–vis and optical imaging analysis before and after exposure to *n*-butane (Figure S24). These experiments confirm that UAM-1S shows an optical response, manifested as a slight wavelength shift and an increase in reflectance, while maintaining its excellent adsorption properties and structural integrity after cycling experiments (Figure S24).

The *n*-butane uptake of UAM-1S is moderate compared with other porous materials such as DUT-8, DUT-49, or MIL-101 (Table S6). Nevertheless, achieving this unique combination of moisture stability, low-pressure *n*-butane capture, cyclability, and a colorimetric response induced by a structural transformation remains challenging in both flexible and rigid MOFs. Under simulated air (*n-*C_4_H_10_/N_2_) flow, dynamic column breakthrough (DCB) experiments demonstrate preferential adsorption of *n-*C_4_H_10_ over N_2_, despite the former’s dilute concentrations. Under dry conditions (98.3/1.7 *v*/*v*, N_2_/*n-*C_4_H_10_, 0% RH), N_2_ elutes rapidly, whereas *n-*C_4_H_10_ reaches saturation at a later stage (Δ*t*_sat_ = 22 min g^–1^), consistent with the preferential affinity of UAM-1S toward *n-*C_4_H_10_ (Figure 6a). To establish an appropriate regeneration protocol, thermogravimetric analysis (TGA) was used to investigate the desorption behavior of adsorbed *n-*C_4_H_10_. Although complete regeneration could be achieved by purging with an inert gas at ambient temperature, the process required approximately 120 min. In contrast, mild heating to only 60 °C reduced the regeneration time to ∼20 min (Figure S27). As this temperature is readily accessible from low-grade waste heat, it was adopted for the subsequent temperature-programmed desorption (TPD) experiments. The corresponding TPD profile exhibits a well-defined *n*-C_4_H_10_ desorption peak (Figure 6b), confirming reversible nature of the adsorption process.

**Figure 6:**
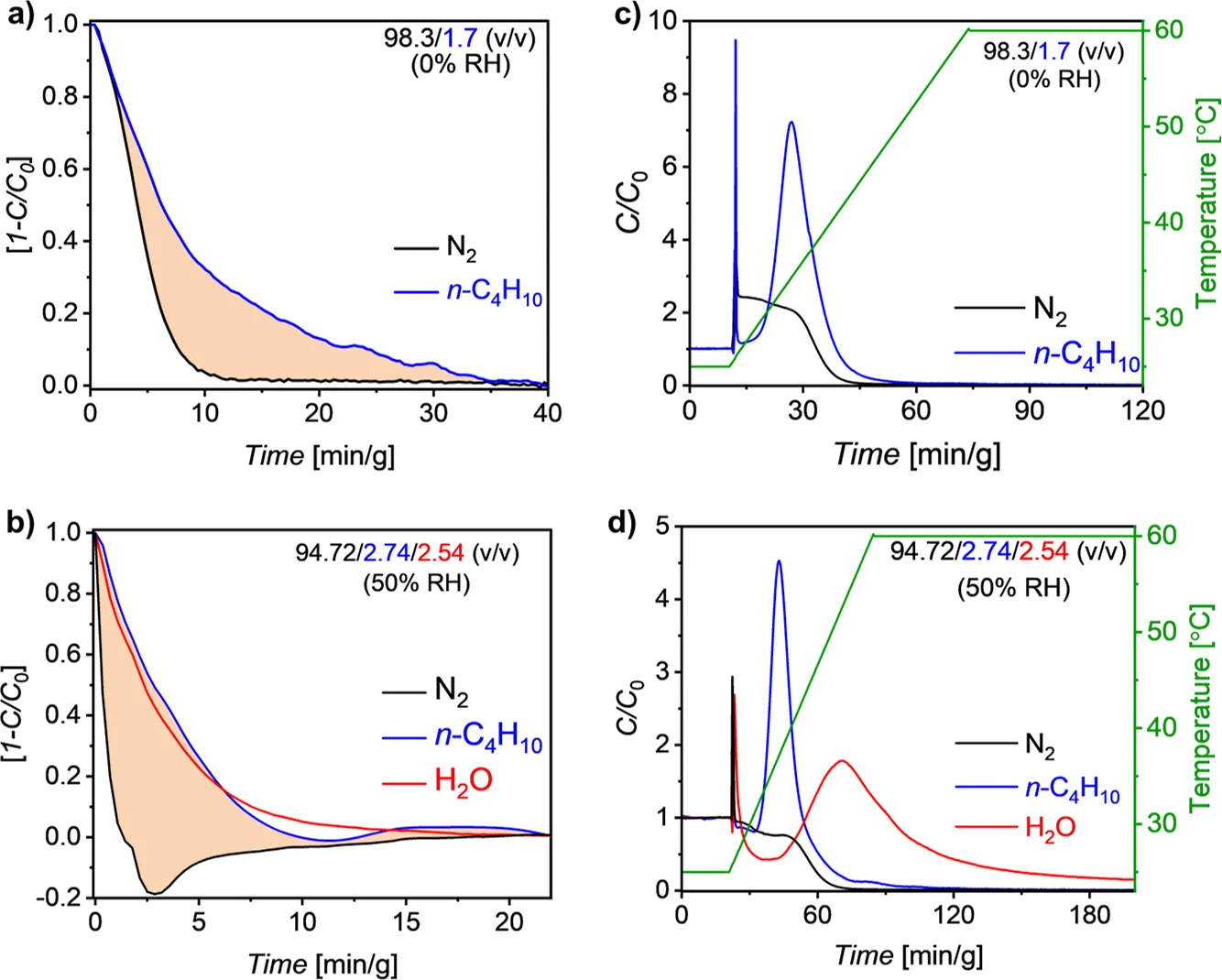
(a) DCB profile for a N_2_/*n*-C_4_H_10_ mixture (98.3/1.7 v/v, 0% RH) showing rapid breakthrough of N_2_ and delayed elution of *n*-C_4_H_10_. The shaded region highlights the separation window between N_2_ and *n*-C_4_H_10_. (b) DCB profile for a humid N_2_/*n*-C_4_H_10_/H_2_O mixture (94.72/2.74/2.54 v/v, 50% RH) revealing competitive adsorption between *n*-C_4_H_10_ and H_2_O while N_2_ breaks through rapidly. (c) TPD profile following adsorption under dry conditions, where *n*-C_4_H_10_ exhibits a pronounced desorption peak upon heating to 60 °C. (d) TPD profile following adsorption under wet conditions showing distinct desorption traces for N_2_, *n*-C_4_H_10_, and H_2_O upon heating.

To evaluate the effect of moisture, DCB experiments were subsequently performed under controlled humid conditions. A dry ternary control experiment employing Ar as the humidity carrier gas (98.3/1.7, N_2_/*n-*C_4_H_10_, v/v, under Ar) produced an essentially identical breakthrough profile to the dry binary experiment (Figure S28), confirming that Ar acts solely as an inert carrier and does not compete for adsorption. Introducing water vapor (95.85/2.92/1.23 and 94.72/2.74/2.54, N_2_/*n*-C_4_H_10_/H_2_O, v/v/v, corresponding to 30 and 50% RH, respectively) resulted in progressively earlier *n*-C_4_H_10_ breakthrough, reducing Δ*t*_sat_ from 22 min g^–1^ to 14.5 min g^–1^ (for 30% RH) and 8.7 min g^–1^ (for 50% RH) (Figures 6b and S29). Quantitative analysis of DCB profiles further revealed a reduction in working capacity with increasing humidity (Figure S29). Despite this decrease, UAM-1S retained ∼65% of its dry working capacity even at 50% RH, highlighting its ability to capture dilute *n*-C_4_H_10_ under humid conditions. The corresponding TPD profiles (Figures 6d and S30) show distinct desorption features for both H_2_O and *n*-C_4_H_10_, confirming coadsorption, while the lower H_2_O desorption intensity at 30% RH is consistent with the reduced water loading. These data are in line with a recent report showing, through computational and experimental studies, that operation under humid conditions is crucial for the environmental application of flexible MOF.^24^

Because enclosed environments may contain additional volatile hydrocarbons, the interference resistance of UAM-1S was evaluated using equimolar ternary mixtures of N_2_/CH_4_/*n*-C_4_H_10_ and N_2_/C_3_H_8_/*n*-C_4_H_10_ (both 1/1/1, v/v/v) (Figure S31). In both mixtures, CH_4_ and C_3_H_8_ exhibited breakthrough substantially earlier than *n*-C_4_H_10_, registering Δ*t* = 115 min g^–1^ (CH_4_) and 90 min g^–1^ (C_3_H_8_). These results demonstrate that the adsorption preference for *n*-C_4_H_10_ is preserved even in the presence of competing light alkanes, highlighting the interference resistance of UAM-1S.

Overall, these breakthrough studies demonstrate that UAM-1S combines selective *n*-C_4_H_10_ capture, regeneration at a mild temperature, tolerance toward humidity, and resistance to interference from coexisting light hydrocarbons. Collectively, these characteristics support its potential for practical *n*-C_4_H_10_ capture from air streams.

## Conclusion

UAM-1O undergoes a first-order structural opening transition at 273 K but remains nonresponsive toward *n*-butane at 298 K within the investigated pressure range. In contrast, UAM-1S exhibits guest-induced switching from the cp to op phase upon *n-*butane adsorption at both temperatures; however, no intermediate phase between the closed and open states is observed, as confirmed by concomitant *in situ* PXRD measurements. This behavior of UAM-1S differs from previously reported continuous breathing transitions upon CO_2_ adsorption at 195 K and is associated with the size matching of *n-*butane to an energetically favorable small-pore environment. The high gas pressure required for cp → op transformation of UAM-1O is attributed to reassemble paddlewheel building units upon guest uptake which is not necessary for UAM-1S. Detailed kinetic investigations based on time-resolved PXRD performed in parallel with the adsorption process demonstrate that even under lower overpressures, UAM-1S exhibits significantly faster uptake kinetics than UAM-1O.

Single-crystal X-ray diffraction analyses of UAM-1O loaded with three homologous *n*-hydrocarbons, combined with DFT-based energy calculations, reveal the preferred adsorption sites and quantify the energetic stabilization responsible for the exceptional fit of *n-*butane within the small pore. Following adsorption, both materials retain the guest molecules under ambient conditions, and elevated temperatures of around 60 °C are required to achieve complete desorption.

Notably, the immediate adsorption capability of UAM-1S in the presence of moisture, accompanied by distinct color changes caused by host-framework structural transformation and confirmed by *in situ* UV–vis spectroscopy as well as optical imaging, underscores the dual functionality of this material as both a sorbent and an indicator for *n*-butane in poorly ventilated environments. These findings open new perspectives in porous materials chemistry, demonstrating that flexible materials can offer advantages over rigid ones by enabling simultaneous dangerous gas capture and sensing applications. The presented concept, exemplified here by a thiazolothiazole MOF, may also be extended to intelligent porous polymers, flexible covalent organic frameworks, and adaptive macrocycles.

## Supplementary Material





## Notes

PXRD patterns, crystal structure drawings, synthetic procedures, crystallographic tables, and additional experimental and theoretical procedures (PDF)
Adsorption Information File (TXT)
