# Supplementary material for: n‑Butane Capture Accompanied by a Colorimetric Response in a Flexible Thiazolothiazole Metal–Organic Framework
Source: J Am Chem Soc. 2026 Sep 7;148(37):39847–56. doi: 10.1021/jacs.6c10210 (PMC13614298; doi:10.1021/jacs.6c10210)
Supplement: Supplementary file 1 [file ja6c10210_si_001.pdf]

Supporting Information

n-Butane Capture Accompanied by a Colorimetric Response in a Flexible Thiazolothiazole Metal–Organic Framework

Szymon K. Sobczak,[a] Asif Raza,[b] Bartosz Mazur,[c] Wola Mishchanka[a], Anastasia Efimova,[d] Volodymyr Bon,[e] Bogdan Kuchta,[c] Michael J. Zaworotko,[b] Stefan Kaskel,[e] Soumya Mukherjee,[b] Kornel Roztocki,[a]* Agnieszka M. Janiak[a]

[a] Faculty of Chemistry, Adam Mickiewicz University, Uniwersytetu Poznańskiego 8, 61-614 Poznań, Poland kornel.roztocki@amu.edu.pl
[b] Department of Chemical Sciences, Bernal Institute and Research Ireland Centre forPharmaceuticals (SSPC), University of Limerick, Limerick V94 T9PX, Ireland
[c] Faculty of Chemistry, Wrocław University of Science and Technology, C. K. Norwida 4/6, Wrocław, Poland
[d] Faculty of Agriculture/Environment/Chemistry Dresden University of Applied Sciences, Friedrich-List-Platz 1 01069 Dresden, Germany
[e] Chair of Inorganic Chemistry, Technische Universität Dresden, Bergstrasse 66, 01062 Dresden, Germany

Table of Contents

Figures **10**

Tables **31**

References **33**

Figures

Figure S1. Mechanistic understanding of CO_2-driven structural transformation in UAM-1X (O – top row; S – bottom row): (a) CO_2 adsorption (full symbols) and desorption (open symbols) at 195 K juxtaposed with (b) corresponding in situ PXRD patterns collected at selected pressure (λ = 1.540599 Å); (c) In situ time-resolved PXRD during the CO_2 adsorption at 195 K. m_{pd}: maximum pore diameter; p_{ws}: pore window size; V_{pt}, theoretical pore volume; and V_{pe}, experimental pore volume for CO_2 adsorption calculated at ~0.99 bar according to the Gurvich's rule. Reproduced from[36] with permission from the Royal Society of Chemistry 10

Figure S2. (a) Relative electronic energy. The values for both materials were calculated independently by subtracting from all values the value of minimum energy for a given cp_1 variant, without Zn–O bond breakage. (b) Energy profile calculated per single Zn-node for the transformation of the cp_1 to cp_2 configuration. Data for UAM-1O ($Zn_2(oba)_2TzTz$) and UAM-1S ($Zn_2(sba)_2TzTz$) were calculated using solid-state NEB, while data for cluster ($Zn_2O_4N_2$) was calculated using NEB. NEB = nudged elastic band methodology. (c) Schematic representation of the considered transition mechanism. The initial structure cp_1 is presented on the left with two oxygens labeled as a and b, in the middle, the transition path with breaking both Zn–O(b) bond and the formation of new Zn–O(a) bond, and on the right are the resulting structures with different resulting nonbonded oxygens. Please note that cp_1 denotes the contracted phase of UAM-1S or UAM-1O, where the coordination number of zinc and the paddle-wheel are preserved, while cp_2 refers to contracted phases with a decreed zinc coordination number to 4 and a disassembled paddle-wheel. All calculations, except for cluster, were done for unit cell containing 16 Zn atoms. Copyright 2025, American Chemical Society.[37] 11

Figure S3. Comparison of experimental and theoretical n-butane isotherms for UAM-1O at two different temperatures. Full circle – theoretical data. Full squares -experimental data. Theoretical data were calculated for open pore phase. 12

Figure S4. Comparison of experimental and theoretical n-butane isotherms for UAM-1S at two different temperatures. Full circle – theoretical data. Full squares -experimental data. Theoretical data were calculated for open pore phase. 12

Figure S5. GCMC-n-butane adsorption isotherms (298 K) for various intermediate structures of UAM-1S. These structures were obtained through computational optimization performed for different pore volumes according to literature procedure.[37] 13

Figure S6. Relation between the theoretical maximum n-butane uptake at 1 bar (298 K) and the cell volume of artificially generated intermediate UAM-1S structures. 13
Figure S7. Colored PXRD maps in full 2Θ range (λ = 0.6199 Å)..................... 14
Figure S8. Selected PXRD patterns of UAM-1O from time-resolved PXRD experiment (λ = 0.6199 Å) showing reflection separation and merging of reflections at 5.69 I 5.85 2θ/º during the adsorption of n-butane over time (the time window of 325–329 s was selected)..................... 14
Figure S9. In situ Raman spectra recorded during n-butane adsorption: range 1325–1625 cm^{-1} (carboxylates region; left); full Raman spectroscopy range (right). The closed-pore phase (initial phase) is marked in blue, the open-pore phase in red, and the intermediate phases in gray. 15
Figure S10. UAM-1X (X: O or S) microscopic images of crystals (with scale bar) after synthesis (left) and after activation (right; DCM exchange; 80 ºC and vacuum), captured under polarized light..................... 15
Figure S11 Positions and lengths of the contacts of n-butane molecules with UAM-1O across the large pore (left) and across the small pore (right) (Gray-C, Blue-N, Red-O, Yellow- S, Green- Zn, White- H)..................... 16
Figure S12. TG-MS analysis of UAM-1O$_{(n\text{-}butane)}$...................... 16
Figure S13 Position and length of the contacts of the n-pentane molecule with UAM-1O in the large pore (Gray-C, Blue-N, Red-O, Yellow- S, Green- Zn, White- H)). 17
Figure S14. Positions and lengths of the contacts of n-hexane molecules with UAM-1O in the large pore (left) and the small pore (right) (Gray-C, Blue-N, Red-O, Yellow- S, Green- Zn, White- H). 18
Figure S15. n-butane interactions with UAM-1O small pore..................... 18
Figure S16. Crystal structure of UAM-1O: with pentane (i – n-pentane identified experimentally; ii – n-pentane generated computationally for calculations (Gray-C, Blue-N, Red-O, Yellow- S, Light Blue- Zn; or clarity, hydrogen atoms were removed from the framework structure). 19
Figure S17. Crystal structure of UAM-1O: with hexane (i–iii – n-hexane identified experimentally; iv – n-hexane generated computationally for calculations) (Gray-C, Blue-N, Red-O, Yellow- S, Light Blue- Zn; white-H)..... 20
Figure S18. n-butane positions in UAM-1S in x-axis view (a), and y-axis view (b). (Gray-C, Blue-N, Red-O, Yellow- S, Light Blue- Zn; white-H) 20
Figure S19. Hydrocarbons adsorption isotherms at 298 K..................... 21
Figure S20. CO_2, H_2O and n-butane adsorption isotherms at 298 K..................... 22
Figure S21. Full in situ IV-VIs range during n-butane adsorption (UAM-1S + BaSO4) (left). In situ UV-vis measured for pure **UAM-1S** with before and after images. 22
Figure S22.. (a) Minimum periodic centroid-centroid distance between a TzTz core and the closest OBA phenyl ring. (b) In-plane displacement of the same contacts, measured relative to the TzTz core plane. (c) Γ-point Kohn-Sham gaps. (d) Representation of the centroids in the crystal structures of UAM-1O and UAM-1S (hydrogen atoms omitted for clarity; carbon, gray; oxygen, red; nitrogen, blue; sulfur, yellow; zinc, green). (e) Color change of the UAM-1O(cp) upon adsorption of n-butane at 278 K. 23
Figure S23. Fragment-projected densities of states for the (a) closed, (b) open (empty), and (c) open (with n-butane) geometries of UAM-1O. Fragment-projected densities of states for the (d) closed, (e) open (empty) UAM-1S. Energies are referenced to the valence-band maximum. Gray shading between the vertical lines marks the Γ-point Kohn–Sham gap. Colored curves and areas show projections onto the OBA or SBA linkers, TzTz linkers, Zn atoms, and n-butane, the gray curve/area is the total density of states. Densities are reported per framework formula unit. 24
Figure S24. a) n-Butane adsorption isotherms measured at 298 K over successive adsorption-desorption cycles (left), with a bar chart indicating the maximum n-butane uptake in each successive cycle (right). b) Photos of the measurement cells containing activated UAM-1S and of the same material after dosing it into the n-butane cell c) Reflective UV-Vis UAM-1S collected for fresh activated sample, the activated sample after five n-butane cycles, and the n-butane loaded sample during the first and sixth cycles **d**) SEM image of UAM-1S(*cp)* recorded after five n-butane adsorption-desorption cycles..................... 25
Figure S25. Humidity stability of **UAM-1S** proved by repeatable adsorption and desorption of water vapor at 298K. Data shows that materials are stable in humid conditions. The data were reproduced with the permission of the RSC.[36]..................... 26
Figure S26. Comparison of the powder diffraction patterns of UAM-1O(op)$_{n\text{-}butane}$ and UAM-1S(op)$_{n\text{-}butane}$...... 27
Figure S27. Regeneration study of UAM-1S (through monitoring TGA) over two adsorption–desorption cycles of n-C_4H_{10}. Sample weight (blue, left axis) (ca. 9 mg) and temperature (red, right axis) were recorded simultaneously. Heating to 60 °C under N_2 flow (60 $mLmin^{-1}$) enables complete desorption, requiring approximately 20 min. Conversely, 120 min was needed to fully desorb during the second cycle when no heat was applied. 27

Figure S28. DCB profile for a ternary N_2/n-C_4H_{10}/Ar mixture (98.3/1.7 v/v, balance Ar) at ambient conditions. Ar was used as the humidity carrier (balance) gas in wet experiments, this control experiment was conducted to decouple the effect of Ar. N_2 and Ar breakthrough rapidly, whereas n-C_4H_{10} exhibits a significantly delayed breakthrough, indicating preferential adsorption of n-C_4H_{10}. And confirming Ar is non-adsorbing gas. The shaded region highlights the separation window between N_2 and n-C_4H_{10}. 28
Figure S29. Dynamic column breakthrough (DCB) curves of n-C_4H_{10} under dry and humid conditions. The shaded area (grey) represents the integrated breakthrough area used to calculate the dynamic working capacity. (a) Dry breakthrough experiment using a 98.3/1.7 (v/v) N_2/n-C_4H_{10} feed (0% RH). (b) Breakthrough experiment using a 95.85/2.92/1.23 (v/v/v) N_2/n-C_4H_{10}/H_2O feed (30% RH). (c) Breakthrough experiment using a 94.72/2.74/2.54 (v/v/v) N_2/n-C_4H_{10}/H_2O feed (50% RH). (d) Comparison of the working capacities obtained under dry and humid conditions after normalization to the dry-feed n-C_4H_{10} concentration (1.70 vol%). The normalization is to factor in the slightly varying n-C_4H_{10} concentrations caused by different humidity. Capacity retention (plotted on the Y2 axis) is reported relative to the dry experiment. 29
Figure S30. (a) DCB profile for N_2/n-C_4H_{10}/H_2O mixture (95.85/2.92/1.23 v/v) at 30% RH, showing preferential adsorption of n-C_4H_{10} and measurable uptake of H_2O, while N_2 breaks through rapidly. The shaded region highlights the separation window between N_2 and n-C_4H_{10}. (b) Corresponding TPD profile following adsorption under the same conditions, revealing distinct desorption features for N_2, n-C_4H_{10} and H_2O upon heating to 60 °C, consistent with co-adsorption and competitive site occupation. 29
Figure S31. DCB experiments demonstrating the interference resistance of **UAM-1S** toward common volatile alkanes. (a) N_2/CH_4/n-C_4H_{10} (1/1/1, v/v/v) breakthrough experiment at 298 K. (b) N_2/C_3H_8/n-C_4H_{10} (1/1/1, v/v/v) breakthrough experiment at 298 K. 30

Tables

Table S1. Crystallographic data of UAM-1O(n-butane, UAM-1O(n-pentane), UAM-1O(n-hexane)..... 31
Table S2. Selected structural information on investigated UAM-$1O_{(n\text{-hydrocarbons})}$ and UAM-$1O_{(DMF)}$[36] 31
Table S3. Calculated interaction energies for different positions of hydrocarbons in UAM-1). * were created artificially. Please refer to **Figures S15** and **S16**. 32
Table S4 Components of the LED analysis of the interaction energy of the fragments. Unit of the energy is kJ/mol. 32
Table S5. Calculated interaction energies for different positions of n-butane within UAM-1S. Gas molecules were artificially put in location corresponding to the observed adsorption in both pores of UAM-1O. Please refer to **Figure S17**. 32
Table S6 Comparison of total n-C_4H_{10} uptake for different materials at 1 bar. Adsorption temperatures are given in brackets. * isotherm were recorded on the fresh sample, ... 32

Experimental and computational procedures

Procedure for the formation of UAM-1O(*n*-butane) crystals: After the synthesis of UAM-1O, DMF was decanted from the crystals and DCM was added. After 3 hours, DMC was decanted and fresh DCM was added and the materials were left in DCM overnight. The next day, DCM was decanted and replaced with fresh DCM. Next, repeat this procedure for another 3 days. The crystals were transferred using a pipette to a glass vial, and excess DCM was gently removed with a paper towel. The vial was placed in an autoclave and sealed. A valve with a pressure gauge was connected to the autoclave. The autoclave was placed in ice, and *n*-butane was introduced. After *n*-butane was introduced, the system was warmed to ambient temperature, and the butane pressure in the autoclave was 3 bar. The crystals were left in an autoclave with *n*-butane for 3 days.

Procedure for the formation of UAM-1O(*n*-pentane) and UAM-1O(*n*-hexane) crystals: After the synthesis of UAM-1O, DMF was decanted from the crystals and DCM was added. After 3 hours, DMC was decanted and fresh DCM was added and the materials were left in DCM overnight. The next day, DCM was decanted and replaced with fresh DCM. Next, repeat this procedure for another 3 days. The crystals were transferred using a pipette to a glass vial, and excess DCM was gently removed with a paper towel. The crystals were then treated with the appropriate solvent, and the vial was tightly closed and left overnight. The next day, the solvents were decanted and replaced with fresh ones and left overnight.

X-ray Crystallography: Reflection intensities for UAM-1O(*n*-butane), UAM-1O(*n*-pentane) and UAM-1O(*n*-hexane) were collected on a Bruker D8 Quest diffractometer equipped with a PHOTON III CPAD detector and an Oxford Cryostream 700 cold nitrogen-gas blower. MoKα X-rays (λ = 0.71073 Å) were generated using an Incoatec Microfocus Source (IμS 3.0). Data reduction was carried out using the Bruker Saint software. Absorption and other corrections were made using SADABS-2016]as implemented in the Bruker APEX 5 software package[1]. Crystal structures were solved by direct methods using the SHELXT program[2] and were refined by full matrix least squares on F^2 using the program SHELXL-2018[3] through the graphical interface XSeed[4]. Non-hydrogen atoms were refined with anisotropic displacement parameters, except for those constituting the minor component in the disordered models, for which the occupancy factors were refined as fractions. All C-bound hydrogen atoms were placed at calculated positions and refined using a riding model with their U_{iso} values constrained to 1.5 times the U_{eq} of their pivot atoms for terminal sp3 carbon atoms and 1.2 times for all other carbon atoms.

In all crystal structures, where necessary, the restraints for the 1,2- and 1,3-distances were applied. In some cases OMIT instruction was used to exclude specific reflections affected by a beam stop. When it was not possible to determine the position of hydrocarbons molecules in the crystals investigated, their contributions were subtracted from the diffraction data using the Solvent Mask option implemented in Olex2.[5,6]

The data have been deposited in the Cambridge Crystallographic Data Collection (CCDC) with deposition numbers CCDC 2537888-2537890. These data can be obtained free of charge via www.ccdc.cam.ac.uk/data_request/cif, by emailing data_request@ccdc.cam.ac.uk, or by contacting The Cambridge Crystallographic Data Centre, 12 Union Road, Cambridge CB2.

Time-resolved *In situ* PXRD experiments were conducted at P23 *in situ* diffraction and imaging beamline of PETRA III synchrotron (DESY). Monochromatic irradiation with E = 20.0 keV (λ = 0.619921 Å) was used in all experiments. Reflection intensities were measured using a PILATUS 1M (DECTRIS) detector. The distance between the sample and detector is 45 cm. In a typical experiment, PXRD patterns were collected with 10 Hz rate during the 300 – 3600 s, integration of the 2D diffraction images was done by the pyFA[7] software. The experimental setup consists of volumetric adsorption instrument BELSORP-max (Microtrac MRB), used as a gas handling system, connected to the

customized home-built *in situ* glass capillary adsorption cell. The dosing of *n*-butane to the cell and PXRD synchrotron measurements were synchronized by a TTL-trigger. The temperature of the experimental cell was controlled at 273 K by Oxford Cryostream 700 Series Cryostream Cooler . In the typical experiment, the desired pressure of *n*-butane was built in the standard volume part while the cell was completely evacuated to the vacuum degree of $p \leq 1$ Pa.

All raw data needed to reproduce the data analysis are available at:
https://researchportal.amu.edu.pl/info/researchdata/UAM72ea1f9403d14124b4735b4fffb0bb70/

***In situ* Raman spectra** have been measured simultaneously with PXRD using portable Raman spectrometer Ocean QE (Ocean Insight) equipped with 785nm Spectrum Stabilized Laser Module.

***In situ* powder X-ray diffraction** measurements were carried out at the KMC-2 beamline at the BESSY II electron storage ring operated by the Helmholtz-Zentrum Berlin für Materialien und Energie. The previously developed in situ PXRDcell, based on beryllium adsorption chamber was mounted on the closed cycle helium cryocooler. The cell was connected to the low-pressure port of the BELSORP-max volumetric adsorption instrument and *in situ* PXRD / *n*-butane physisorption experiments were conducted in the fully automated mode using TTL-signals between adsorption and X-ray diffraction units.[8] A complete *n*-butane adsorption–desorption isotherms at 273 K for UAM-1O and UAM-1S were measured on approximately 50 mg of the sample. The corresponding PXRD patterns were recorded *in situ* after equilibration (0.1% of pressure change within 300 s) at selected points of the isotherm

Isothermal adsorption analysis: Isothermal adsorption analysis: *n*-butane adsorption/desorption studies were performed on a BELSORP-max adsorption apparatus (MicrotracBEL Corp.) at 273 K and 298 K. Prior to the sorption measurements the samples were soaked 3-4 times in DCM for 3-4h and degased at 80 °C for 8-14 h.

Differential Scanning Calorimetry: Differential Scanning Calorimetry: The DSC experiments were conducted using a DSC 1/700 (Mettler Toledo) in temperature range from 20 to 200 °C at a heating rate of 5 K/min. The device was regularly calibrated with reference substances including n-octane, gallium, and indium. The transition temperatures derived from the DSC curves are extrapolated onset temperatures. Enthalpy values were determined as integrals of the peak area relative to the baseline over the entire temperature range where the thermal effect of the process is observed.

Thermogravimetric analyzer coupled to a mass spectrometer: The experiments were carried out using a TG-MS apparatus (PYRIS TGA 1, MS CLARUS 680 SQ8) in the temperature range of 20–250 °C. EIC was recorded for up to 10 ions. Following the SC-XRD measurement, the UAM-1O(op)$_{n\text{-butane}}$ sample was stored in a fridge (4 ºC) for 10 days. Subsequently, TGA coupled with mass spectrometry (**Figure S12**) was performed to verify the presence of *n*-butane within the pores. Upon heating to approximately 100 °C, a mass loss of about 9.6 % was observed. The corresponding mass spectrum exhibited characteristic *n*-butane signals, including the molecular ion ($M^+ = 58$, $C_4H_9^+$) and the fragment ion ($M^+ = 43$, $C_3H_7^+$).

***In situ* UV-Vis spectroscopy:** *In situ* UV–Vis measurements during *n*-butane adsorption were performed using a VARIAN Cary 4000 spectrometer. The MOF sample was ground with $BaSO_4$ in a 1:4 ratio and placed in a measurement cell through which gas was introduced. Different *n*-butane concentrations were obtained by adjusting the flow rates of the inert gas (nitrogen) and *n*-butane, resulting in a concentration range from 0 to 100%. The measurement was also repeated for the pure MOF sample.

Diffuse reflectance UV–Vis spectroscopy measurements were performed using an Agilent Technologies Cary 5000 UV–Vis–NIR spectrophotometer. Initially, a baseline was recorded using an empty sample holder. Subsequently, spectra were collected for the activated materials, the fresh sample, and the sample after repeated *n*-butane adsorption cycles. To investigate the effect of *n*-butane

adsorption, the sample was placed in the adsorption apparatus, evacuated, and then gradually exposed to *n*-butane until a pressure of 1 bar was reached at 298 K. The sample was maintained under an *n*-butane atmosphere for approximately 10 min. It was then rapidly transferred to the sample holder, and diffuse reflectance UV–Vis spectra were immediately recorded.

SEM images were taken on a Hitachi SU-70. Crystals were coated in a 5 nm gold film prior to imaging, using a K550 Emitech Sputter Coater, with a coating current of 20 mA to prevent high-energy electron beam damage of the sample.

Dynamic column breakthrough (DCB) and temperature programmed desorption (TPD) experiments: In typical DCB experiments, ≈440 mg of pre-activated **UAM-10** was placed in a quartz tube (8 mm diameter; 8 mm x 6 mm x 400 mm) to form a packed fixed-bed (**Scheme S1**). First, the adsorbent bed was purged under a 20 cm^3 min^{-1} flow of He gas at 333 K for 30 min prior to breakthrough experiment. Upon cooling to room temperature, the gas flow was switched to the desired N_2/*n*-C_4H_{10}/Ar gas mixture compositions. In dry experiments, Ar was flowed at constant temperature bypassing the water bubbler, whereas in wet experiments, the water bubbler was engaged (**Scheme S1**) to flow Ar through, with constant monitoring of the relative humidity (%RH) using a humidity sensor. Binary and ternary (dry and wet) breakthrough experiments were conducted at 298 K. The outlet composition was continuously monitored by a Hiden HPR-20 Quadrupole Mass spectrometer until complete breakthrough was achieved. Post-breakthrough, TPD experiments were conducted using saturated sorbent beds heated at a constant rate from RT to 60 °C under He flow (20 cm^3 min^{-1}). The desorbed gases were monitored continuously. *N.B.* All percentage compositions included in the manuscript and Supporting Information have excluded argon (as the humidity carrier balance gas).

To compare DCB experiments' results, two characteristic time intervals were used. (A) Saturation time difference (Δt_{sat}) is the difference between the saturation times ($C \approx 0.95C_0$) of two components[9]:

$$\Delta t_{sat} = t_{sat,B} - t_{sat,A}$$

Here t_{sat} corresponds to the time at which the outlet concentration reaches the inlet concentration $C \approx 0.95C_0$. In this work, Δt_{sat} was used to compare the delay in complete saturation of *n*-C_4H_{10} relative to N_2 under dry and humid conditions.

(B) Breakthrough time difference (Δt) is defined as the difference between the breakthrough (or elution) times of two components, where the breakthrough time is taken as the point at which the outlet concentration reaches $C/C_0 \approx 0.01$:

$$\Delta t = t_{B,C/C_0=0.01} - t_{A,C/C_0=0.01}$$

Where t represents the characteristic breakthrough time of each component. This parameter describes the separation window between two eluting species.

The working capacity of *n*-C_4H_{10} was obtained from the breakthrough curves by integrating the area between the normalized breakthrough profile as per[10]:

$$q_{meas} = \frac{F_{y_0}}{m}\int_0^{t_{sat}}\left(1 - \frac{C}{C_0}\right)dt$$

- q_{meas} is the measured working capacity (cm^3(STP) g^{-1}),
- F is the total inlet volumetric flow rate (cm^3 min^{-1}),
- y_0 is the inlet mole fraction of *n*-C_4H_{10},
- m is the adsorbent mass (g),
- C/C_0 is the normalized outlet concentration, and
- t_{sat} is the saturation breakthrough time.

To enable direct comparison of the adsorption performance under different humidity conditions, the measured capacities were normalized to the dry-feed *n*-C_4H_{10} concentration using;

$$q_{norm} = q_{meas} \times \frac{y_{dry}}{y_{humid}}$$

- q_{norm} is the normalized working capacity,
- q_{meas} is the measured working capacity
- y_{dry} is the inlet mole fraction of *n*-C_4H_{10} in dry experiment
- y_{humid} is the inlet mole fraction of *n*-C_4H_{10} in humid experiments

The normalized capacity retention was calculated relative to the dry experiment as

$$Capacity\ retention\ (\%) = \frac{q_{norm}}{q_{dry}} \times 100$$

Where q_{dry} is the normalized working capacity obtained under dry conditions.

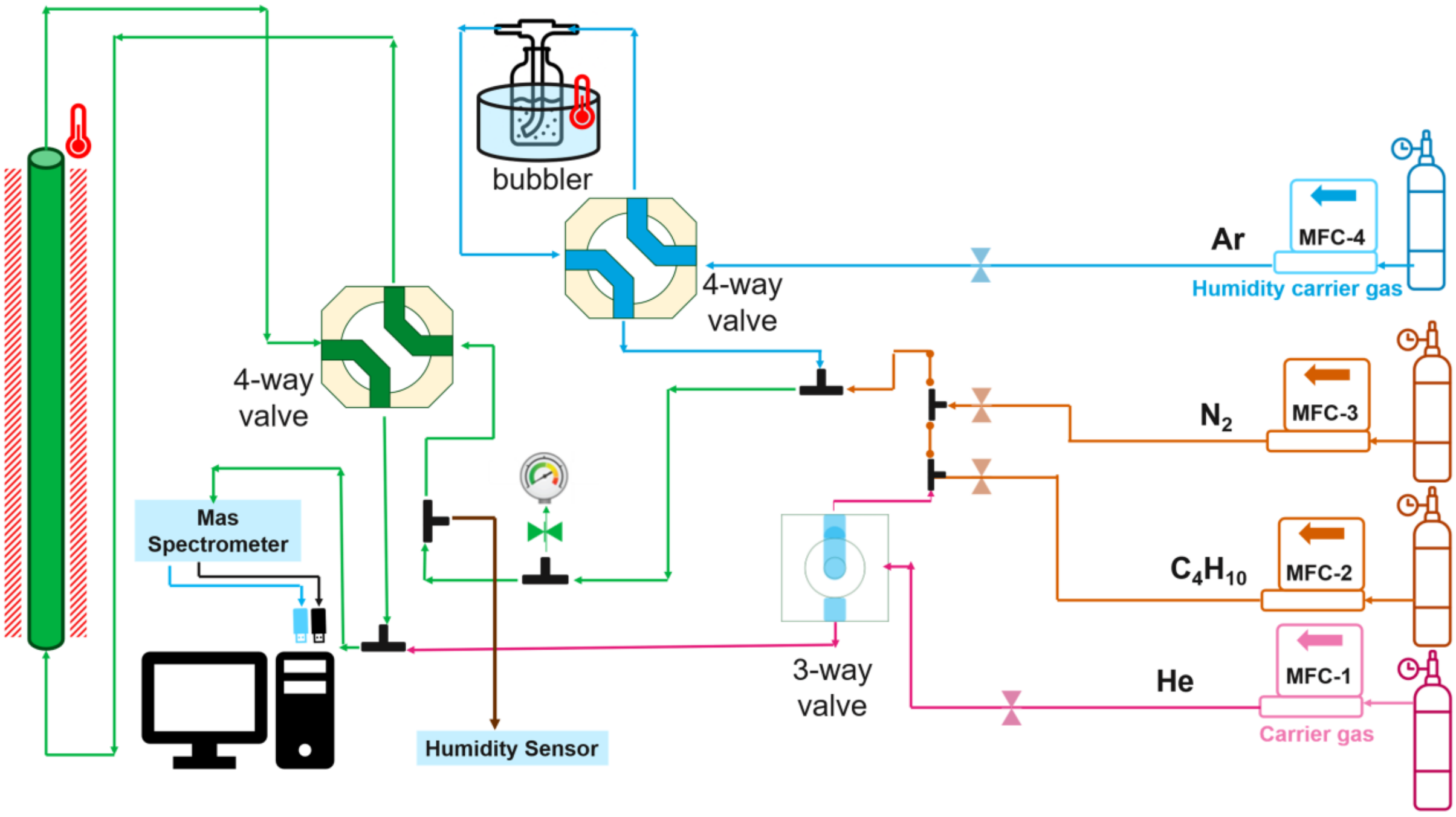

Scheme S1: Schematic of the DCB and TPD experimental setup.

For the interference study with smaller alkanes (CH_4, C_3H_8), a Micromeritics Selective Sorption Analyser (SAA) was used. ~300 mg of pre-activated solid adsorbent was packed in a ¼" (6.35 mm) stainless steel column to form a fixed-bed. Outlet compositions were monitored using an MKS Cirrus 3 mass spectrometer. Prior to recording the adsorption data, the bed was purged with a 40 cm^3min^{-1} flow of pure He for 3 h at 333 K and cooled to RT while maintaining He flow. The flow was then switched to the desired analyte mixture with the temperature maintained at 298 K for the adsorption branch of the experiment. For methane-based gas mixtures, 1$mLmin^{-1}$ flow each of N_2, CH_4, *n*-C_4H_{10} was used while for propane-based gas mixtures, 2 $mLmin^{-1}$ flow each of N_2, C_3H_8, *n*-C_4H_{10} was used. Once saturation was achieved, the flow was switched to He at 20 cm^3min^{-1}, and a temperature ramp rate of 5 K per minute was applied until a column temperature of 333 K was achieved. Temperature and He flow were maintained until concentrations of olefins at outlet were negligible.

Gases, including helium (99.996%), argon (99%), methane (99.99%) and propane (99.9995%) were purchased from BOC Gases Ireland. *n*-butane; 99.95%) was purchased from Air products. Each was used directly without further purification.

DFT Calculation Methodology: Calculations were carried out using periodic density-functional theory (DFT) method as implemented in VASP 6.5.0 package with the PAW method.[11] The cutoff energy for the plane wave basis set was 700 eV with a single gamma k-point. The energy convergence for SCF loop was set to 1e-6 eV. Gaussian smearing of the band occupancies with a smearing width of 0.05 eV was employed. The PBE density functional[11] with the Grimme's D3 scheme[12] and Becke–Johnson (BJ) damping[13] for the empirical dispersion correction was utilized. In the geometry optimization simulations with forced conformations of pentane and hexane in small pore, forces were converged to 0.02 eV/Å and only atom positions were allowed to optimize (with cell parameters kept fixed). Initial structures were obtained from experimentally recorded data. For each hydrocarbon, a single molecule was left in each recorded symmetry unique position. As pentane and hexane adsorption in small pore was not observed experimentally, butane conformation was modified by adding one or two CH_n groups to adsorbed butane, followed by atom positions geometry optimization. The DFT complexation energy was calculated as:

$$\Delta E_{comp} = E_{mof+gas} - E_{mof} - E_{mol}$$

Where $E_{mof+gas}$ is the energy of MOF with adsorbed hydrocarbon, E_{mof} is the energy of MOF at the same conformation but without adsorbed molecule, and E_{mol} is the energy of extracted molecule in the same periodic conditions

Given that the UAM-1S structure had not been experimentally recorded with adsorbed butane, we manually placed gas molecules in locations corresponding to the observed adsorption in both pores of UAM-1O. Two butane molecules were placed within a large pore and two within a small pore (**Figure S17**). Subsequently, the atom positions of both the fluid and the framework were fully optimized. Following the geometry optimization, the single-point energy calculations were performed to calculate the complexation energy at given geometry (**Table S3**). It has been demonstrated that the energies of molecules adsorbed in large pore are equal to the calculated complexation energies of butane adsorbed in large pore of the UAM-1O structure. The energies of molecules adsorbed in the small pore exhibit a slight decrease in value when compared to the energies of butane adsorbed in the small pore of UAM-1O structure (-16.6 kJ/mol vs. -18.9 kJ/mol per carbon atom in UAM-1S and UAM-1O, respectively). This discrepancy may be attributable to the inability to attain the global minimum, a consequence of the exceedingly confined dimensions within the narrow small pore. This finding is further corroborated by a comparison of the energies of conformations *iii* and *iv*, both of which are adsorbed in a small pore. However, a notable trend emerges wherein the energy of complexation in small pores exhibits a higher value compared to molecules adsorbed in large pores.

GCMC Methodology: Computations were performed using the BEM2 cluster[14] at the Wrocław Centre for Networking and Supercomputing (WCSS), with access provided by PL-Grid Infrastructure.[15] Simulations were carried out with the RASPA 2.0.50 software package.[16] The force field parameters were taken from the libraries distributed with RASPA: the UFF force field was used for Zn atoms,[17] and the DREIDING force field for all other framework atoms.[18] The definition of *n*-butane followed the TraPPE[19] force field. Simulation directory structures and input files were generated using Bash scripts while graphs were prepared with Python 3 and matplotlib[20] library.

An example of input file:

```
SimulationType MonteCarlo
NumberOfCycles 50000
NumberOfInitializationCycles 20000
PrintEvery 10000

PrintForcefieldToOutput no
PrintPseudoAtomsToOutput no
PrintMoleculeDefinitionToOutput no

Forcefield ExampleMOFsForceField

Framework 0
FrameworkName uam1s-1
```

```
UnitCells 2 2 2
ExternalTemperature 298.0
ExternalPressure 100000

Component 0 MoleculeName butane
MoleculeDefinition ExampleDefinitions
SwapProbability 5.0
TranslationProbability 3.0
RotationProbability 2.0
ReinsertionProbability 2.0
CreateNumberOfMolecules 0
```

Local energy decomposition (LED) calculations[21–23] were performed with ORCA 6.1.1[24] using the DLPNO-CCSD(T) method[25,26] The def2-TZVP basis set[27] was employed for all atoms together with the corresponding def2-TZVP/C[28] and def2/J auxiliary basis sets[29]. The RIJCOSX approximation[30] VeryTightSCF convergence criteria, and TightPNO thresholds were used throughout.

The representative fragment-based small pore model was extracted from the periodic structure with adsorbed butane. The cluster was truncated as shown on Figure 3, to include all important close contacts for the butane molecule. At the places of truncation, the structure was accordingly saturated with hydrogen or lithium atoms, to preserve neutrality of the molecular fragment. The geometry of added atoms was then optimized with r2SCAN-3c method,[31] with default simulation parameters implemented in ORCA. Next, this neutral, closed-shell cluster was divided into two fragments, with n-butane defined as fragment 1 and the UAM-1O cluster as fragment 2. The LED calculation was carried out at the adsorption-complex geometry. Additional DLPNO-CCSD(T) single-point calculations were performed for the isolated n-butane and UAM-1O fragments, while retaining their geometries from the complex. The resulting energy therefore corresponds to the interaction energy at the fixed complex geometry and does not include a separate geometrical-deformation contribution.

The interaction energy was decomposed according to:

$$\Delta \mathrm{E}_{int.} = \Delta E^{\mathrm{ref.}}_{\mathrm{el.\ prep.}} + E^{\mathrm{ref.}}_{\mathrm{electrost.}} + E^{\mathrm{ref.}}_{\mathrm{exch.}} + \Delta E^{C-CCSD.}_{non-disp.} + E^{C-CCSD.}_{disp.} + \Delta E^{C-(T).}_{int.}$$

The dispersion contribution was obtained as the sum of the strong- and weak-pair dispersion terms. The electronic-preparation, non-dispersive correlation, and perturbative-triples interaction terms were evaluated from the corresponding complex and isolated-fragment calculations following the standard LED procedure. Dispersion-interaction densities[32,33] generated using the DoDIDplot option, exported as Gaussian cube files, and visualized together with the molecular structure/electron-density surface using CrystalMaker 11.7.1[34] [ChimeraX 1.12 software.[35]

Figures

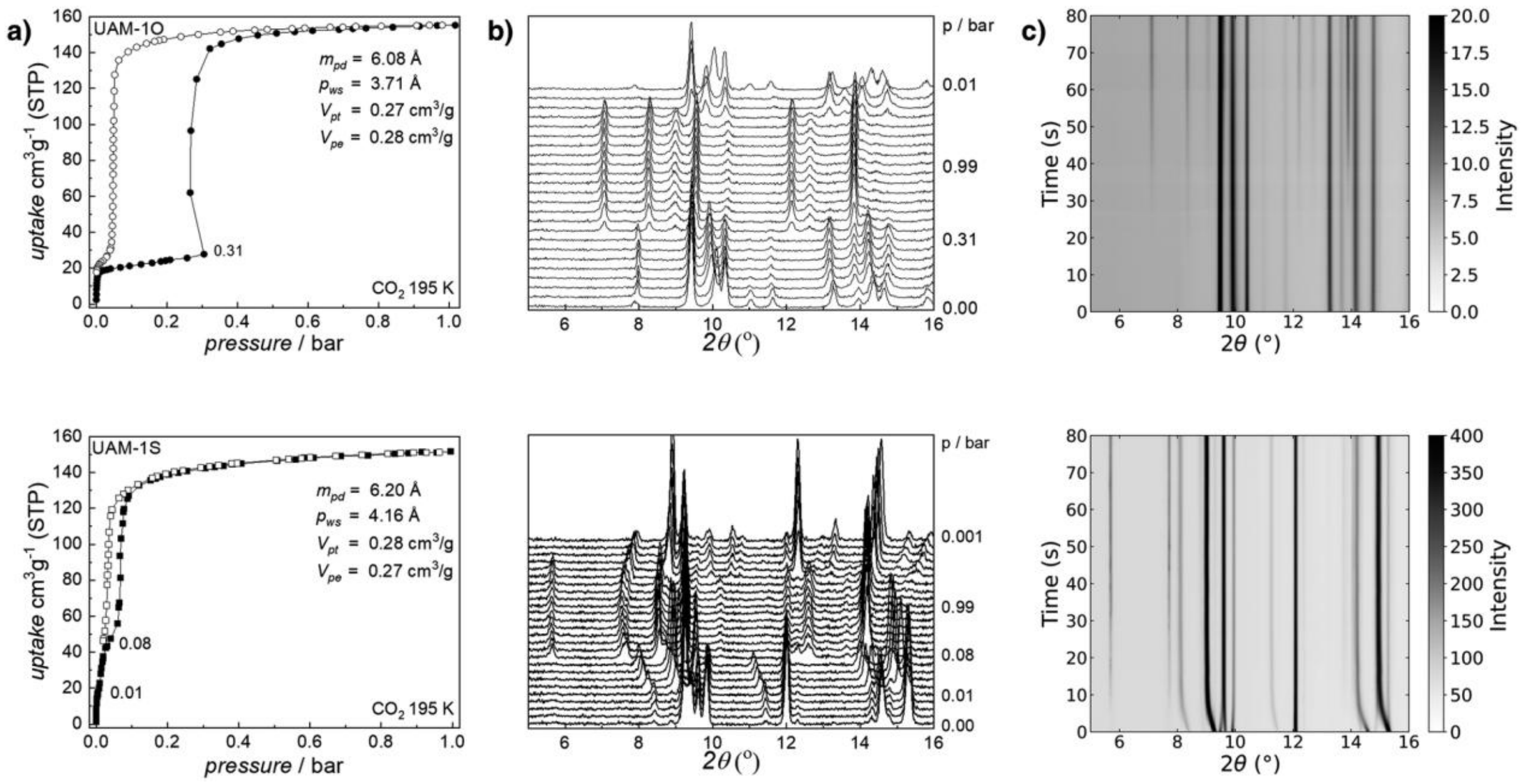

Figure S1. Mechanistic understanding of CO_2-driven structural transformation in UAM-1X (O – top row; S – bottom row): (a) CO_2 adsorption (full symbols) and desorption (open symbols) at 195 K juxtaposed with (b) corresponding *in situ* PXRD patterns collected at selected pressure (λ = 1.540599 Å); (c) *In situ* time-resolved PXRD during the CO_2 adsorption at 195 K. m_{pd}: maximum pore diameter; p_{ws}: pore window size; V_{pt}, theoretical pore volume; and V_{pe}, experimental pore volume for CO_2 adsorption calculated at ~0.99 bar according to the Gurvich's rule. Reproduced from[36] with permission from the Royal Society of Chemistry

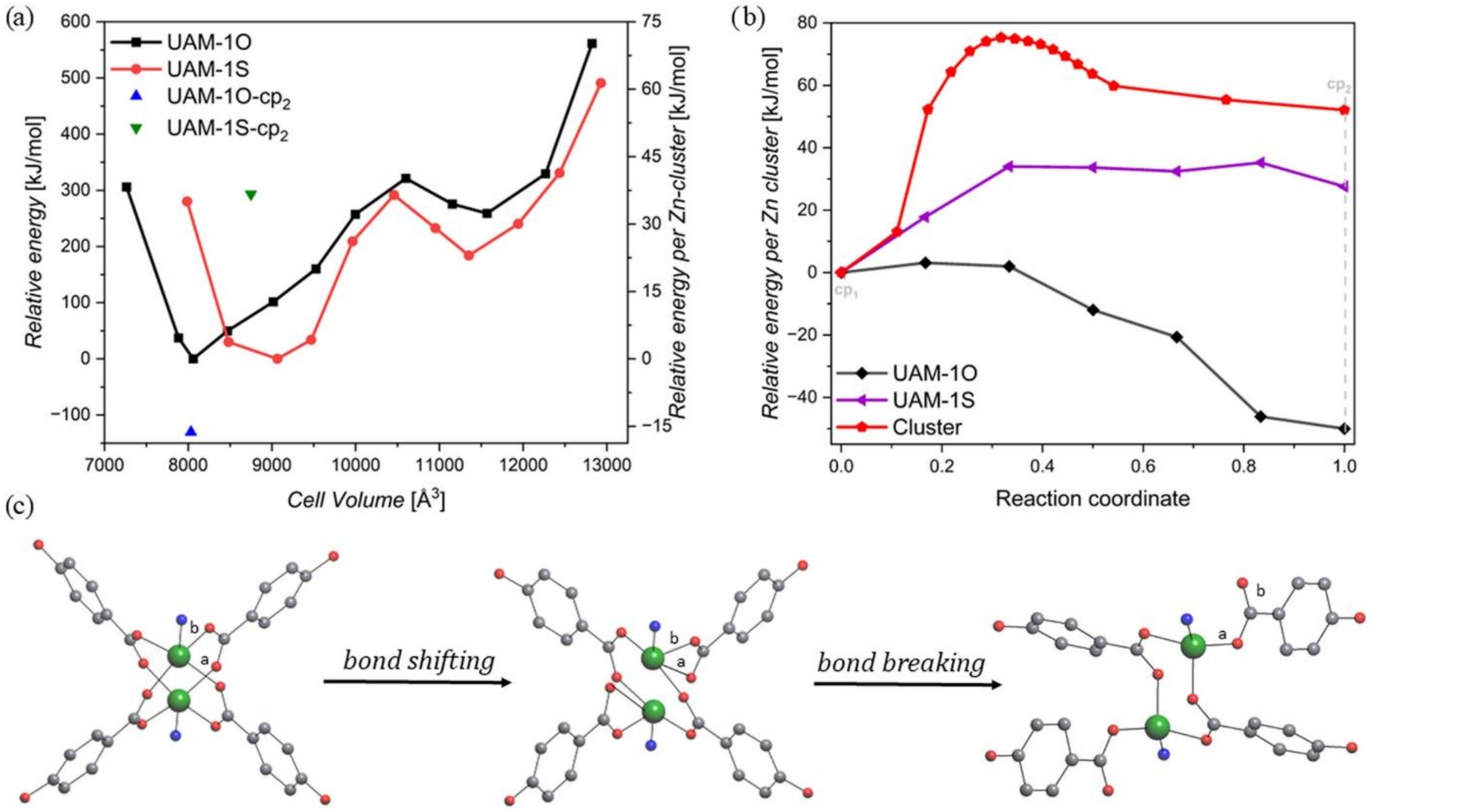

Figure S2. (a) Relative electronic energy. The values for both materials were calculated independently by subtracting from all values the value of minimum energy for a given cp_1 variant, without Zn–O bond breakage. (b) Energy profile calculated per single Z*n*-node for the transformation of the cp_1 to cp_2 configuration. Data for UAM-1O ($Zn_2(oba)_2TzTz$) and UAM-1S ($Zn_2(sba)_2TzTz$) were calculated using solid-state NEB, while data for cluster ($Zn_2O_4N_2$) was calculated using NEB. NEB = nudged elastic band methodology. (c) Schematic representation of the considered transition mechanism. The initial structure cp_1 is presented on the left with two oxygens labeled as a and b, in the middle, the transition path with breaking both Zn–O(b) bond and the formation of new Zn–O(a) bond, and on the right are the resulting structures with different resulting nonbonded oxygens. Please note that cp_1 denotes the contracted phase of UAM-1S or UAM-1O, where the coordination number of zinc and the paddle-wheel are preserved, while cp_2 refers to contracted phases with a decreed zinc coordination number to 4 and a disassembled paddle-wheel. All calculations, except for cluster, were done for unit cell containing 16 Zn atoms. Copyright 2025, American Chemical Society.[37]

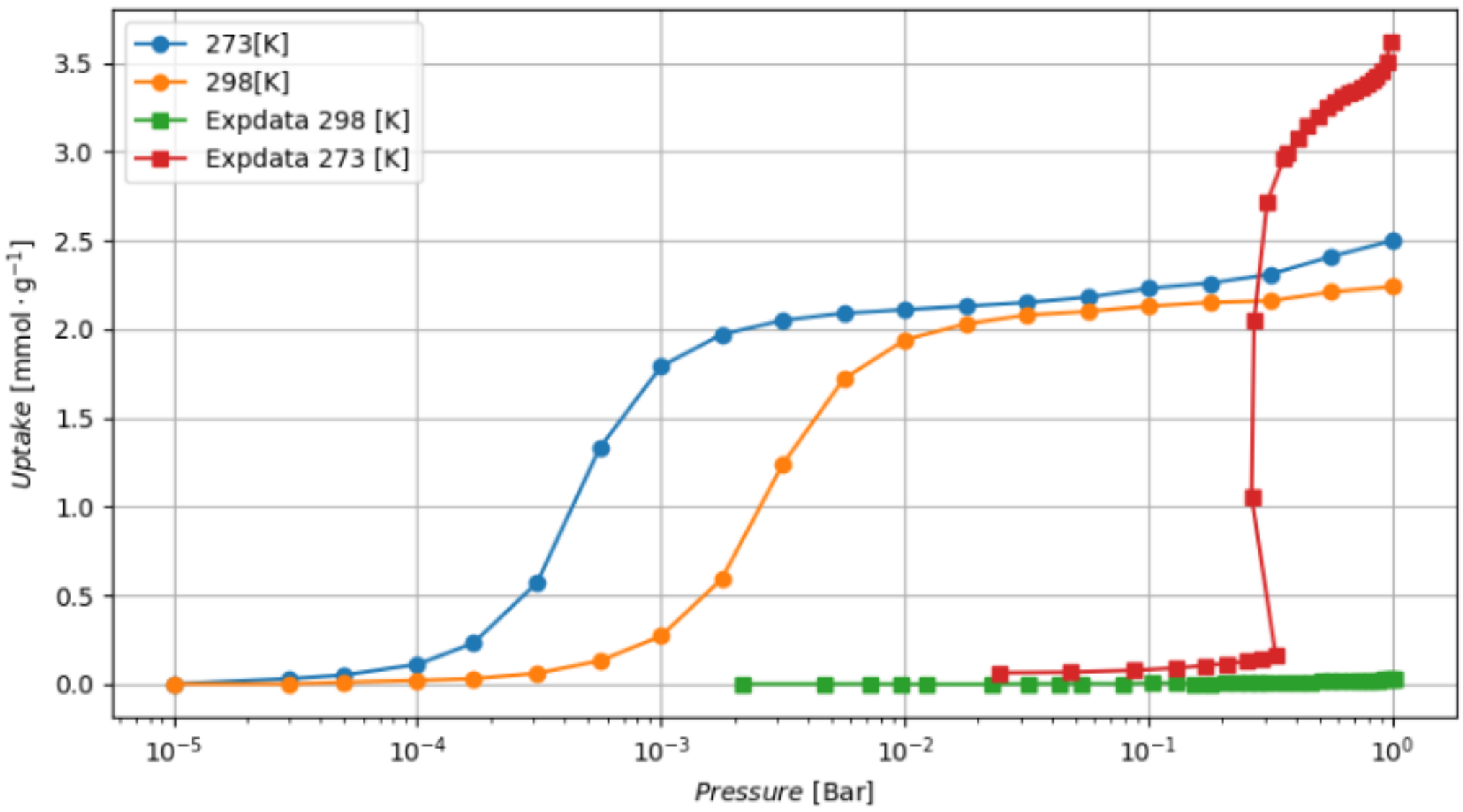

Figure S3. Comparison of experimental and theoretical *n*-butane isotherms for UAM-1O at two different temperatures. Full circle – theoretical data. Full squares -experimental data. Theoretical data were calculated for open pore phase.

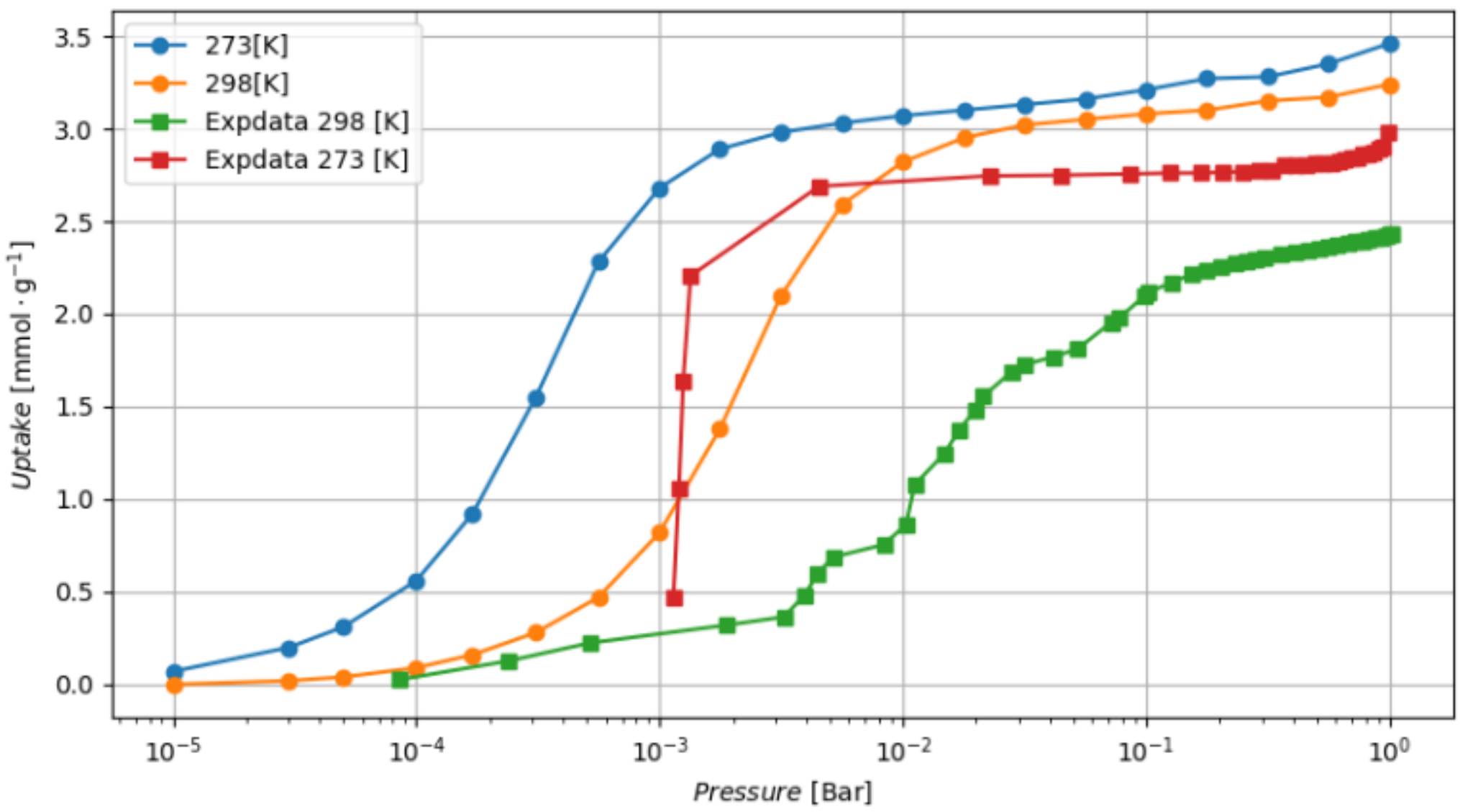

Figure S4. Comparison of experimental and theoretical *n*-butane isotherms for UAM-1S at two different temperatures. Full circle – theoretical data. Full squares -experimental data. Theoretical data were calculated for open pore phase.

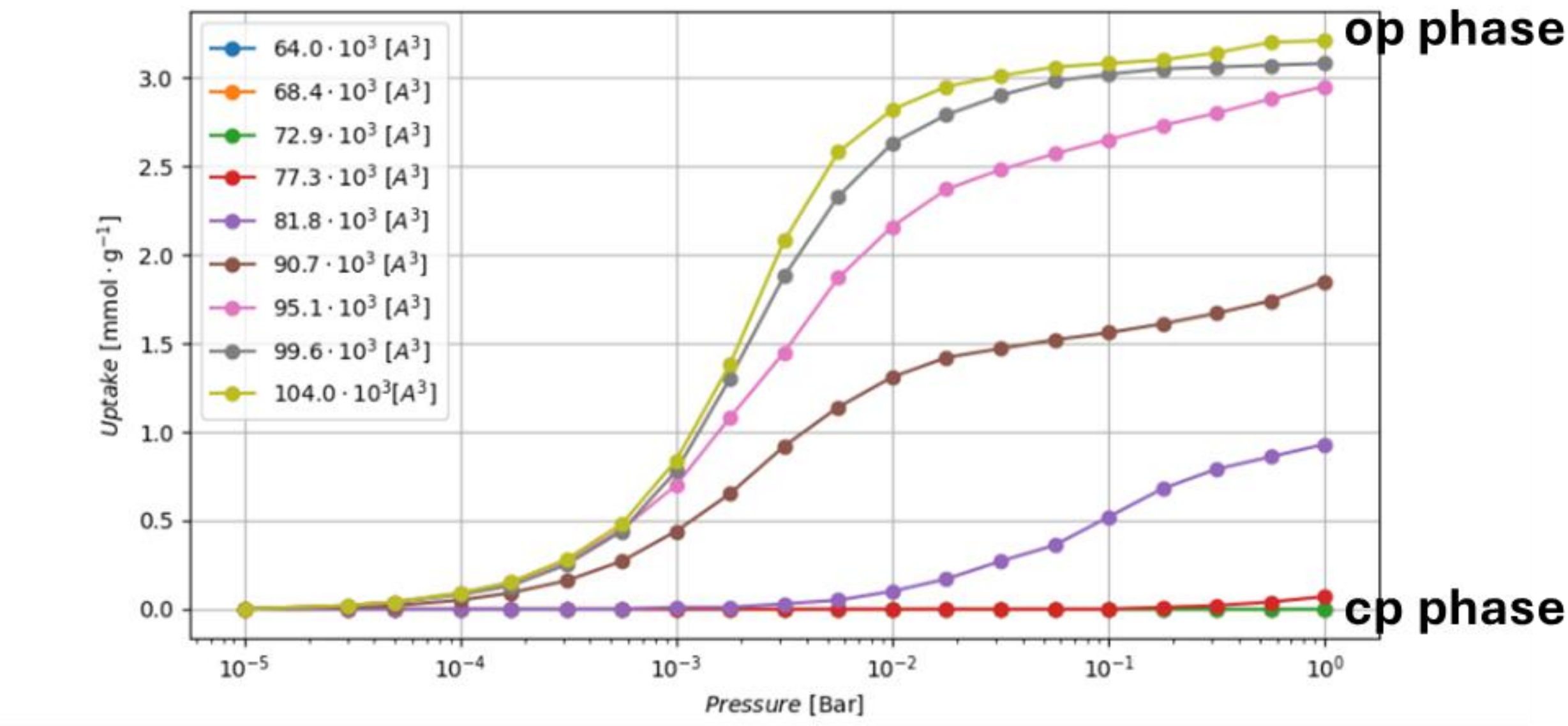

Figure S5. GCMC-*n*-butane adsorption isotherms (298 K) for various intermediate structures of UAM-1S. These structures were obtained through computational optimization performed for different pore volumes according to literature procedure.[37]

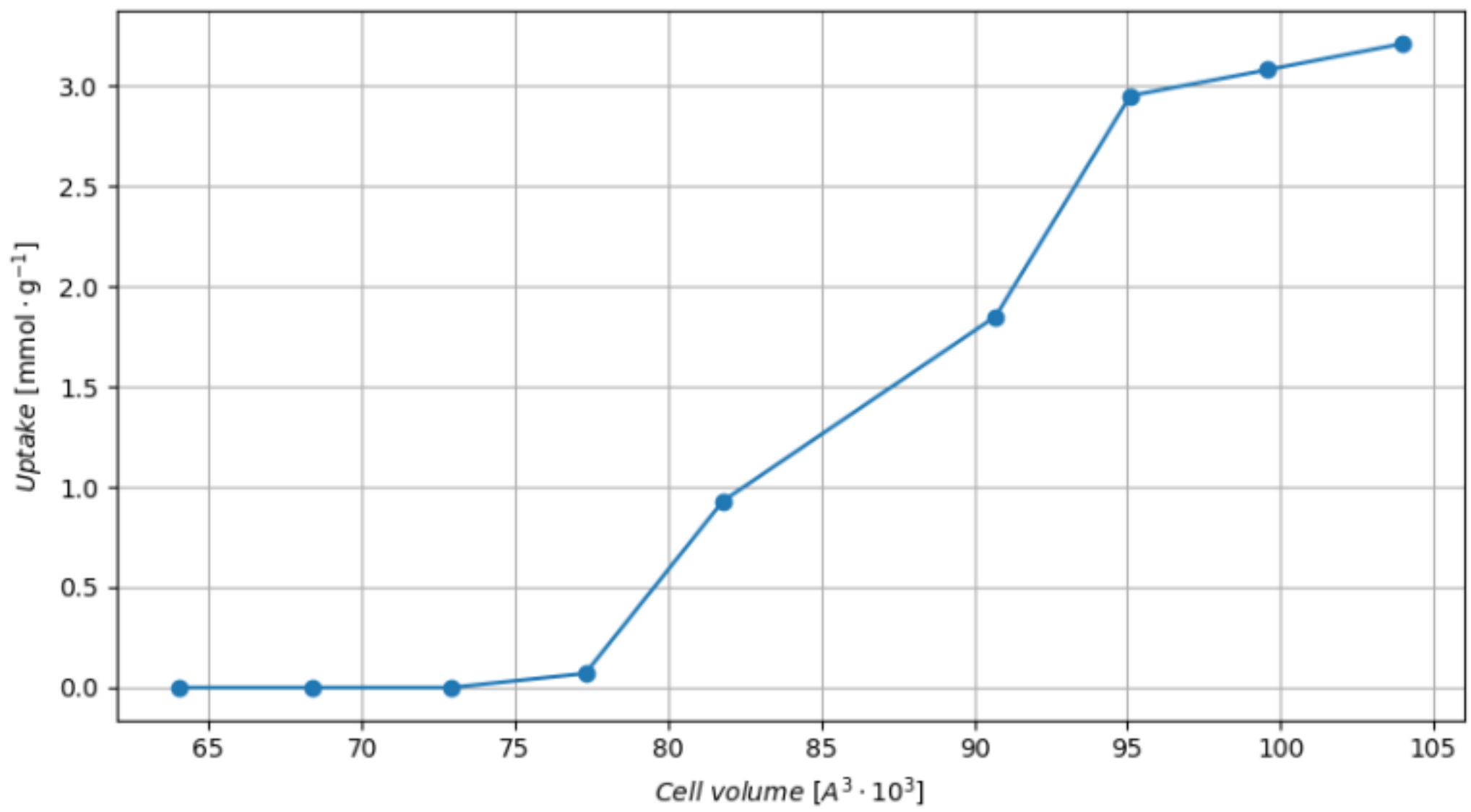

Figure S6. Relation between the theoretical maximum *n*-butane uptake at 1 bar (298 K) and the cell volume of artificially generated intermediate UAM-1S structures.

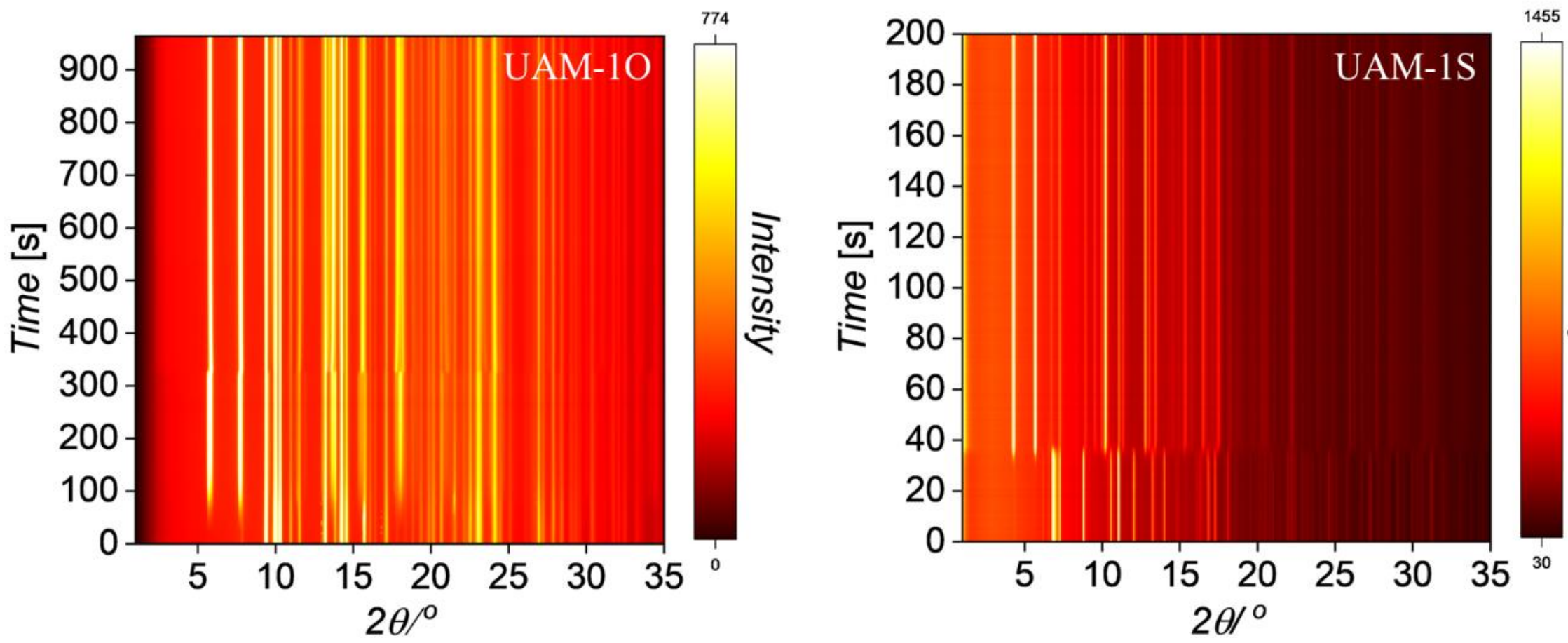

Figure S7. Colored PXRD maps in full 2Θ range (λ = 0.6199 Å).

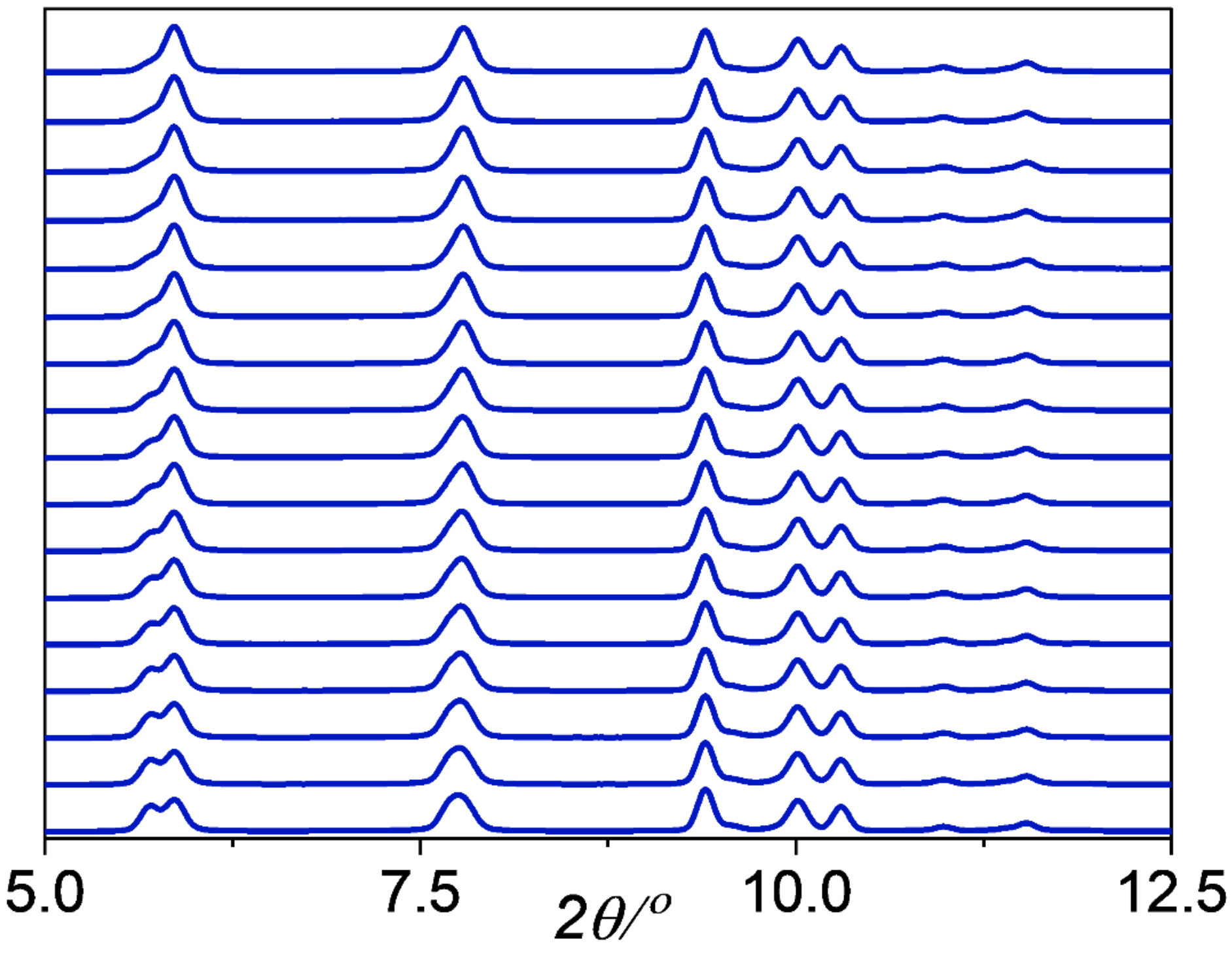

Figure S8. Selected PXRD patterns of UAM-1O from time-resolved PXRD experiment (λ = 0.6199 Å) showing reflection separation and merging of reflections at 5.69 I 5.85 2θ/° during the adsorption of *n*-butane over time (the time window of 325–329 s was selected).

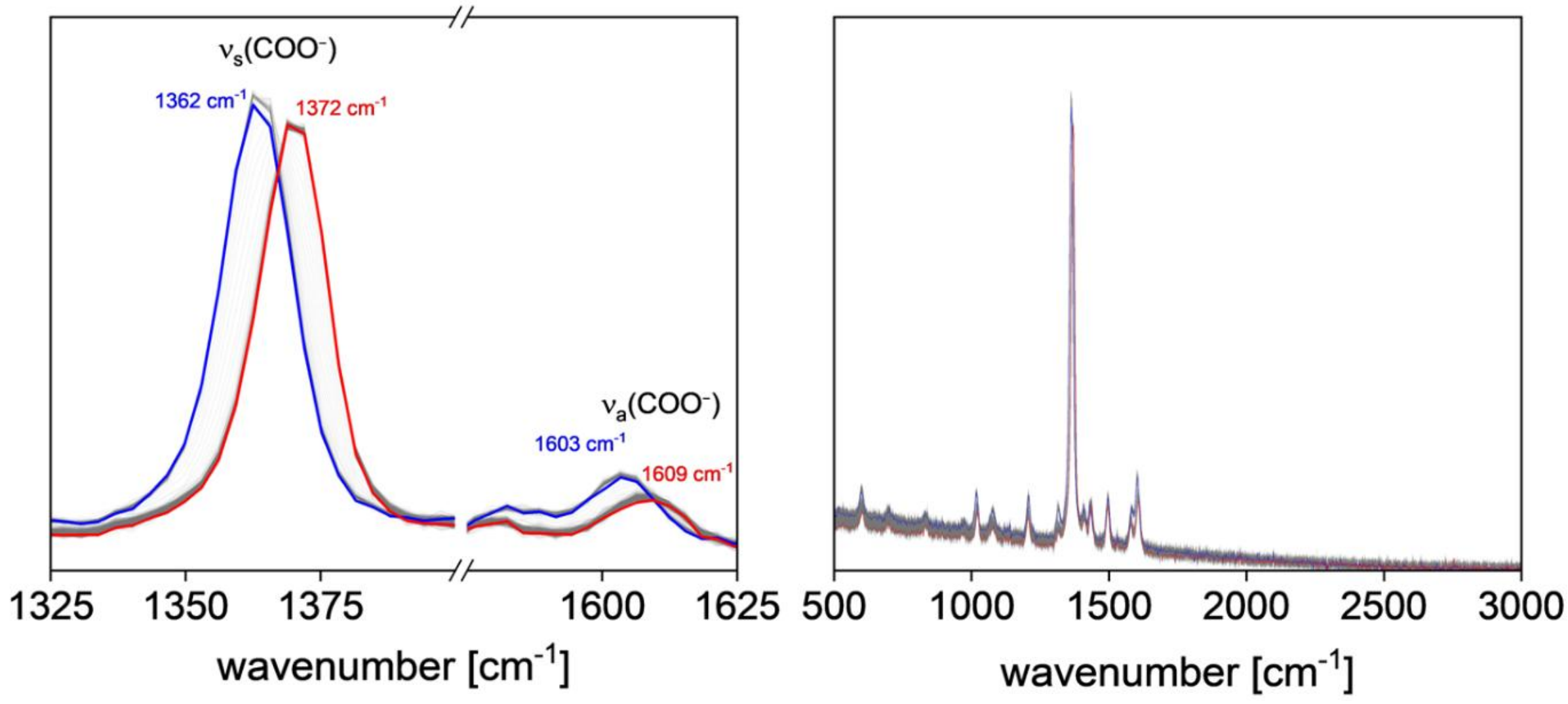

Figure S9. *In situ* Raman spectra recorded during *n*-butane adsorption: range 1325–1625 cm^{-1} (carboxylates region; left); full Raman spectroscopy range (right). The closed-pore phase (initial phase) is marked in blue, the ope*n*-pore phase in red, and the intermediate phases in gray.

Since the chemical bonds remain intact during the adsorption and activation in UAM-1S, no disappearance or formation of new bands is observed in the Raman spectrum; rather, only shifts of the pre-existing bands are detected. This is particularly evident in the shift of the very intense band at 1362 cm^{-1} to 1372 cm^{-1}, originating from the symmetric vibration of the carboxylate group, and of the much weaker band at 1603 cm^{-1} to 1609 cm^{-1}, arising from the asymmetric vibration of the carboxylate group (**Figure S9**).

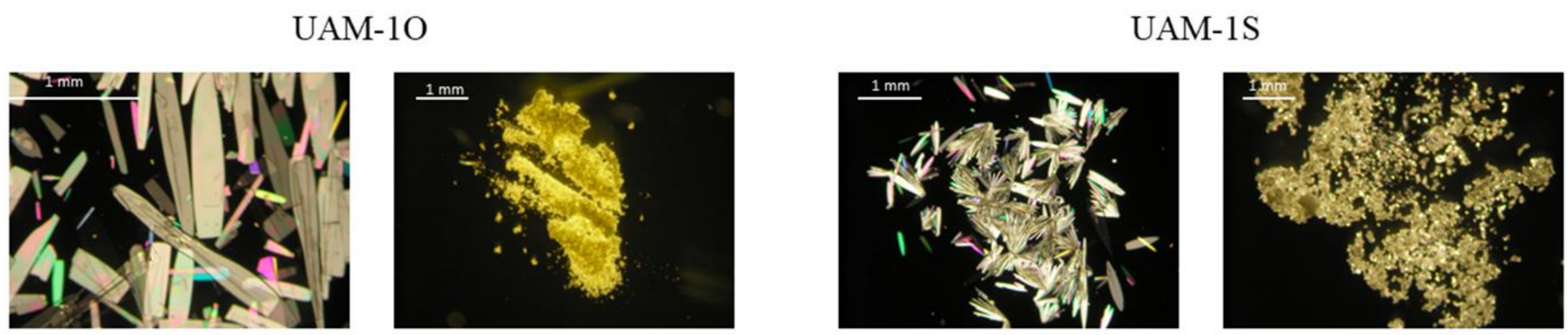

Figure S10. UAM-1X (X: O or S) microscopic images of crystals (with scale bar) after synthesis (left) and after activation (right; DCM exchange; 80 °C and vacuum), captured under polarized light.

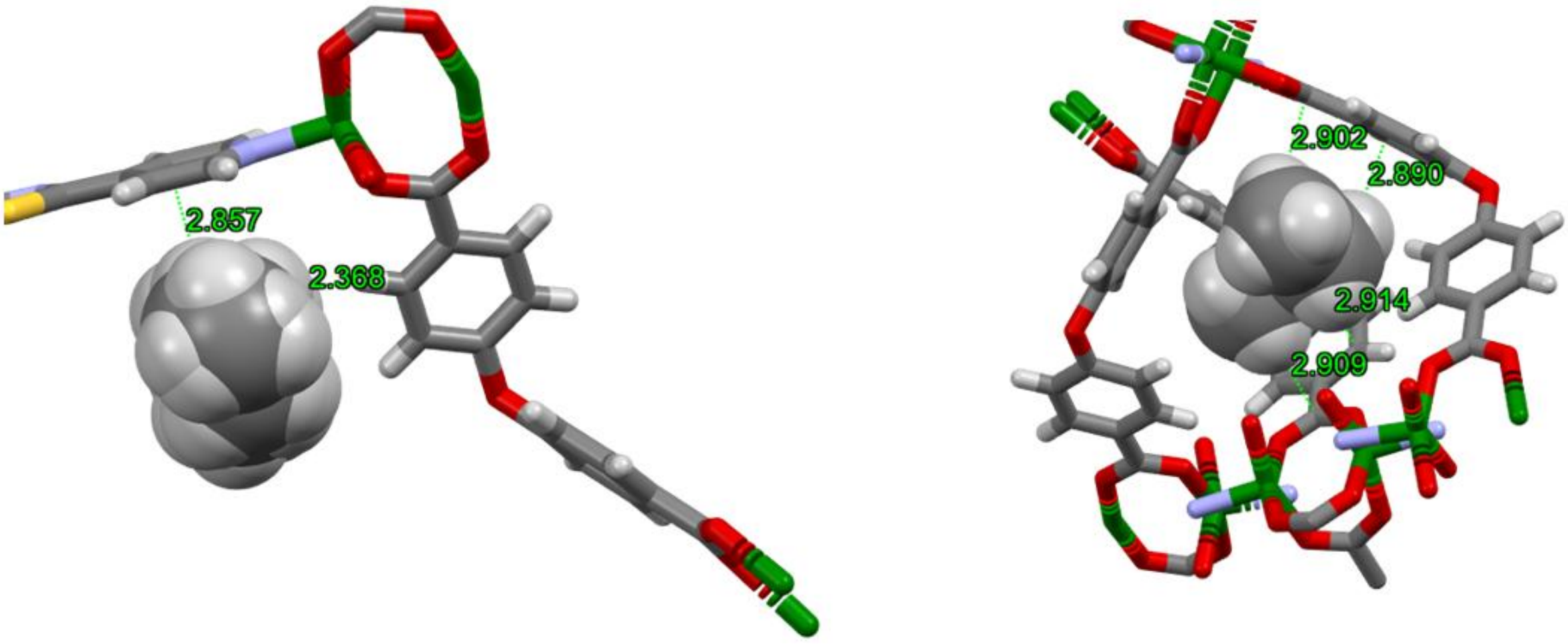

Figure S11 Positions and lengths of the contacts of *n*-butane molecules with UAM-1O across the large pore (left) and across the small pore (right) (Gray-C, Blue-N, Red-O, Yellow- S, Gree*n*- Zn, White- H).

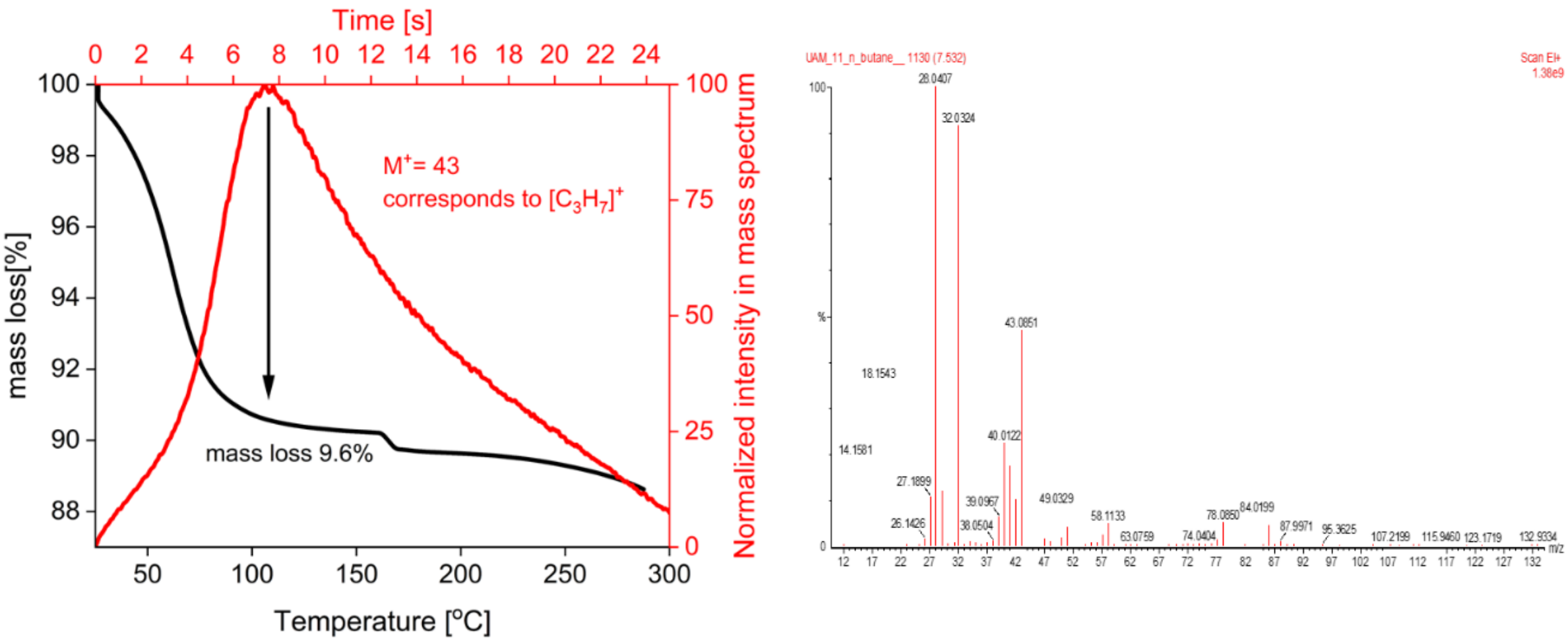

Figure S12. TG-MS analysis of UAM-1O$_{(n\text{-butane})}$.

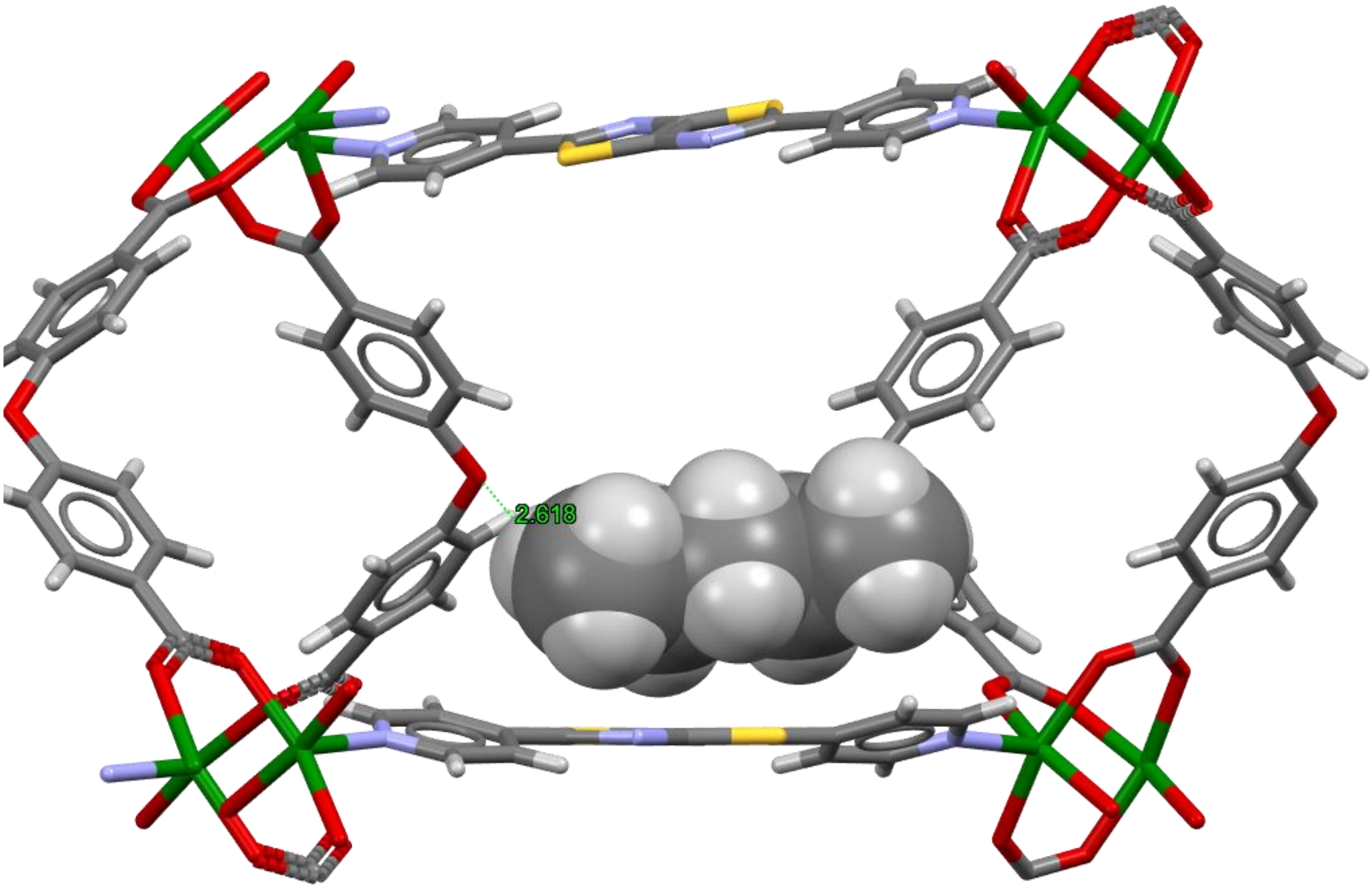

Figure S13 Position and length of the contacts of the *n*-pentane molecule with UAM-1O in the large pore (Gray-C, Blue-N, Red-O, Yellow- S, Gree*n*- Zn, White- H)).

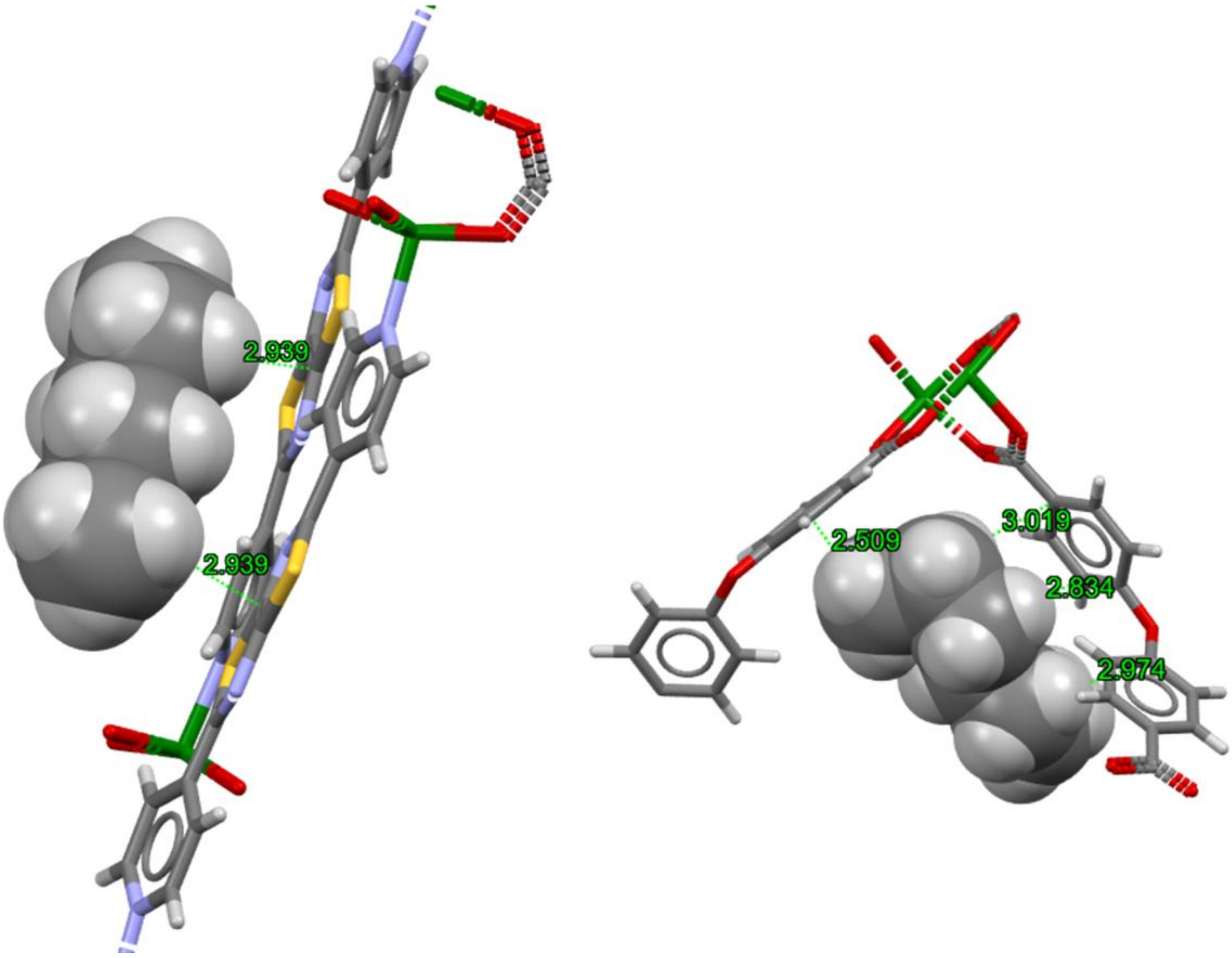

Figure S14. Positions and lengths of the contacts of *n*-hexane molecules with UAM-1O in the large pore (left) and the small pore (right) (Gray-C, Blue-N, Red-O, Yellow- S, Green- Zn, White- H).

Notably, the all-trans *n*-hexane chains are oriented either parallel to the (001) plane or perpendicular to it when adjacent to a GTT *n*-hexane molecule, whose chain segment protrudes beyond the small pore (**Figure S14**).

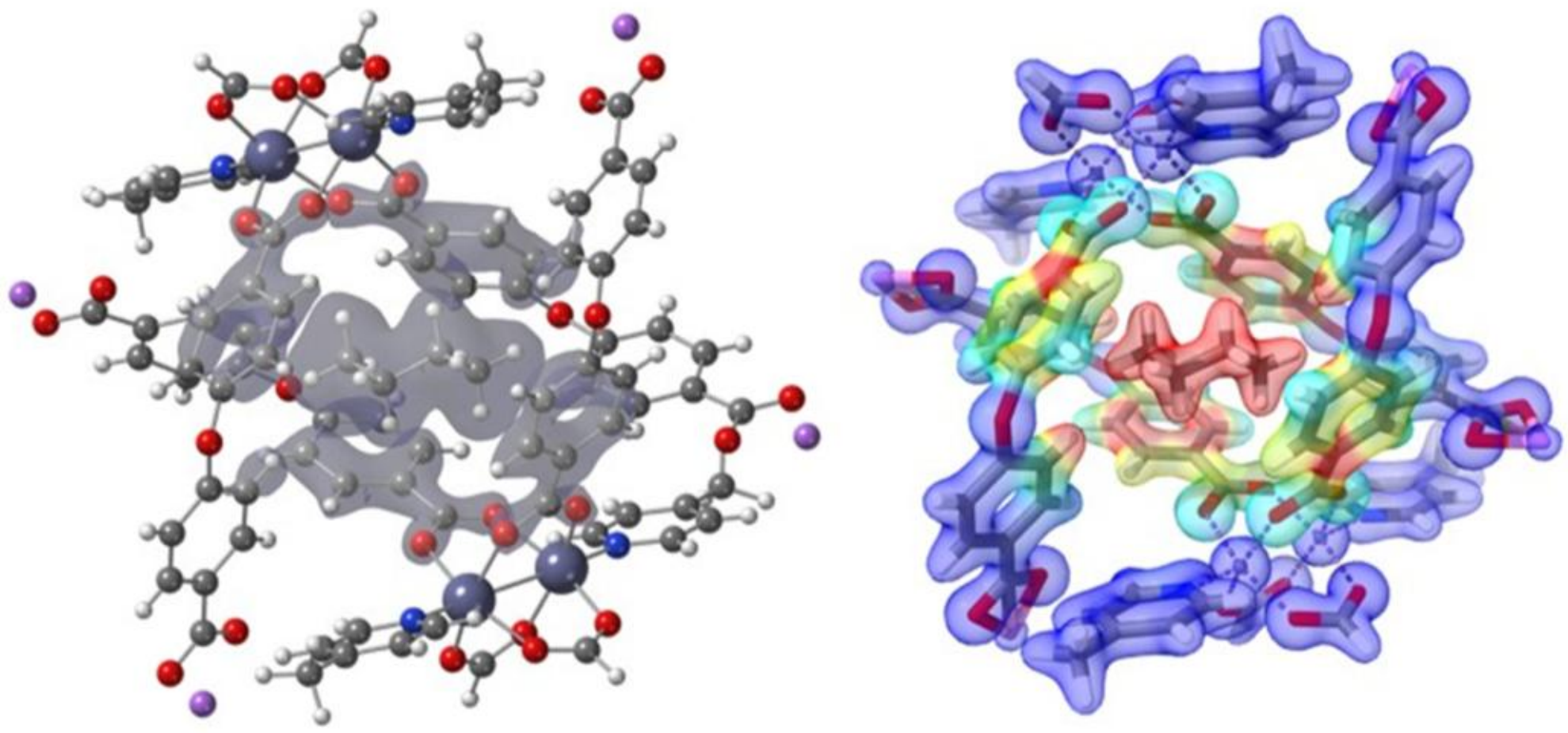

Figure S15. *n*-butane interactions with UAM-1O small pore.

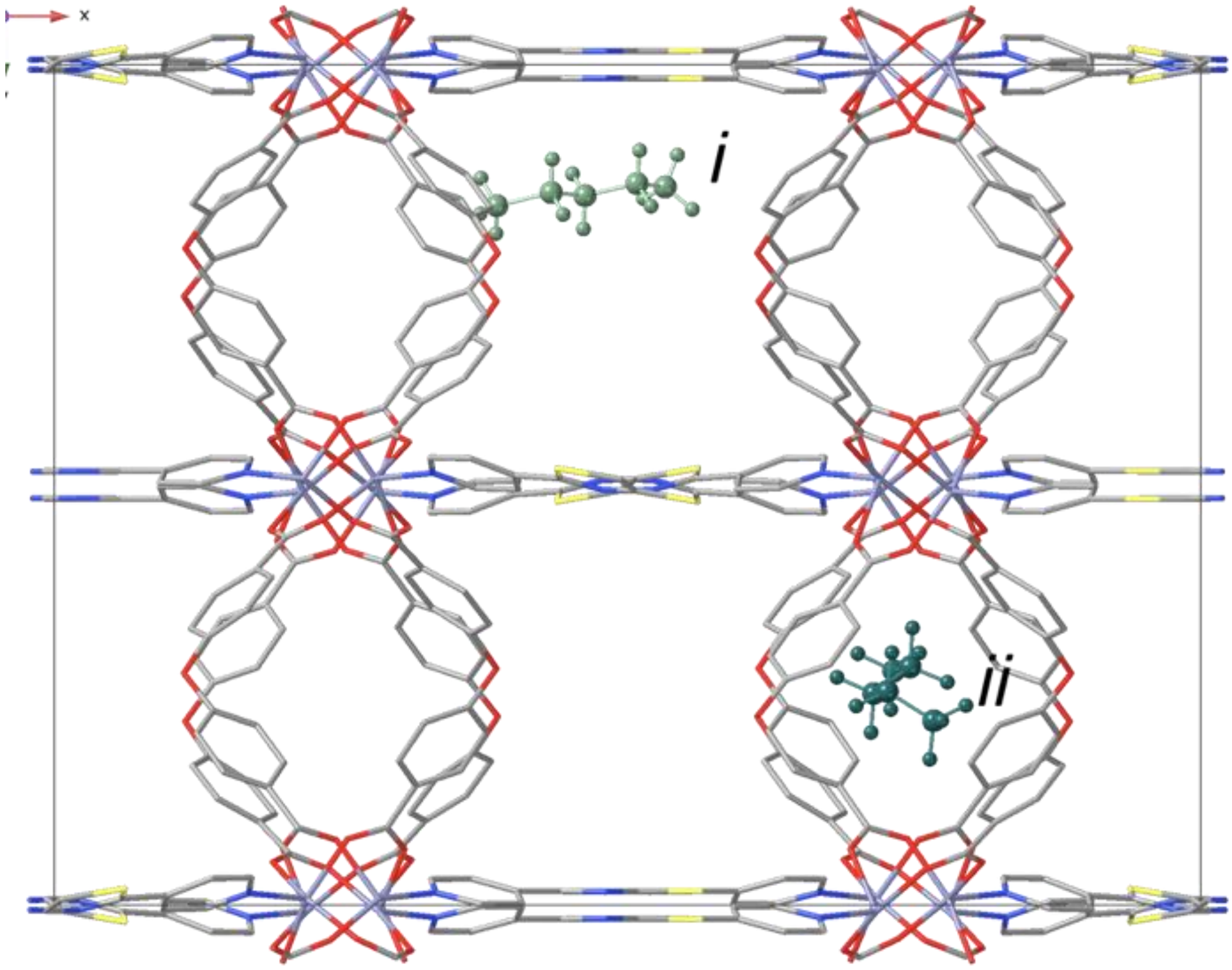

Figure S16. Crystal structure of UAM-1O: with pentane (*i* – *n*-pentane identified experimentally; *ii* – *n*-pentane generated computationally for calculations (Gray-C, Blue-N, Red-O, Yellow- S, Light Blue- Zn; or clarity, hydrogen atoms were removed from the framework structure).

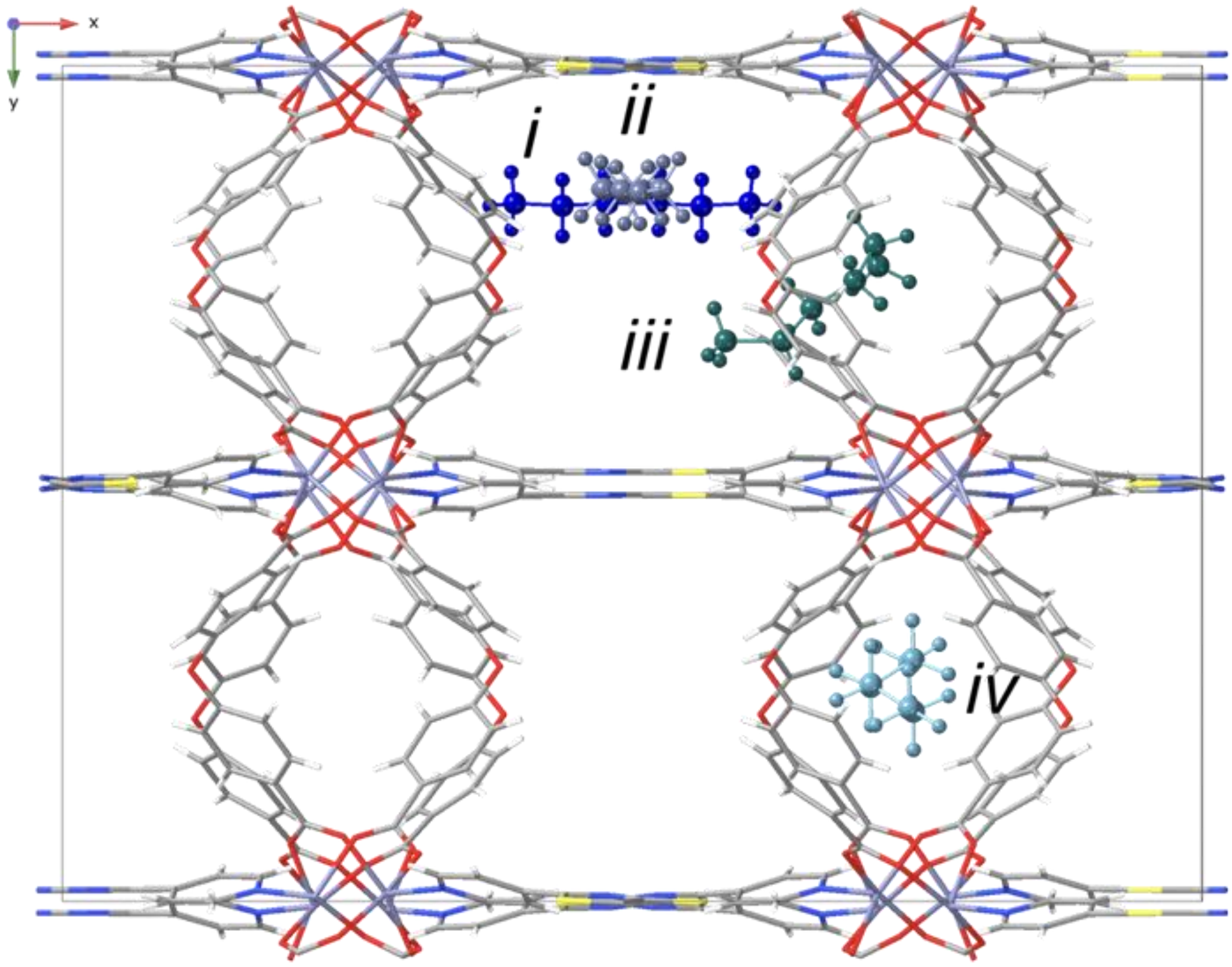

Figure S17. Crystal structure of UAM-1O: with hexane (*i*–*iii* – *n*-hexane identified experimentally; *iv* – *n*-hexane generated computationally for calculations) (Gray-C, Blue-N, Red-O, Yellow- S, Light Blue- Zn; white-H).

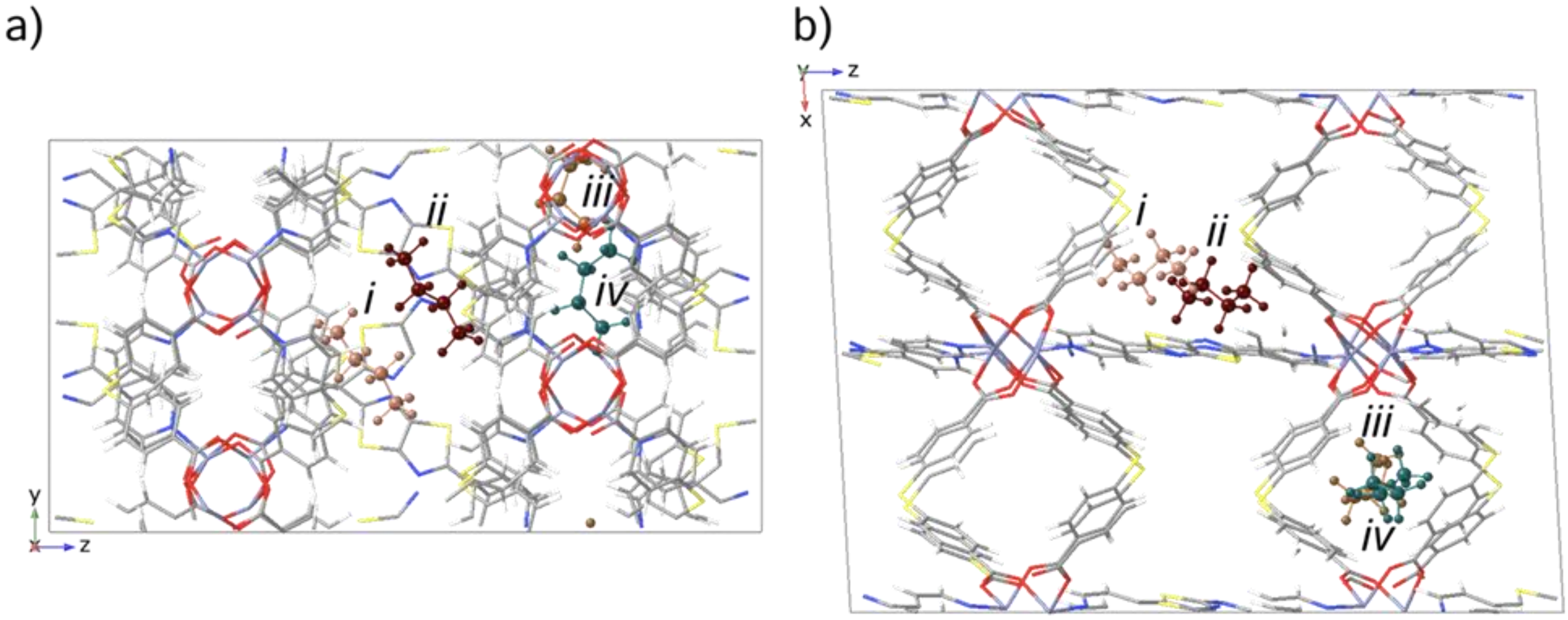

Figure S18. *n*-butane positions in UAM-1S in x-axis view (a), and y-axis view (b). (Gray-C, Blue-N, Red-O, Yellow- S, Light Blue- Zn; white-H).

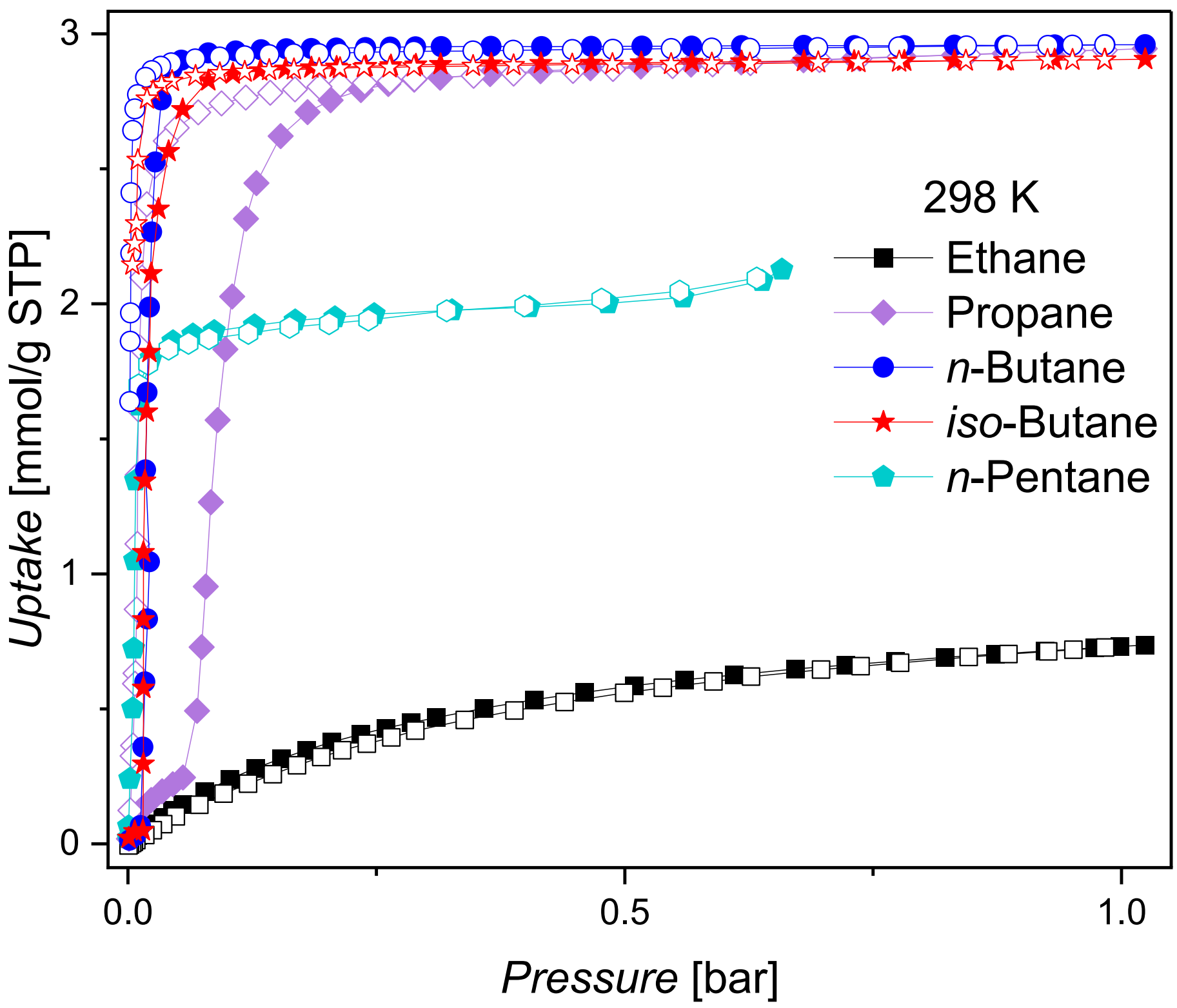

Figure S19. Hydrocarbons adsorption isotherms at 298 K.

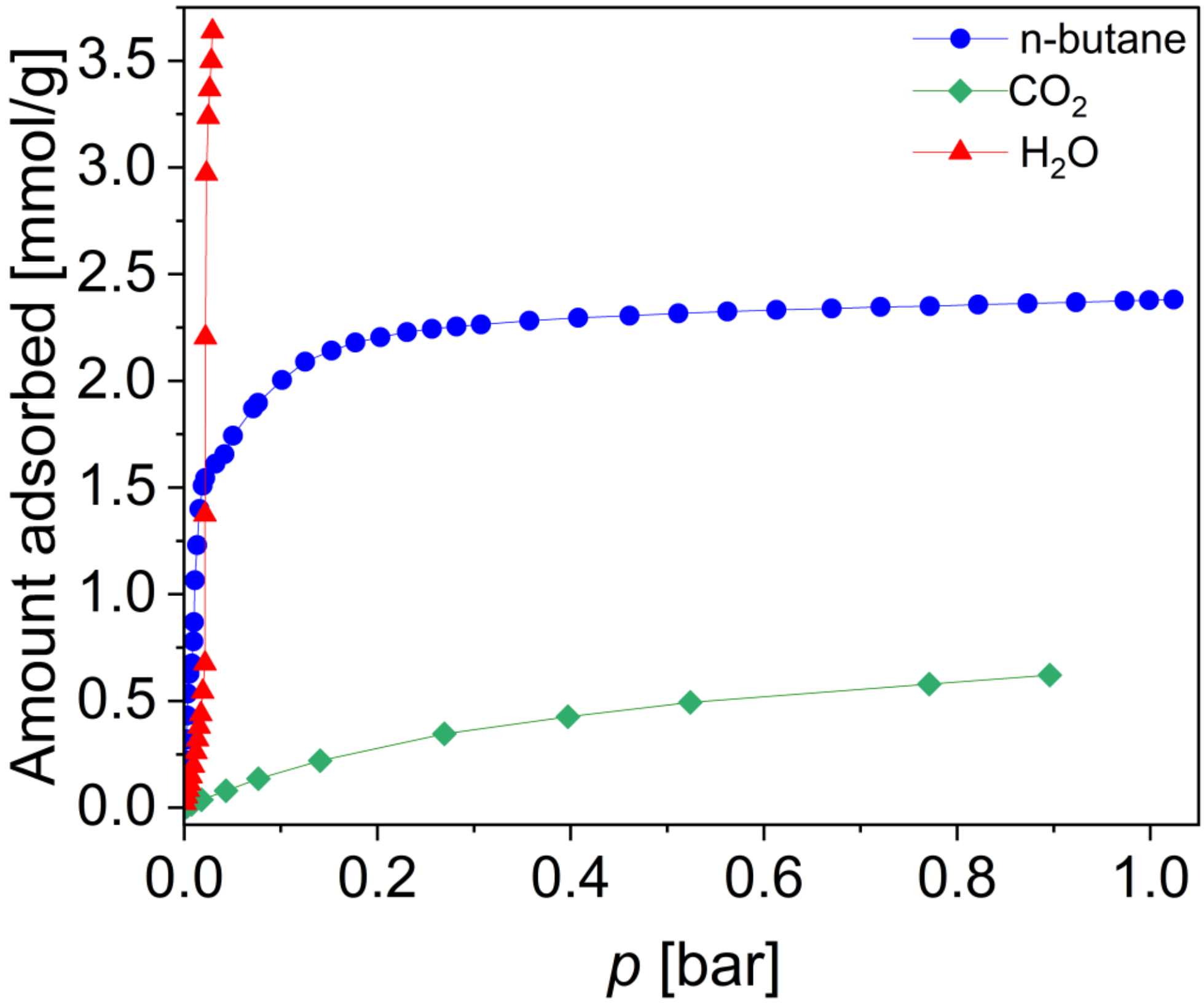

Figure S20. CO_2, H_2O and *n*-butane adsorption isotherms at 298 K.

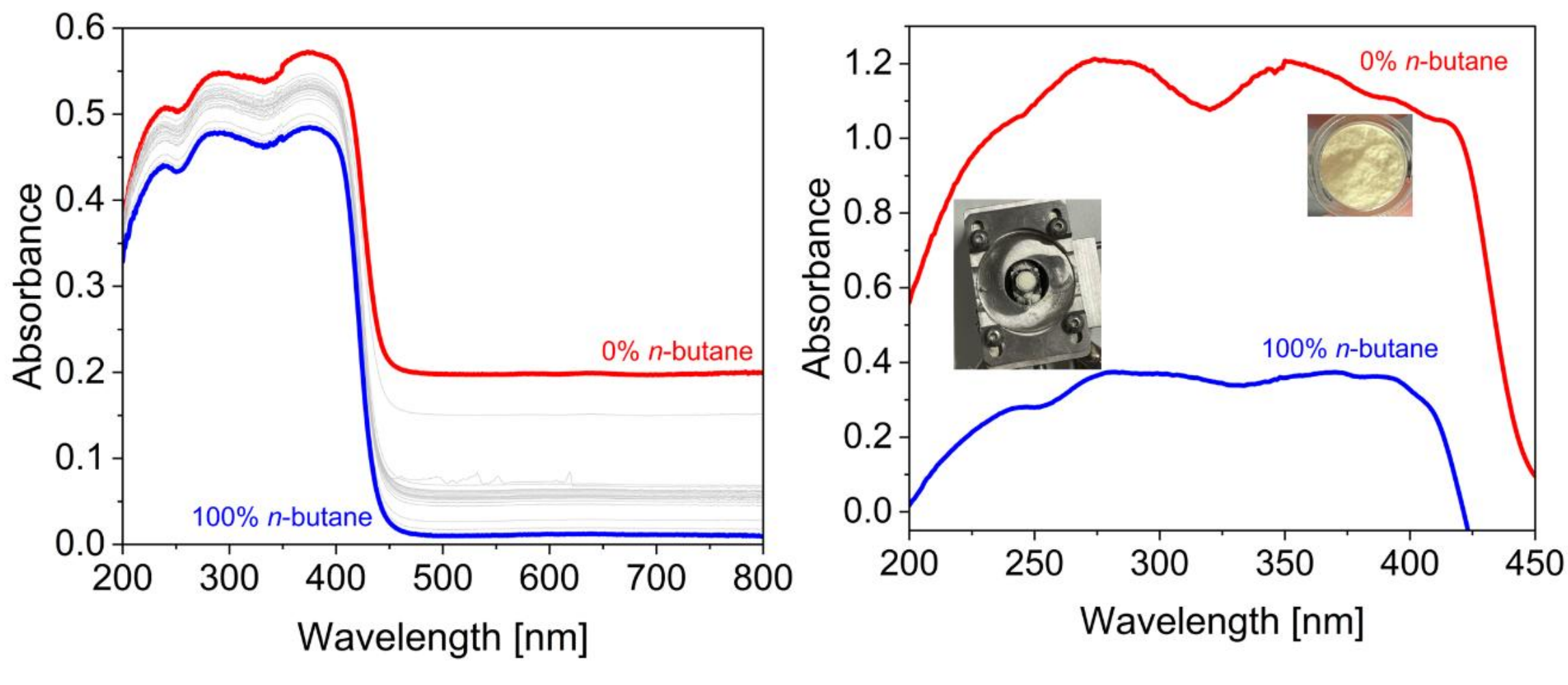

Figure S21. Full *in situ* IV-VIs range during *n*-butane adsorption (UAM-1S + BaSO4) (left). *In situ* UV-vis measured for pure **UAM-1S** with before and after images.

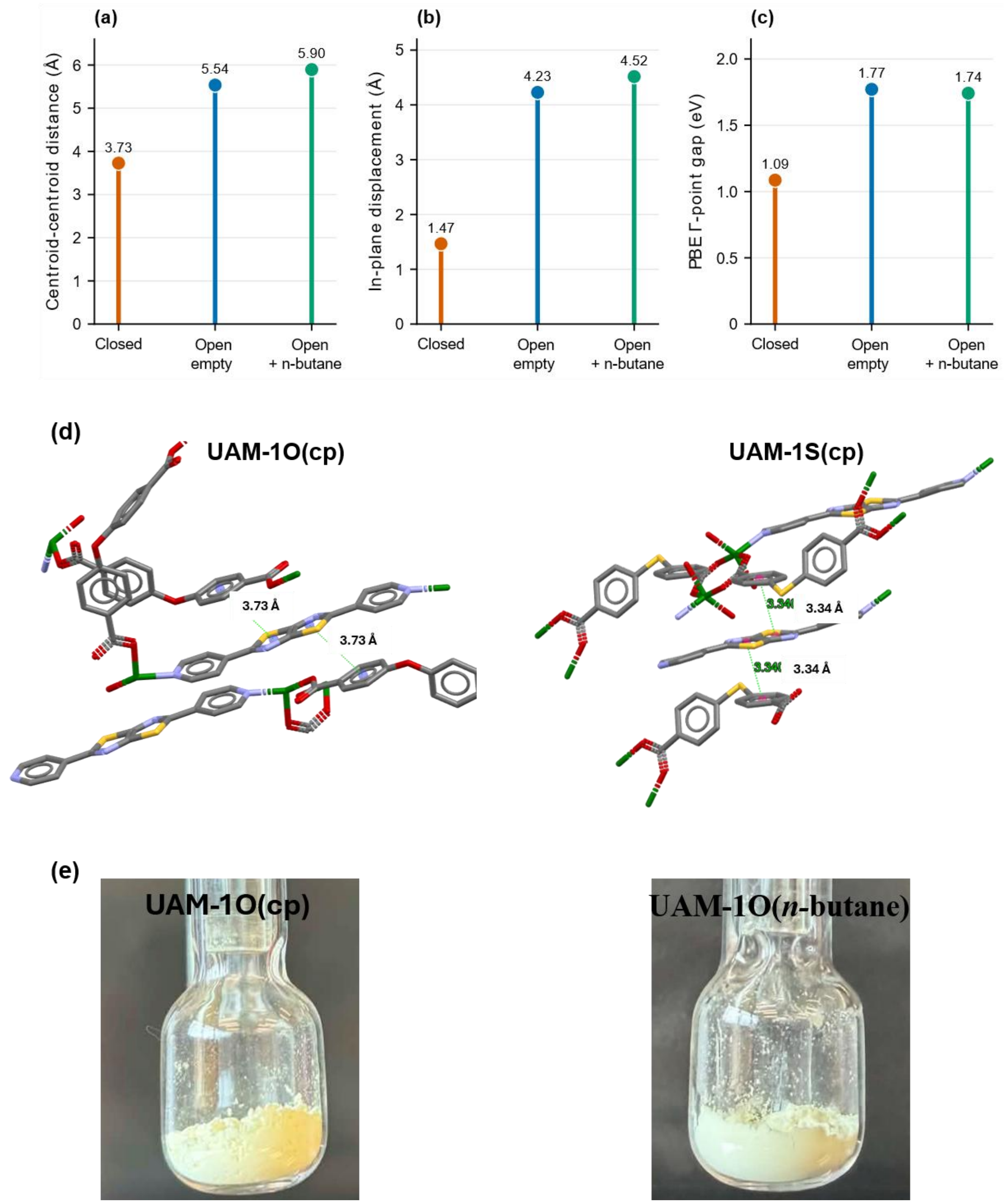

Figure S22. (a) Minimum periodic centroid-centroid distance between a TzTz core and the closest OBA phenyl ring. (b) In-plane displacement of the same contacts, measured relative to the TzTz core plane. (c) Γ-point Kohn-Sham gaps. (d) Representation of the centroids in the crystal structures of UAM-1O and UAM-1S (hydrogen atoms omitted for clarity; carbon, gray; oxygen, red; nitrogen, blue; sulfur, yellow; zinc, green). (e) Color change of the UAM-1O(*cp*) upon adsorption of n-butane at 278 K.

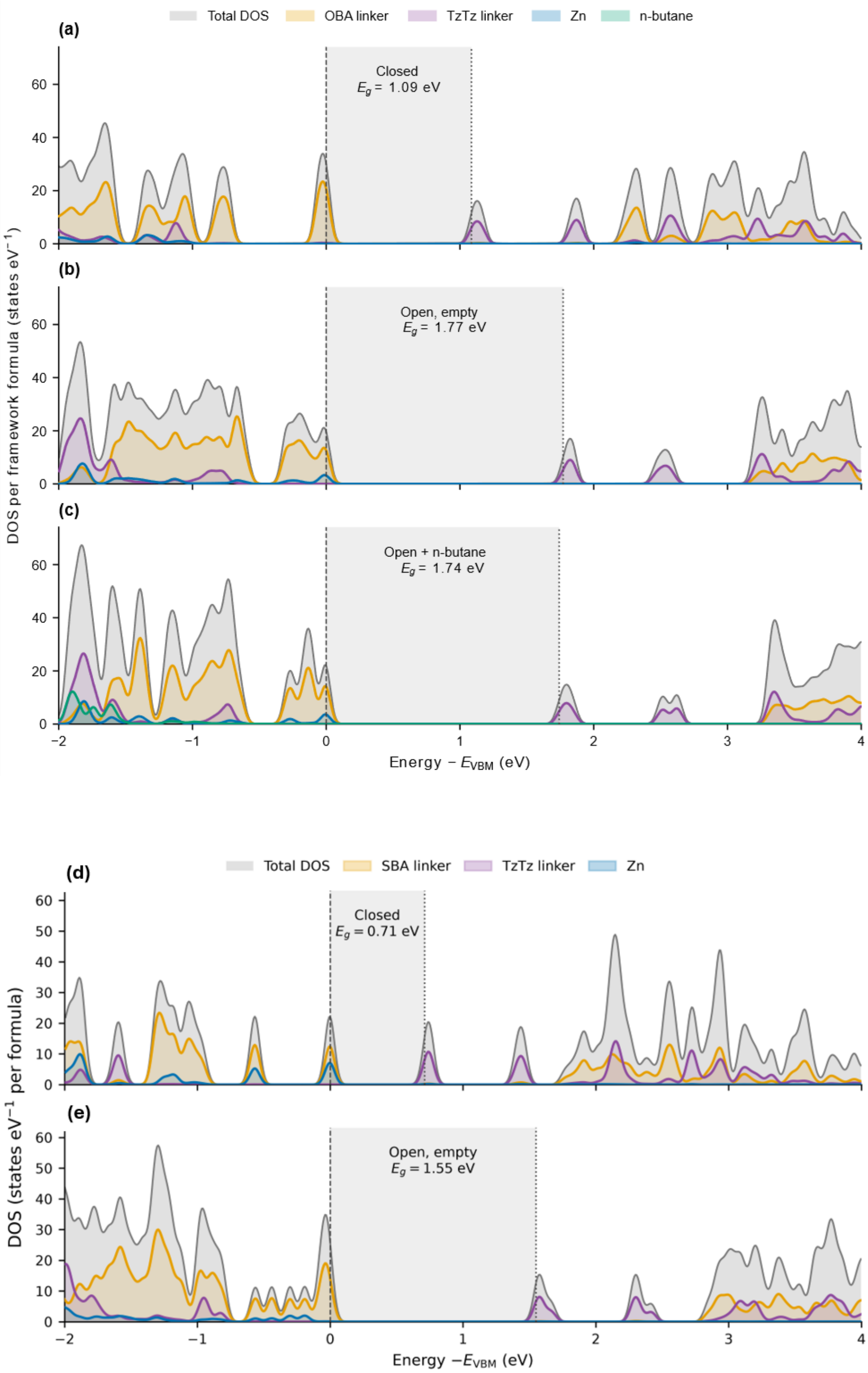

Figure S23. Fragment-projected densities of states for the (a) closed, (b) open (empty), and (c) open (with n-butane) geometries of UAM-1O. Fragment-projected densities of states for the (d) closed, (e) open (empty) UAM-1S. Energies are referenced to the valence-band maximum. Gray shading between the vertical lines marks the Γ-point Kohn–Sham gap. Colored curves and areas show projections onto the OBA or SBA linkers, TzTz linkers, Zn atoms, and n-butane, the gray curve/area is the total density of states. Densities are reported per framework formula unit.

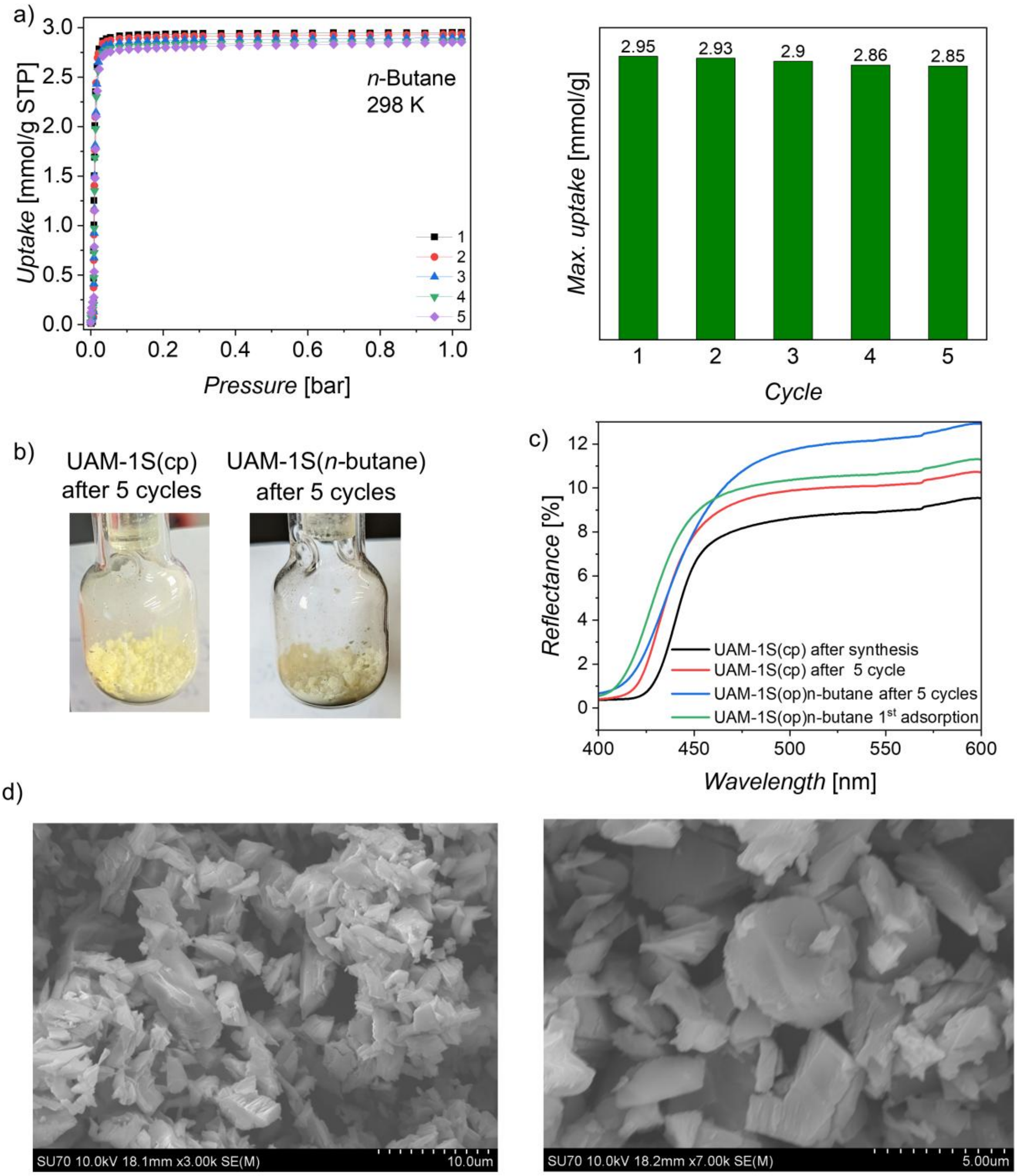

Figure S24. a) *n*-Butane adsorption isotherms measured at 298 K over successive adsorption-desorption cycles (left), with a bar chart indicating the maximum n-butane uptake in each successive cycle (right). b) Photos of the measurement cells containing activated UAM-1S and of the same material after dosing it into the n-butane cell c) Reflective UV-Vis UAM-1S collected for fresh activated sample, the activated sample after five n-butane cycles, and the n-butane loaded sample during the first and sixth cycles **d**) SEM image of UAM-1S(*cp*) recorded after five n-butane adsorption-desorption cycles.

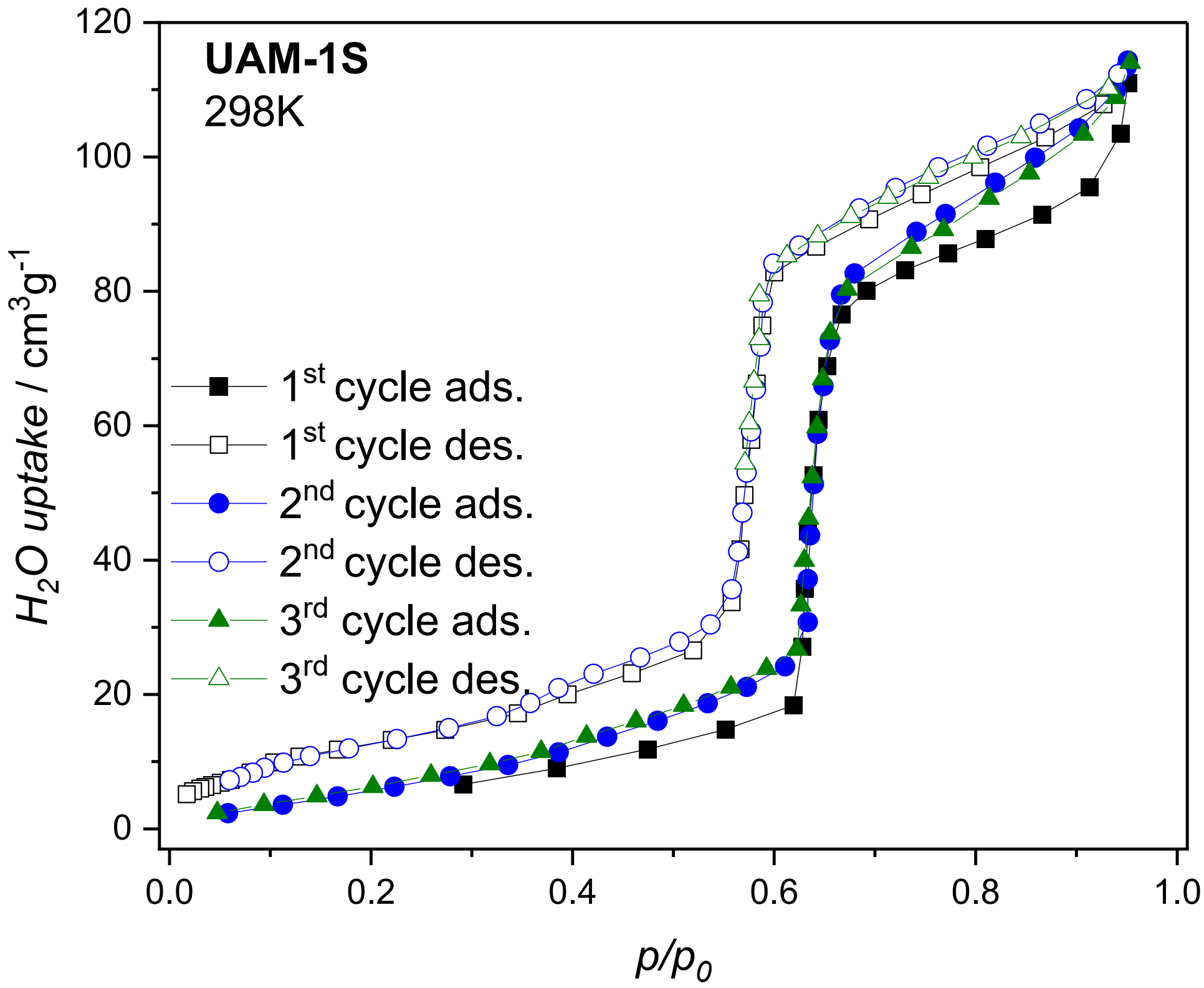

Figure S25. Humidity stability of **UAM-1S** proved by repeatable adsorption and desorption of water vapor at 298K. Data shows that materials are stable in humid conditions. The data were reproduced with the permission of the RSC.[36]

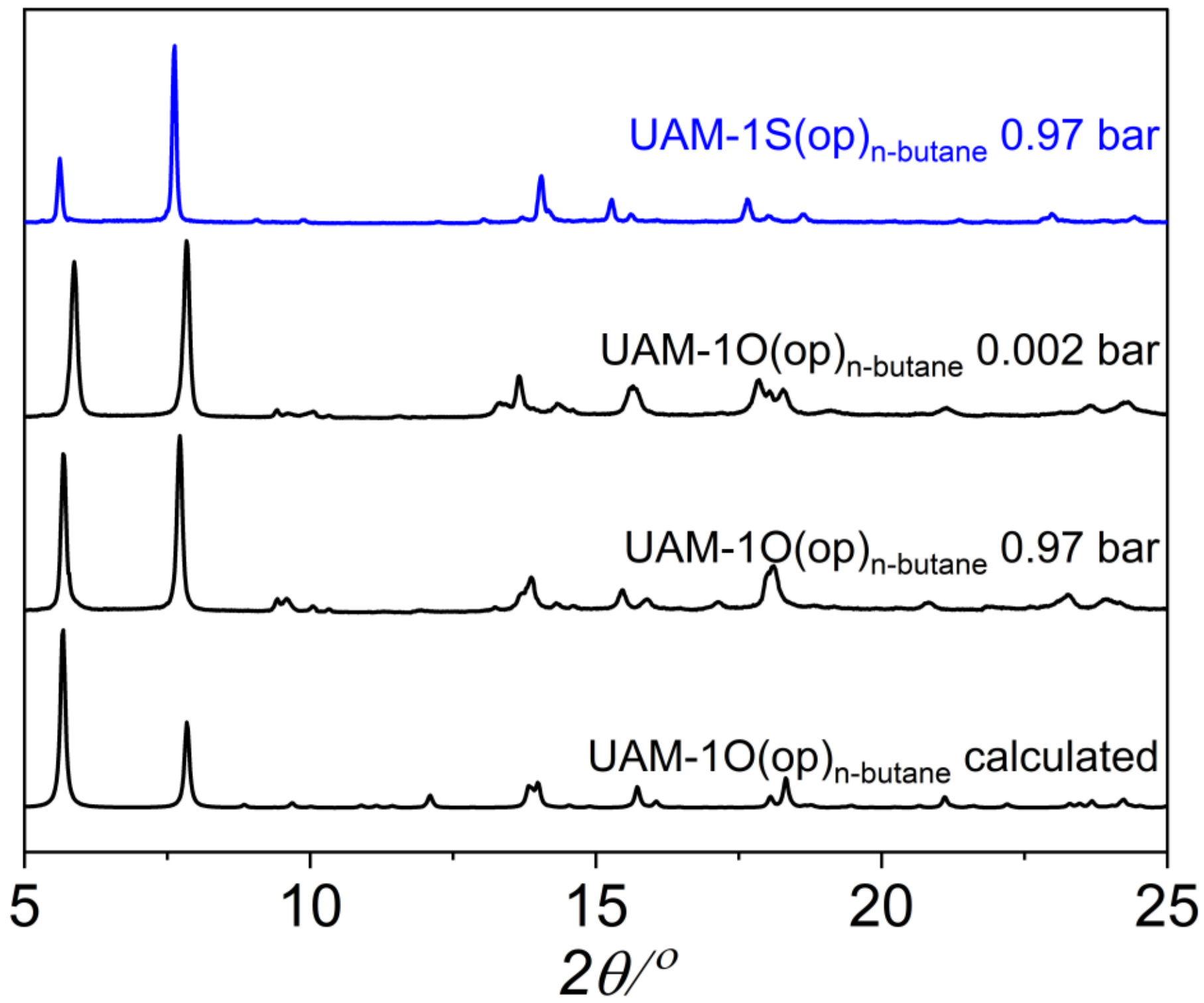

Figure S26. Comparison of the powder diffraction patterns of UAM-1O(*op*)$_{n\text{-butane}}$ and UAM-1S(*op*)$_{n\text{-butane}}$.

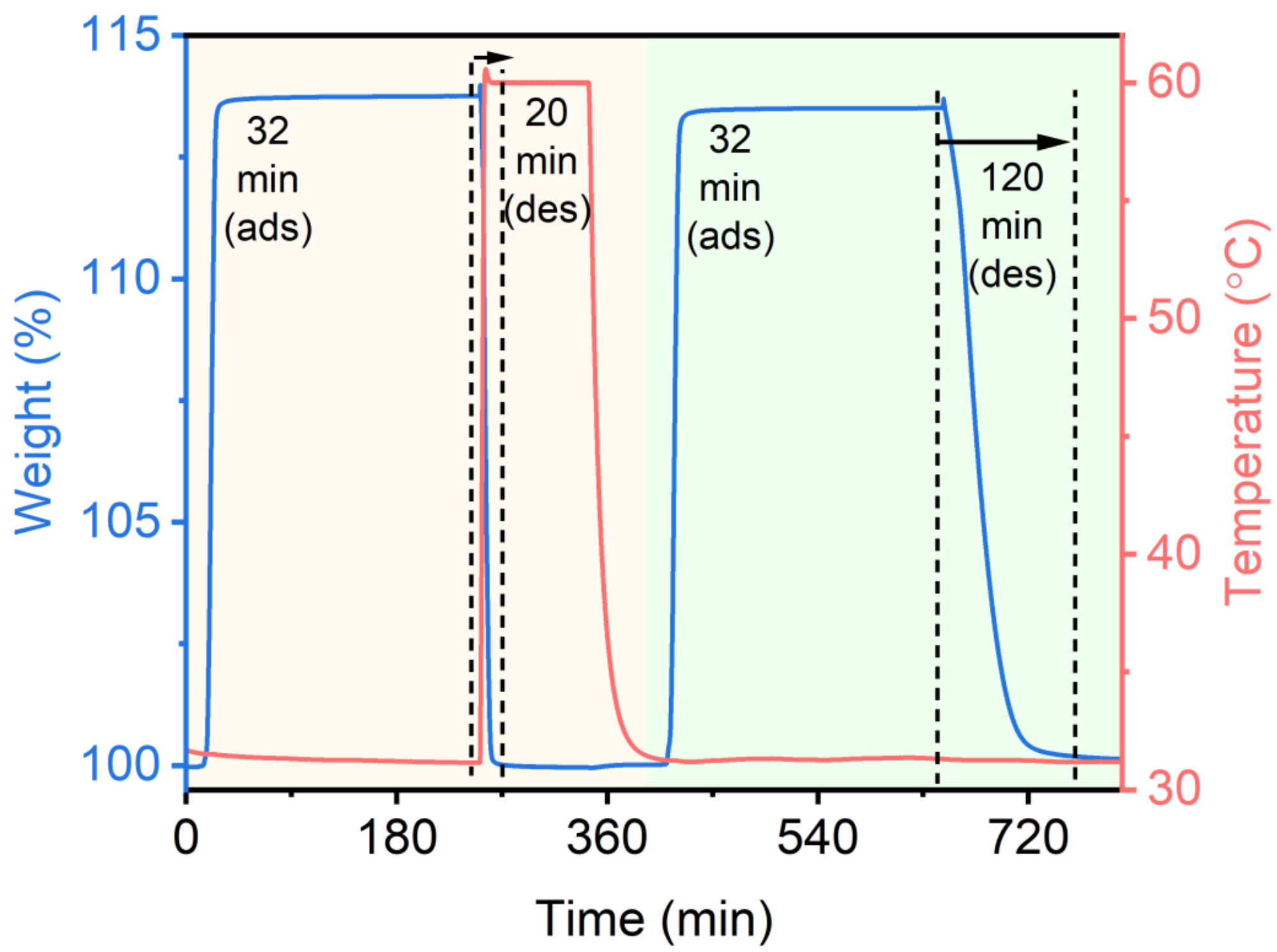

Figure S27. Regeneration study of UAM-1S (through monitoring TGA) over two adsorption–desorption cycles of *n*-C_4H_{10}. Sample weight (blue, left axis) (*ca*. 9 mg) and temperature (red, right axis) were recorded simultaneously. Heating to 60 °C under N_2 flow (60 mLmin^{-1}) enables complete desorption, requiring approximately 20 min. Conversely, 120 min was needed to fully desorb during the second cycle when no heat was applied.

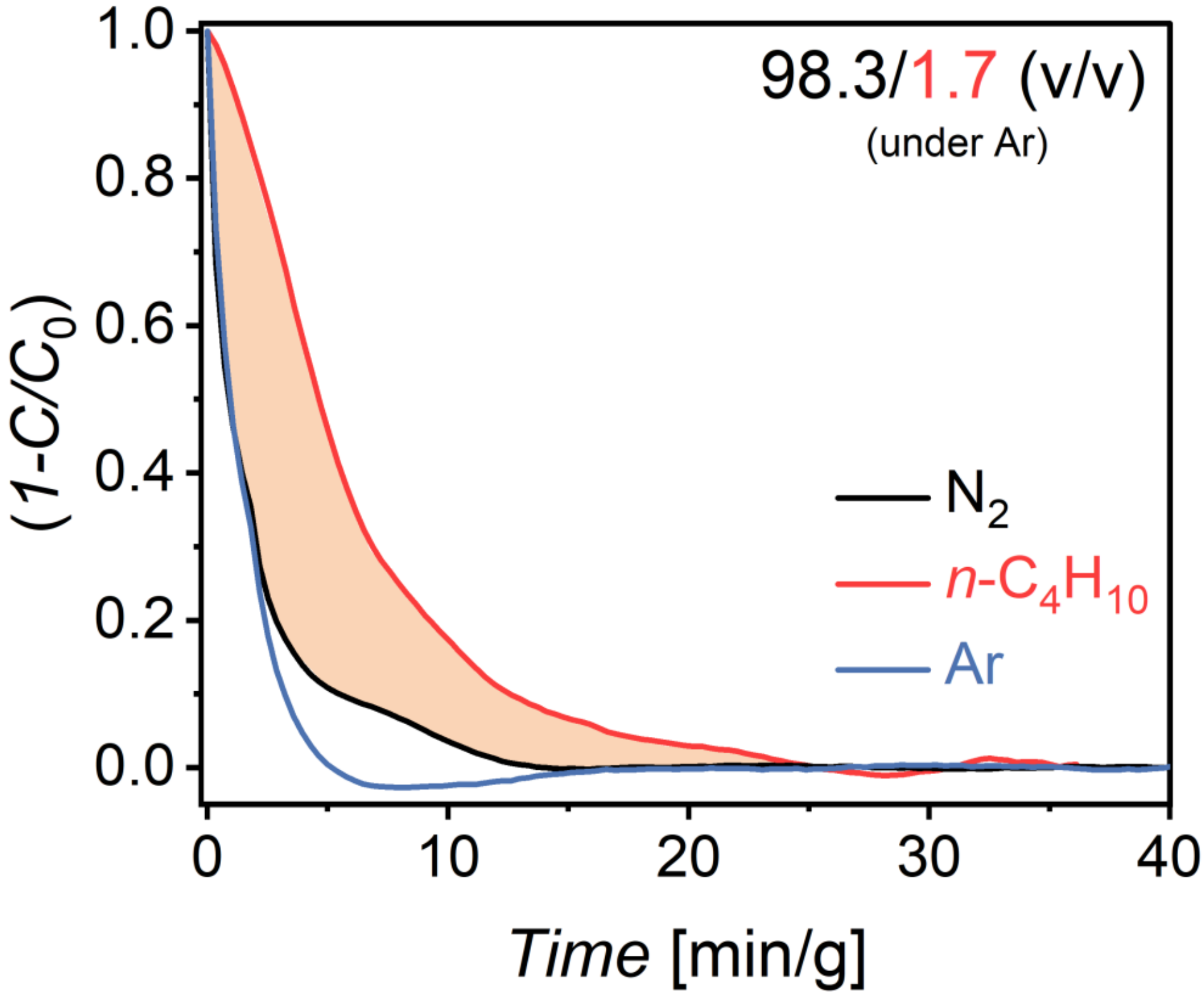

Figure S28. DCB profile for a ternary N_2/*n*-C_4H_{10}/Ar mixture (98.3/1.7 *v/v*, balance Ar) at ambient conditions. Ar was used as the humidity carrier (balance) gas in wet experiments, this control experiment was conducted to decouple the effect of Ar. N_2 and Ar breakthrough rapidly, whereas *n*-C_4H_{10} exhibits a significantly delayed breakthrough, indicating preferential adsorption of *n*-C_4H_{10}. And confirming Ar is no*n*-adsorbing gas. The shaded region highlights the separation window between N_2 and *n*-C_4H_{10}.

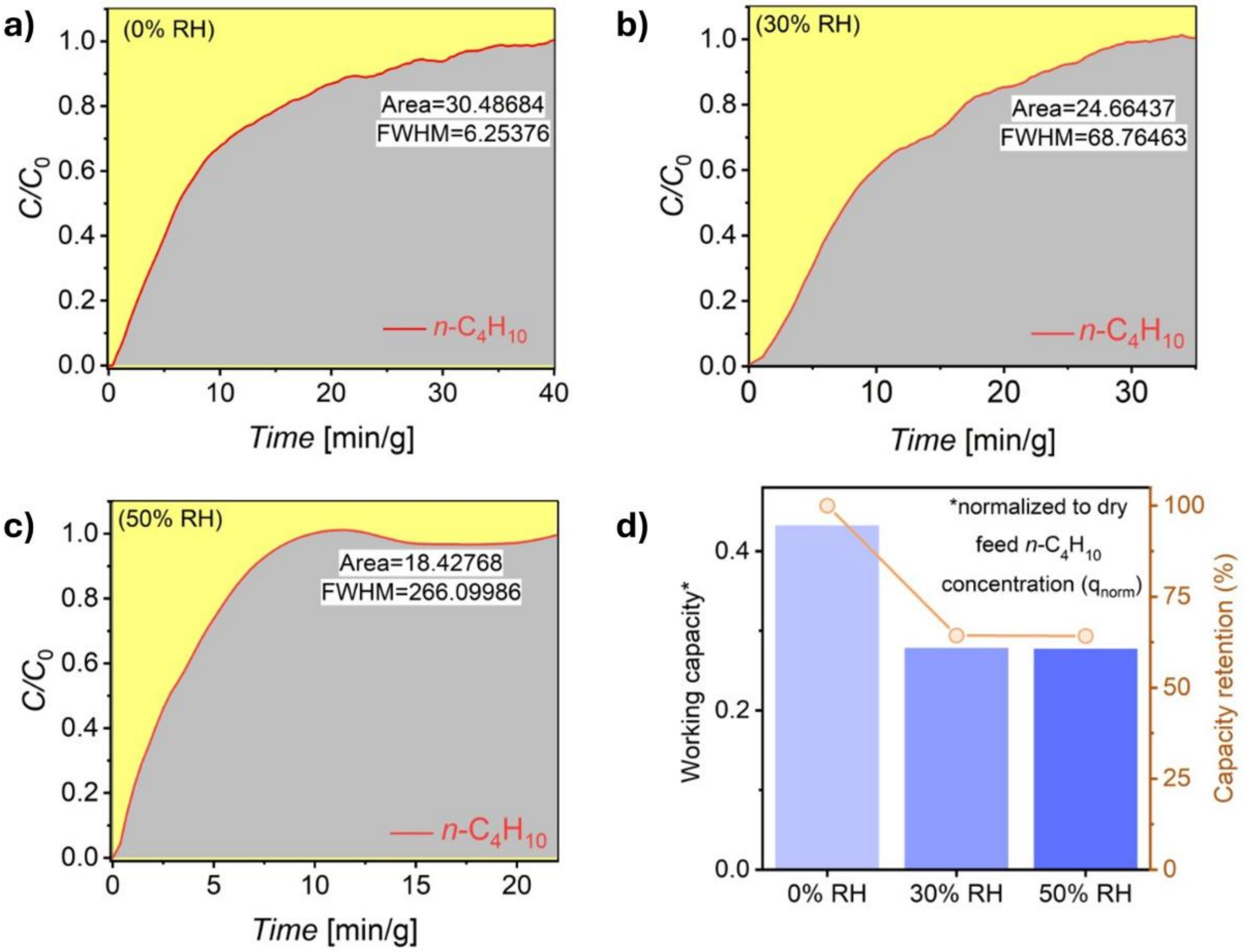

Figure S29. Dynamic column breakthrough (DCB) curves of *n*-C_4H_{10} under dry and humid conditions. The shaded area (grey) represents the integrated breakthrough area used to calculate the dynamic working capacity. (a) Dry breakthrough experiment using a 98.3/1.7 (v/v) N_2/*n*-C_4H_{10} feed (0% RH). (b) Breakthrough experiment using a 95.85/2.92/1.23 (v/v/v) N_2/*n*-C_4H_{10}/H_2O feed (30% RH). (c) Breakthrough experiment using a 94.72/2.74/2.54 (v/v/v) N_2/*n*-C_4H_{10}/H_2O feed (50% RH). (d) Comparison of the working capacities obtained under dry and humid conditions after normalization to the dry-feed n-C_4H_{10} concentration (1.70 vol%). The normalization is to factor in the slightly varying n-C_4H_{10} concentrations caused by different humidity. Capacity retention (plotted on the Y2 axis) is reported relative to the dry experiment.

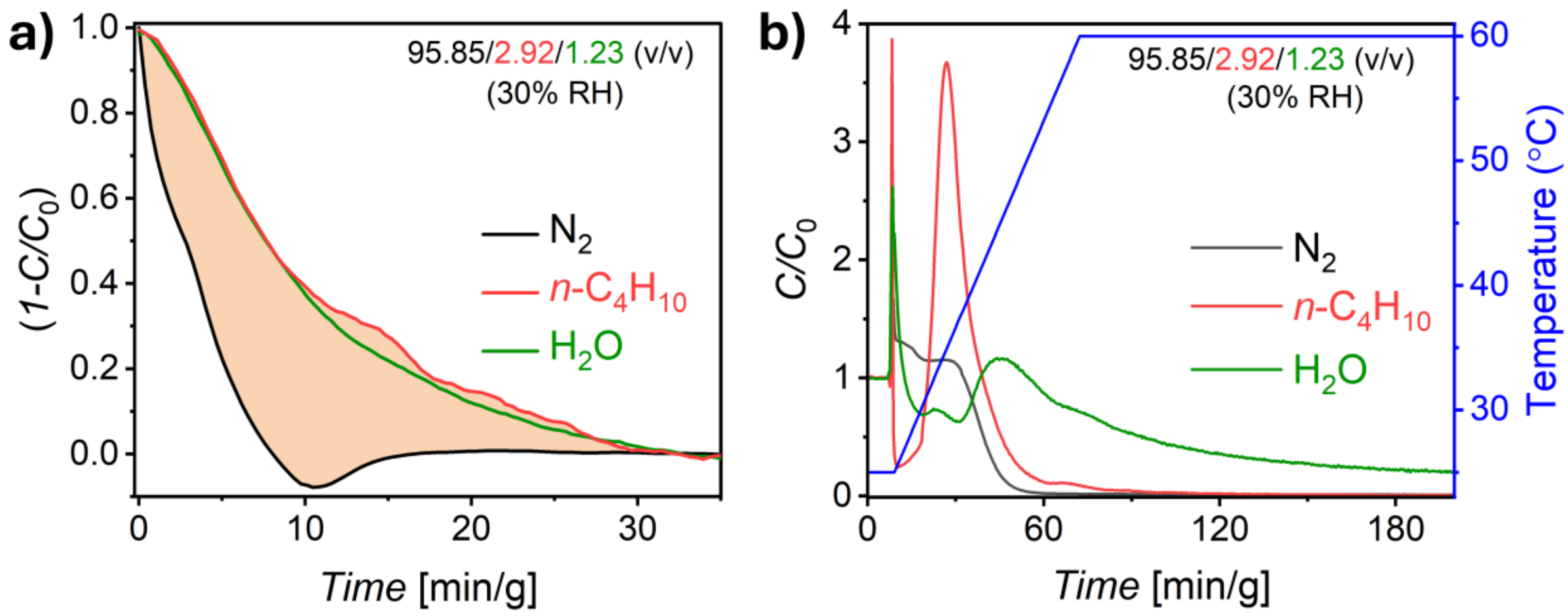

Figure S30. (a) DCB profile for N_2/*n*-C_4H_{10}/H_2O mixture (95.85/2.92/1.23 *v/v*) at 30% RH, showing preferential adsorption of *n*-C_4H_{10} and measurable uptake of H_2O, while N_2 breaks through rapidly. The shaded region highlights the separation window between N_2 and *n*-C_4H_{10}. (b) Corresponding TPD profile following adsorption under the same conditions, revealing distinct

desorption features for N_2, n-C_4H_{10} and H_2O upon heating to 60 °C, consistent with co-adsorption and competitive site occupation.

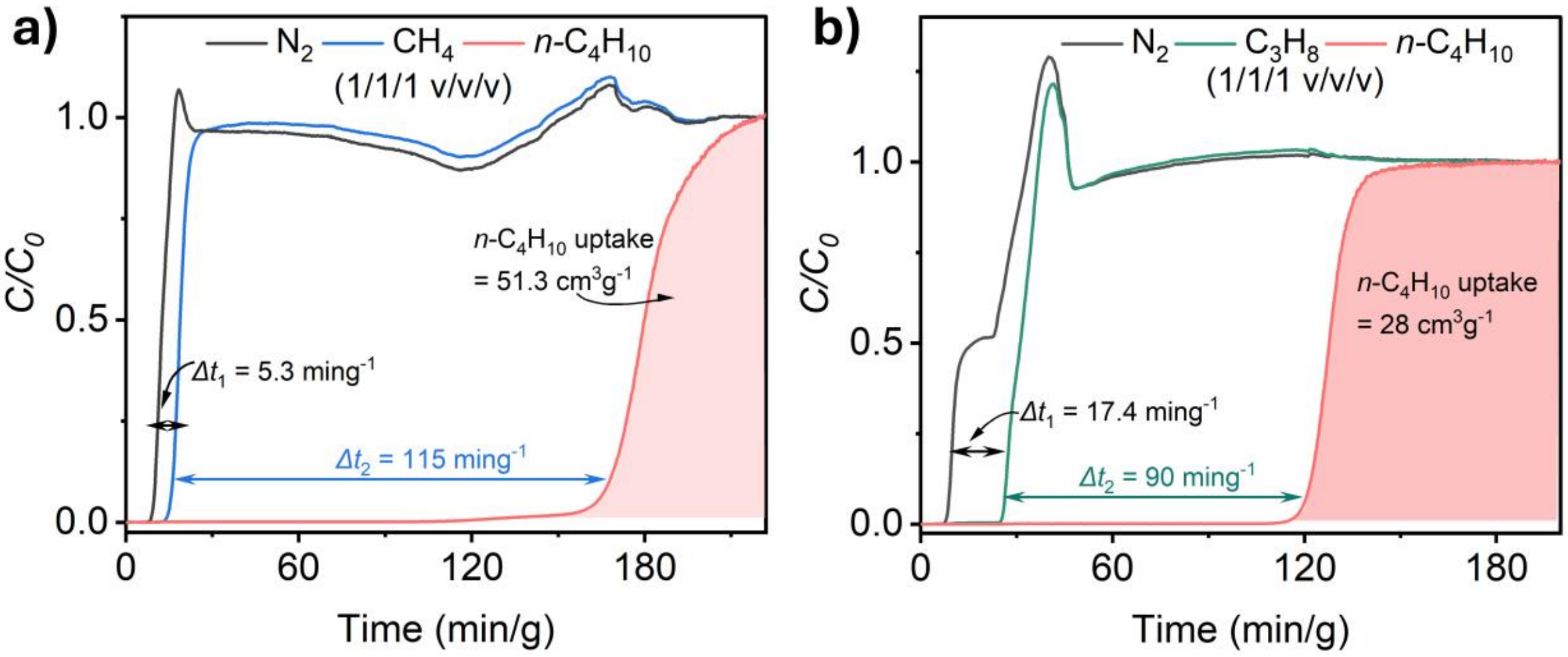

Figure S31. DCB experiments demonstrating the interference resistance of **UAM-1S** toward common volatile alkanes. (a) N_2/CH_4/n-C_4H_{10} (1/1/1, v/v/v) breakthrough experiment at 298 K. (b) N_2/C_3H_8/n-C_4H_{10} (1/1/1, v/v/v) breakthrough experiment at 298 K.

Tables

Table S1. Crystallographic data of UAM-1O(*n*-butane, UAM-1O(*n*-pentane), UAM-1O(*n*-hexane).

	UAM-1O(*n*-butane)	UAM-1O(*n*-pentane)	UAM-1O(*n*-hexane)
CCDC	2537888	2537889	2537890
Empirical formula	$C_{49}H_{41.5}N_4O_{10}S_2Zn_2$	$C_{45.33}H_{32}N_4O_{10}S_2Zn_2$	$C_{51.6}H_{46.4}N_4O_{10}S_2Zn_2$
Formula weight	1041.22	987.64	1077.38
Temperature/K	100	100	100
Crystal System	Orthorhombic	Orthorhombic	Orthorhombic
Space group	*P*bcn	*P*bcn	*P*bcn
a/*b*/*c* [Å]	31.137(2)/ 22.522(15)/ 15.9582(10)	31.124(6)/ 22.826(5)/ 16.020(3)	31.129(3)/ 22.844(3)/ 15.9546(17)
α/ *β*/ *γ* [°]	90/90/90	90/90/90	90/90/90
Volume/Å^3	11191.0(13)	11382(4)	11345(2)
Z	8	8	8
d [g/cm^3]	1.236	1.153	1.262
Radiation type	MoKα (λ= 0.71073)	MoKα (λ= 0.71073)	MoKα (λ= 0.71073)
μ/mm^{-1}	0.985	0.965	0.974
F(000)	4284.0	4032.0	4384.0
Crystal size/mm	0.42 × 0.21 × 0.18	0.53 × 0.19 × 0.08	0.45 × 0.20 × 0.11
2Θ range for data collection/°	4.078 to 51.414	4.062 to 50.624	4.312 to 52.044
Index ranges	-37≤ h ≤ 37, -27 ≤ k ≤ 27,-19 ≤ l ≤ 19	-37≤ h ≤ 37, -27 ≤ k ≤ 27, -19 ≤ l ≤ 19	-38≤ h ≤ 36, -28≤ k ≤ 27,-19 ≤ l ≤ 19
Reflections collected	176827	350041	140790
Independent reflections	10645 [R_{int}=0.0549, R_{sigma}=0.0271]	10174 [R_{int}=0.1457, R_{sigma}=0.0489]	11157 [R_{int}=0.1345, R_{sigma}=0.0730]
Data/restraints/parameters	10645/10/615	10174/8/585	11157/24/645
Goodness-of-fit on F^2	1.055	1.000	1.056
Final R indexes [I>=2σ (I)]	R_1= 0.0434, wR_2= 0.1215	R_1= 0.0655, wR_2= 0.1877	R_1= 0.0744, wR_2= 0.1830
Final R indexes [all data]	R_1=0.0581. wR_2=0.1343	R_1=0.1029. wR_2=0.2255	R_1=0.1172. wR_2=0.2088
Largest diff. peak/hole /eÅ^{-3}	0.78/-0.45	1.19/-0.87	1.48/-0.53

Table S2. Selected structural information on investigated UAM-1O$_{(n\text{-hydrocarbons})}$ and UAM-1O$_{(DMF)}$[37]

	**UAM-1O(*n*-butane)**	**UAM-1O(*n*-pentane)**	**UAM-1O(*n*-hexane)**	**UAM-1O(DMF)**
Z*n*-O [Å]	2.026-2.061	2.017-2.055	2.007-2.066	2.027-2.068
Z*n*-N [Å]	2.018-2.021	2.023-2.025	2.021-2.024	2.017-2.030
$l_{Zn\text{-}Zn}$ [Å]	2.873	2.878	2.872	2.868
C-X-C [°]	115.07-115.47	116.68-117.17	115.30-116.65	115.2
Length of MOF–hydrocarbon contacts [Å]	2.368-2.914	2.618	2.509-3.019	-

Table S3. Calculated interaction energies for different positions of hydrocarbons in UAM-1). * were created artificially. Please refer to **Figures S15** and **S16**.

Molecule	butane			pentane		hexane			
Conformation	*i*	*ii*	*iii*	*i*	*ii**	*i*	*ii*	*iii*	*iv**
E_{comp} (kJ/mol)	-53.2	-53.2	-75.8	-67.0	-69.3	-74.3	-80.9	-84.3	-56.0
E_{comp} per CH_n (kJ/mol)	-13.3	-13.3	-18.9	-13.4	-13.9	-12.4	-13.5	-14.1	-9.3

Table S4. Components of the LED analysis of the interaction energy of the fragments. Unit of the energy is kJ/mol.

$\Delta E^{\text{ref.}}_{\text{el. prep.}}$	$E^{\text{ref.}}_{\text{electrost.}}$	$E^{\text{ref.}}_{\text{exch.}}$	$\Delta E^{C-CCSD.}_{non-disp.}$	$E^{C-CCSD.}_{disp.}$	$\Delta E^{C-(T).}_{int.}$	Sum
152.2	-83.8	-37.9	-6.4	-82.0	-10.0	-68.1

el. prep. – electronic preparation; electrost. – electrostatics; exch. – exchange; non disp. – non-dispersive correlation; disp. – dispersion; int. – perturbative triples

Table S5. Calculated interaction energies for different positions of *n*-butane within UAM-1S. Gas molecules were artificially put in location corresponding to the observed adsorption in both pores of UAM-1O. Please refer to **Figure S17**.

Conformation	*i*	*ii*	*iii*	*iv*
E_{comp} (kJ/mol)	-53.1	-53.1	-50.2	-66.3
E_{comp} per CH_n (kJ/mol)	-13.3	-13.3	-12.6	-16.6

Table S6. Comparison of total n-C_4H_{10} uptake for different materials at 1 bar. Adsorption temperatures are given in brackets.
* isotherm were recorded on *the fresh* sample,
** isotherm were recorded on the sample that had been stored in sealed vials for approximately 2 years.

Material	*n*-butane uptake [mmol/g] at ~1bar	Reference
**UAM-1O**	3.61 (273 K)/ 0.29 (298 K)	This work
**UAM-1S**	2.99 (273 K)/2.93* or 2.50** (298 K)	This work
**MIL-101**	11.2 (293 K)	38
**Cu_3btc_2**	3.7 (293 K)	38
**Y-fcu-MOF**	1.97 (293 K)	39
**ZIF-8**	4 (293 K)	40
**CALF-20**	2.87 (298 K)	41
**DUT-8(Ni)**	7.1 (272.5 K)	42
**IPM-101**	2.3 (298 K)	43
**FJI-H38**	2.4 (283 K)	44
**DUT-49(Cu)**	10 (298 K)	45
**Znpyc-CF_3**	2.48 (298 K)	46
**ZJNU-30**	11.83 (298K)	47
**Zr-fum-20**	3.2 (298 K)	48
**CMS-PMOF-1**	1.9 (298 K)	49
**Y-fum**	2 (298 K)	49
**[$Ni_3(HCOO)_6$]**	1.67 (298 K)	50
**MIL-53(Al)**	3.5 (298 K)	51
**Co-bpy-bpdc**	1.67 (298 K)	52
**[$Co_3(HCOO)_6$]**	1.77 (298 K)	53
**Ag-hmpba**	0.9 (298 K)	54

Presented data indicate that the n-butane uptake of UAM-1S is not the highest but still competitive. For example, UAM-1S adsorbs only 2.9 mmol g^{-1} of n-butane at 298 K, which is three times lower than that of materials such as DUT-49, DUT-8, MIL-101, or ZJNU-30. While DUT materials are flexible, these materials are moisture-unstable. On the other hand, MIL-101 and ZJNU-30 adsorb most of the n-butane only at relatively high pressures do not show desirable flexibility. Therefore, when considering all key parameters, including adsorption capacity, n-butane retention, and colour change induced by structural transformation, UAM-1S appears to be one of the most promising materials for the dual applications presented in this work.

References

(1) Krause, L.; Herbst-Irmer, R.; Sheldrick, G. M.; Stalke, D. Comparison of Silver and Molybdenum Microfocus X-Ray Sources for Single-Crystal Structure Determination. *J. Appl. Crystallogr.* **2015**, *48* (1), 3–10. https://doi.org/10.1107/S1600576714022985.

(2) Sheldrick, G. M. It SHELXT – Integrated Space-Group and Crystal-Structure Determination. *Acta Crystallogr. Sect. A* **2015**, *71* (1), 3–8. https://doi.org/10.1107/S2053273314026370.

(3) Sheldrick, G. M. Crystal Structure Refinement with SHELXL. *Acta Crystallogr. Sect. C Struct. Chem.* **2015**, *71* (Pt 1), 3–8. https://doi.org/10.1107/S2053229614024218.

(4) Barbour, L. J. It X-Seed 4: Updates to a Program for Small-Molecule Supramolecular Crystallography. *J. Appl. Crystallogr.* **2020**, *53* (4), 1141–1146. https://doi.org/10.1107/S1600576720007438.

(5) Dolomanov, O. V.; Bourhis, L. J.; Gildea, R. J.; Howard, J. A. K.; Puschmann, H. It OLEX2: A Complete Structure Solution, Refinement and Analysis Program. *J. Appl. Crystallogr.* **2009**, *42* (2), 339–341. https://doi.org/10.1107/S0021889808042726.

(6) Bourhis, L. J.; Dolomanov, O. V.; Gildea, R. J.; Howard, J. A. K.; Puschmann, H. The Anatomy of a Comprehensive Constrained, Restrained Refinement Program for the Modern Computing Environment – It Olex2 Dissected. *Acta Crystallogr. Sect. A* **2015**, *71* (1), 59–75. https://doi.org/10.1107/S2053273314022207.

(7) Kieffer, J.; Valls, V.; Blanc, N.; Hennig, C. New Tools for Calibrating Diffraction Setups. *J. Synchrotron Radiat.* **2020**, *27* (2), 558–566. https://doi.org/10.1107/S1600577520000776.

(8) Bon, V.; Senkovska, I.; Wallacher, D.; Heerwig, A.; Klein, N.; Zizak, I.; Feyerherm, R.; Dudzik, E.; Kaskel, S. In Situ Monitoring of Structural Changes during the Adsorption on Flexible Porous Coordination Polymers by X-Ray Powder Diffraction: Instrumentation and Experimental Results. *Microporous Mesoporous Mater.* **2014**, *188*, 190–195. https://doi.org/10.1016/j.micromeso.2013.12.024.

(9) Yakout, S. M. E.; Abdeltawab, A. A.; Elhindi, K.; Askalany, A. Uranium Dynamic Adsorption Breakthrough Curve onto Rice Straw Based Activated Carbon Using Bed Depth Service Time Model. **2018**, *13*, 9143–9157. https://doi.org/10.15376/biores.13.4.9143-9157.

(10) McGivern, W. S.; Nguyen, H. G. T.; Manion, J. A. Improved Apparatus for Dynamic Column-Breakthrough Measurements Relevant to Direct Air Capture of CO2. *Ind. Eng. Chem. Res.* **2023**, *62* (21), 8362–8372. https://doi.org/10.1021/acs.iecr.2c04050.

(11) Kresse, G.; Hafner, J. Ab Initio Molecular Dynamics for Liquid Metals. *Phys. Rev. B* **1993**, *47* (1), 558–561. https://doi.org/10.1103/PhysRevB.47.558.

(12) Grimme, S.; Antony, J.; Ehrlich, S.; Krieg, H. A Consistent and Accurate Ab Initio Parametrization of Density Functional Dispersion Correction (DFT-D) for the 94 Elements H-Pu. *J. Chem. Phys.* **2010**, *132* (15), 154104. https://doi.org/10.1063/1.3382344.

(13) Grimme, S.; Ehrlich, S.; Goerigk, L. Effect of the Damping Function in Dispersion Corrected Density Functional Theory. *J. Comput. Chem.* **2011**, *32* (7), 1456–1465. https://doi.org/10.1002/jcc.21759.

(14) *Bem2 Parameters*. Dokumentacja WCSS. https://man.e-science.pl/en/kdm/bem2/parametry (accessed 2025-11-21).

(15) *PLGrid*. https://www.plgrid.pl/ (accessed 2025-11-21).
(16) *RASPA – iRASPA*. https://iraspa.org/raspa/ (accessed 2025-11-21).
(17) Rappe, A. K.; Casewit, C. J.; Colwell, K. S.; Goddard, W. A. I.; Skiff, W. M. UFF, a Full Periodic Table Force Field for Molecular Mechanics and Molecular Dynamics Simulations. *J. Am. Chem. Soc.* **1992**, *114* (25), 10024–10035. https://doi.org/10.1021/ja00051a040.
(18) Mayo, S. L.; Olafson, B. D.; Goddard, W. A. DREIDING: A Generic Force Field for Molecular Simulations. *J. Phys. Chem.* **1990**, *94* (26), 8897–8909. https://doi.org/10.1021/j100389a010.
(19) *TraPPE: Transferable Potentials for Phase Equilibria Force Field*. http://trappe.oit.umn.edu/ (accessed 2025-11-21).
(20) *Matplotlib — Visualization with Python*. https://matplotlib.org/ (accessed 2025-11-21).
(21) Schneider, W. B.; Bistoni, G.; Sparta, M.; Saitow, M.; Riplinger, C.; Auer, A. A.; Neese, F. Decomposition of Intermolecular Interaction Energies within the Local Pair Natural Orbital Coupled Cluster Framework. *J. Chem. Theory Comput.* **2016**, *12* (10), 4778–4792. https://doi.org/10.1021/acs.jctc.6b00523.
(22) Bistoni, G. Finding Chemical Concepts in the Hilbert Space: Coupled Cluster Analyses of Noncovalent Interactions. *WIREs Comput. Mol. Sci.* **2020**, *10* (3), e1442. https://doi.org/10.1002/wcms.1442.
(23) Altun, A.; Izsák, R.; Bistoni, G. Local Energy Decomposition of Coupled-Cluster Interaction Energies: Interpretation, Benchmarks, and Comparison with Symmetry-Adapted Perturbation Theory. *Int. J. Quantum Chem.* **2021**, *121* (3), e26339. https://doi.org/10.1002/qua.26339.
(24) Neese, F. Software Update: The ORCA Program System—Version 6.0. *WIREs Comput. Mol. Sci.* **2025**, *15* (2), e70019. https://doi.org/10.1002/wcms.70019.
(25) Riplinger, C.; Pinski, P.; Becker, U.; Valeev, E. F.; Neese, F. Sparse Maps—A Systematic Infrastructure for Reduced-Scaling Electronic Structure Methods. II. Linear Scaling Domain Based Pair Natural Orbital Coupled Cluster Theory. *J. Chem. Phys.* **2016**, *144* (2), 024109. https://doi.org/10.1063/1.4939030.
(26) Riplinger, C.; Sandhoefer, B.; Hansen, A.; Neese, F. Natural Triple Excitations in Local Coupled Cluster Calculations with Pair Natural Orbitals. *J. Chem. Phys.* **2013**, *139* (13), 134101. https://doi.org/10.1063/1.4821834.
(27) Weigend, F.; Ahlrichs, R. Balanced Basis Sets of Split Valence, Triple Zeta Valence and Quadruple Zeta Valence Quality for H to Rn: Design and Assessment of Accuracy. *Phys. Chem. Chem. Phys.* **2005**, *7* (18), 3297–3305. https://doi.org/10.1039/b508541a.
(28) Hellweg, A.; Hättig, C.; Höfener, S.; Klopper, W. Optimized Accurate Auxiliary Basis Sets for RI-MP2 and RI-CC2 Calculations for the Atoms Rb to Rn. *Theor. Chem. Acc.* **2007**, *117* (4), 587–597. https://doi.org/10.1007/s00214-007-0250-5.
(29) Weigend, F. Accurate Coulomb-Fitting Basis Sets for H to Rn. *Phys. Chem. Chem. Phys.* **2006**, *8* (9), 1057–1065. https://doi.org/10.1039/b515623h.
(30) Helmich-Paris, B.; de Souza, B.; Neese, F.; Izsák, R. An Improved Chain of Spheres for Exchange Algorithm. *J. Chem. Phys.* **2021**, *155* (10), 104109. https://doi.org/10.1063/5.0058766.
(31) Grimme, S.; Hansen, A.; Ehlert, S.; Mewes, J.-M. r2SCAN-3c: A "Swiss Army Knife" Composite Electronic-Structure Method. *J. Chem. Phys.* **2021**, *154* (6), 064103. https://doi.org/10.1063/5.0040021.
(32) Wuttke, A.; Mata, R. A. Visualizing Dispersion Interactions through the Use of Local Orbital Spaces. *J. Comput. Chem.* **2017**, *38* (1), 15–23. https://doi.org/10.1002/jcc.24508.
(33) Altun, A.; Neese, F.; Bistoni, G. Effect of Electron Correlation on Intermolecular Interactions: A Pair Natural Orbitals Coupled Cluster Based Local Energy Decomposition Study. *J. Chem. Theory Comput.* **2019**, *15* (1), 215–228. https://doi.org/10.1021/acs.jctc.8b00915.
(34) Palmer, D. C.; Palmer, S. E. CrystalMaker, 2026.
(35) Pettersen, E. F.; Goddard, T. D.; Huang, C. C.; Meng, E. C.; Couch, G. S.; Croll, T. I.; Morris, J. H.; Ferrin, T. E. UCSF ChimeraX: Structure Visualization for Researchers, Educators, and Developers. *Protein Sci.* **2021**, *30* (1), 70–82. https://doi.org/10.1002/pro.3943.
(36) Roztocki, K.; Sobczak, S.; Smaruj, A.; Walczak, A.; Gołdyn, M.; Bon, V.; Kaskel, S.; Stefankiewicz, A. R. Tuning the Guest-Induced Spatiotemporal Response of Isostructural Dynamic Frameworks towards Efficient Gas Separation and Storage. *J. Mater. Chem. A* **2023**, *11* (35), 18646–18650. https://doi.org/10.1039/D3TA02167J.

(37) Sobczak, S. K.; Mazur, B.; Malinska, M.; Formalik, F.; Bon, V.; Khadiev, A.; Kaskel, S.; Kuchta, B.; Janiak, A. M.; Roztocki, K. How a Single Atom Influences the Spatiotemporal Response of Flexible MOFs: Insights from Theory and Experiment. *J. Am. Chem. Soc.* **2025**, *147* (25), 21575–21585. https://doi.org/10.1021/jacs.5c02552.
(38) Klein, N.; Henschel, A.; Kaskel, S. *N*-Butane Adsorption on Cu3(Btc)2 and MIL-101. *Microporous Mesoporous Mater.* **2010**, *129* (1), 238–242. https://doi.org/10.1016/j.micromeso.2009.09.022.
(39) Assen, A. H.; Belmabkhout, Y.; Adil, K.; Bhatt, P. M.; Xue, D.-X.; Jiang, H.; Eddaoudi, M. Ultra-Tuning of the Rare-Earth Fcu-MOF Aperture Size for Selective Molecular Exclusion of Branched Paraffins. *Angew. Chem. Int. Ed.* **2015**, *54* (48), 14353–14358. https://doi.org/10.1002/anie.201506345.
(40) Yang, M.; Wang, H.; Zuo, J. Y.; Deng, C.; Liu, B.; Chai, L.; Li, K.; Xiao, H.; Xiao, P.; Wang, X.; Chen, W.; Peng, X.; Han, Y.; Huang, Z.; Dong, B.; Sun, C.; Chen, G. Efficient Separation of Butane Isomers via ZIF-8 Slurry on Laboratory- and Pilot-Scale. *Nat. Commun.* **2022**, *13* (1), 4792. https://doi.org/10.1038/s41467-022-32418-6.
(41) Jiang, Y.-P.; Deng, C.; Liang, C.-X.; Peng, X.; Zhang, Z.; Zhao, L.; Wu, X.-C.; Zou, J.-S.; Jia, Y.; Cheng, M.-J.; Li, J.-H.; Zhang, J.; Peng, Y.-L.; Gao, M.; Chen, G.-J.; Zaworotko, M. J. Ideal Molecular Splitter with Object Adaptive for Benchmark N-Butylene/Iso-Butylene Separation. *ACS Mater. Lett.* **2024**, *6* (11), 4972–4979. https://doi.org/10.1021/acsmaterialslett.4c01584.
(42) Bon, V.; Klein, N.; Senkovska, I.; Heerwig, A.; Getzschmann, J.; Wallacher, D.; Zizak, I.; Brzhezinskaya, M.; Mueller, U.; Kaskel, S. Exceptional Adsorption-Induced Cluster and Network Deformation in the Flexible Metal–Organic Framework DUT-8(Ni) Observed by in Situ X-Ray Diffraction and EXAFS. *Phys. Chem. Chem. Phys.* **2015**, *17* (26), 17471–17479. https://doi.org/10.1039/c5cp02180d.
(43) Jiang, Z.; Wang, L.; Xue, W.; Zheng, M.; Guo, X.; Huang, H.; Zhong, C. Adenine-Based Metal–Organic Framework with 1 D Narrow Channel for Molecular-Sieving Separation of n-Butane and Isobutane. *Sep. Purif. Technol.* **2024**, *328*, 125025. https://doi.org/10.1016/j.seppur.2023.125025.
(44) Song, D.; Ji, Z.; Zou, S.; Li, H.; Zhou, Y.; Chen, C.; Wu, M.; Hong, M. Incorporation of Multiple Binding Sites into an Adaptive MOF for Sieving Butane Isomers. *Adv. Funct. Mater.* **2025**, *35* (9), 2416384. https://doi.org/10.1002/adfm.202416384.
(45) Polyukhov, D. M.; Krause, S.; Bon, V.; Poryvaev, A. S.; Kaskel, S.; Fedin, M. V. Structural Transitions of the Metal–Organic Framework DUT-49(Cu) upon Physi- and Chemisorption Studied by in Situ Electron Paramagnetic Resonance Spectroscopy. *J. Phys. Chem. Lett.* **2020**, *11* (15), 5856–5862. https://doi.org/10.1021/acs.jpclett.0c01705.
(46) Ni, S.; Li, Y.-T.; Xu, X.; Hou, S.; Lü, X.; Yang, Q.-Y. A Fluorinated Zinc-Based Metal-Organic Framework for Efficient Separation of Butane Isomers via Pore Engineering. *Small Methods* **2025**, *9* (8), 2500027. https://doi.org/10.1002/smtd.202500027.
(47) Liu, H.; He, Y.; Jiao, J.; Bai, D.; Chen, D.; Krishna, R.; Chen, B. A Porous Zirconium-Based Metal-Organic Framework with the Potential for the Separation of Butene Isomers. *Chem. – Eur. J.* **2016**, *22* (42), 14988–14997. https://doi.org/10.1002/chem.201602892.
(48) Chen, Z.; Feng, L.; Liu, L.; Bhatt, P. M.; Adil, K.; Emwas, A.-H.; Assen, A. H.; Belmabkhout, Y.; Han, Y.; Eddaoudi, M. Enhanced Separation of Butane Isomers via Defect Control in a Fumarate/Zirconium-Based Metal Organic Framework. *Langmuir* **2018**, *34* (48), 14546–14551. https://doi.org/10.1021/acs.langmuir.8b03085.
(49) Li, B.; Belmabkhout, Y.; Zhang, Y.; Bhatt, P. M.; He, H.; Zhang, D.; Han, Y.; Eddaoudi, M.; Perman, J. A.; Ma, S. From an Equilibrium Based MOF Adsorbent to a Kinetic Selective Carbon Molecular Sieve for Paraffin/Iso-Paraffin Separation. *Chem. Commun.* **2016**, *52* (96), 13897–13900. https://doi.org/10.1039/c6cc08008a.
(50) Liu, Y.-P.; Zhou, W.-X.; Li, J.-H.; Chen, Z.-N.; Xue, W.; Zhou, H.-L.; Lin, R.-B. Microporous Nickel Formate for Kinetic Separation of Butane Isomers. *Ind. Eng. Chem. Res.* **2025**, *64* (28), 14134–14140. https://doi.org/10.1021/acs.iecr.5c00450.
(51) Bon, V.; Busov, N.; Senkovska, I.; Bönisch, N.; Abylgazina, L.; Khadiev, A.; Novikov, D.; Kaskel, S. The Importance of Crystal Size for Breathing Kinetics in MIL-53(Al). *Chem. Commun.* **2022**, *58* (75), 10492–10495. https://doi.org/10.1039/d2cc02662g.

(52) Xu, L.; Ni, S.; Li, Y.-T.; Zhang, L.-P.; Zheng, S.-T.; Zhang, X.-T.; Liu, X.-Y.; Yang, Q.-Y. A Highly Stable Interpenetrating Cobalt-Based Metal–Organic Framework for n-Butane/i-Butane Separation. *Ind. Eng. Chem. Res.* **2026**, *65* (1), 882–889. https://doi.org/10.1021/acs.iecr.5c04518.
(53) Liu, Y.-P.; Li, J.-H.; Chen, Z.-N.; Xue, W.; Zhou, H.-L.; Lin, R.-B.; Chen, X.-M. Kinetic Separation of Butane Isomers Using a Formate Metal-Organic Framework. *ChemPlusChem* **2025**, *90* (4), e202400756. https://doi.org/10.1002/cplu.202400756.
(54) Ye, Z.-M.; Zhang, X.-W.; Liao, P.-Q.; Xie, Y.; Xu, Y.-T.; Zhang, X.-F.; Wang, C.; Liu, D.-X.; Huang, N.-Y.; Qiu, Z.-H.; Zhou, D.-D.; He, C.-T.; Zhang, J.-P. A Hydrogen-Bonded yet Hydrophobic Porous Molecular Crystal for Molecular-Sieving-like Separation of Butane and Isobutane. *Angew. Chem. Int. Ed.* **2020**, *59* (51), 23322–23328. https://doi.org/10.1002/anie.202011300.
